# Correction: Contexts for developing of national essential diagnostics lists. Lessons from a mixed-methods study of existing documents, stakeholders and decision making on tier-specific essential in-vitro diagnostics in African countries

**DOI:** 10.1371/journal.pgph.0002502

**Published:** 2023-10-11

**Authors:** The PLOS Global Public Health Staff

## Notice of Republication

This article was republished on July 12, 2023, to correct errors in the title. The publisher apologizes for the errors. Please download this article again to view the correct version. The originally published, uncorrected article and the republished, corrected articles are provided here for reference.

## Supporting information

S1 File Originally published, uncorrected article. (PDF)

S2 File Republished, corrected article. (PDF)
