# Supplementary material for: Contexts for developing of national essential diagnostics list. Lessons from a mixed-methods study of existing documents, stakeholders and decision making on tier-specific essential in-vitro diagnostics in African countries
Source: PLOS Glob Public Health. 2023 May 18;3(5):e0001893. doi: 10.1371/journal.pgph.0001893 (PMC10194858; doi:10.1371/journal.pgph.0001893)
Supplement: S1 Table — (PDF) [file pgph.0001893.s001.pdf]

S1 Table. Test Menus

Country	Name of test menu, with dates	URL (if available)
Nigeria	Nigeria National Essential Diagnostics List 2021	Not found online – Document shared
Benin	Paquet minimum d'activités Normes corrigé et Mis en forme (2009)	Not found online – Document shared
Botswana	Standardisation and Laboratory Logistics System Design for Botswana 2009	https://studylib.net/doc/18226387/standardization-and-laboratory-logistics-system-design-fo...
Burkina Faso	Normes en Matière de Laboratoires d'Analyses de Biologie Médicale Juillet 2009	Not found online – Document shared
Burundi	Les normes et standards des laboratoires de Biologie médicale au Burundi	https://afahobckpstorageaccount.blob.core.windows.net/afahobckpcontainer/production/files/Les_normes_et_standards_des_laboratoires_de_Biologie_m%C3%A9dicale_au_Burundi.pdf
Cameroon	Organisation des Laboratoires Suivant la Pyramide Sanitaire au Cameroun 2011	Not found online – Document shared
Côte d'Ivoire	Organisation Laboratoires Analyses Biologie Medicale (2008)	Not found online – Document shared
eSwatini	Essential health care package for Swaziland (2010)	https://extranet.who.int/countryplanningcycles/sites/default/files/planning_cycle_repository/swaziland/essential_health_care_package_for_swaziland.pdf
Ethiopia	The Master Plan for the Public Health Laboratory System 2009-2013	https://www.ephi.gov.et/images/downloads/Ethiopia%20Lab%20Master%20Plan_2nd%20Edition.pdf
Gabon	Health Sector Standards 2011	https://csgabon.info/file/f2/Normessante%2014072011.pdf
Gambia	Basic package test menu - Disease Specific Reference Laboratories (not dated)	Not found online – Document shared
Ghana	Development of a Tiered Laboratory System in Ghana (2021)<br>Laboratory Policy (2015)	Not found online – Document shared
Guinea	Politique Nationale de Biologie Médicale (2009)	https://portail.sante.gov.gn/wp-content/uploads/2020/04/29.-Politique-Nationale-de-Biologie-M%c3%a9dicale-ocr.pdf
Guinee Bissau	Lista de testes por nível (not dated)	Not found online – Document shared
Kenya	Kenya Essential Medical Laboratory Commodity List 2014	https://docplayer.net/43635108-Kenya-essential-medical-laboratory-commodity-list-2014.html
Liberia	Essential Package of Health Services (2011)	Not found online – Document shared

Malawi	Standardisation of Laboratory Tests, Techniques and Equipment 2009	https://pdf.usaid.gov/pdf_docs/Pnadr853.pdf
Mali	"Guide de Bonne Exécution des Analyses (GBEA) dans les laboratoires d'Analyses Médicales (2005)"	Not found online – Document shared
Niger	Paquet Minimum d'Activités des laboratoires par niveau de la pyramide sanitaire (not dated)	Not found online – Document shared
Senegal	Manuel des Techniques Laboratoire 2015	https://dirlabosn.com/wp-content/uploads/2020/02/manuel_SGQ.pdf
Sierra Leone	Basic Package of Essential Health Services (2015-2020)	https://mohs2017.files.wordpress.com/2017/06/gosl_2015_basic-package-of-essential-health-services-2015-2020.pdf
South Africa	Primary Health Care - Laboratory Handbook May 2018	https://knowledgehub.health.gov.za/elibrary/primary-health-care-phc-laboratory-handbook
Togo	"* Documents split by different laboratory sections (Anatomy, toxicology, Biology, Parasitology, Immunology etc) (2021)"	Not found online – Document shared
Uganda	Standard Test Menu, Techniques and List of Supplies for Health Laboratories in Uganda 2017-2020	https://www.cphl.go.ug/sites/default/files/2019-06/NSTMT%20TEST%20MENU.pdf
Tanzania	Standard Medical Laboratory Equipment Guideline 2018	Not found online – Document shared
Zimbabwe	Laboratory Harmonisation and Standardisation in Zimbabwe 2015	Not found online – Document shared
