# Supplementary material for: Contexts for developing of national essential diagnostics list. Lessons from a mixed-methods study of existing documents, stakeholders and decision making on tier-specific essential in-vitro diagnostics in African countries
Source: PLOS Glob Public Health. 2023 May 18;3(5):e0001893. doi: 10.1371/journal.pgph.0001893 (PMC10194858; doi:10.1371/journal.pgph.0001893)
Supplement: S2 Table — (PDF) [file pgph.0001893.s002.pdf]

Country	Document Name	Year	URL
Angola	iiep_aids_national_program_angola	1999-2002	Document shared
	angola_hivaids_en	2003-2008	https://gcwa.unaids.org/external-resource/angola-aids-national-strategic-plan-2003-2008
	PLANO ESTRATÉGICO NACIONAL PARA AS INFECÇÕES DE TRANSMISSÃO SEXUAL, VIH / SIDA	2003-2008	https://www.ilo.org/wcmsp5/groups/public/---ed_protect/---protrav/---ilo_aids/documents/legaldocument/wcms_173701.pdf
	Plano Estratégico Nacional Para o Controlo das Infecções de Transmissão Sexual, VIH e SIDA_2007-2010_fr	2007-2010	https://www.ilo.org/wcmsp5/groups/public/---ed_protect/---protrav/---ilo_aids/documents/legaldocument/wcms_126907.pdf
	fy-2019-angola-malaria-operational-plan	2019	https://d1u4sg1s9ptc4z.cloudfront.net/uploads/2021/03/fy-2019-angola-malaria-operational-plan.pdf
	02-Angola-Plano-de-Transição-da-Pólio-v-20-Abr-18	2018-2022	https://www.passeidireto.com/arquivo/76400133/02-angola-plano-de-transicao-da-polio-v-20-abr-18
	PLANO NACIONAL DE ,DESENVOLVIMENTO SANITARIO	2012-2025	https://extranet.who.int/mindbank/item/3460
Benin	Benin Strategic plan 2016 - 2020	2016-2020	Document shared
	Benin-Plan-National-de-Développement-Sanitaire-2018-2022	2018-2022	https://www.prb.org/wp-content/uploads/2020/06/Benin-Plan-National-de-D%C3%A9veloppement-Sanitaire-2018-2022.pdf
	Benin_PSN_TB_2015-2019	2015-2019	Document shared
	Benin-AIDS-National-Strategic-Plan-2008	2008	https://gcwa.unaids.org/external-resource/benin-aids-national-strategic-plan-2008
	Benin-Plan-Operationnel-de-Reduction-de-la-Mortalite-Maternelle-et-Neonatale-au-Benin	2018 - 2022	https://www.prb.org/wp-content/uploads/2020/06/Benin-Plan-Operationnel-de-Reduction-de-la-Mortalite-Maternelle-et-Neonatale-au-Benin.pdf
	Benin Plan_strategique_integre_lutte_contre_maladies_non_transmissibles_2014-2018	2014-2018	https://www.iccp-portal.org/system/files/plans/Benin%20Plan_strategique_integre_lutte_contre_maladies_non_transmissibles_2014-2018.pdf
	PLAN STRATEGIQUE INTEGRE DE LUTTE CONTRE LES MALADIES NON TRANSMISSIBLES 2019-2023	2019-2023	https://www.iccp-portal.org/system/files/plans/MLI_B3_s21_Plan%20Strat%C3%A9gique%20MNT%20VF%20230319.pdf
	PLAN D'ACTION NATIONAL DE LA SECURITE SANITAIRE DU BENIN	2019-2021	https://faolex.fao.org/docs/pdf/ben196357.pdf
	Document-de-Stratégie-Nationale-de-Réduction-de-la-Mortalité-Maternelle-et-Néonatale-au-Bénin-2006-2015	2006-2015	https://fr.readkong.com/page/document-de-strategie-nationale-de-reduction-de-la-1702259
	STRATEGIE NATIONALE MULTISECTORIELLE DE SANTE SEXUELLE ET DE LA REPRODUCTION DES ADOLESCENTS ET JEUN	2010-2020	https://www.prb.org/wp-content/uploads/2018/05/Strate%CC%81gie-Nationale-Multisectorielle-de-Sante%CC%81-Sexuelle-et-de-la-Reproduction-des-Adolescents-et-Jeunes-au-Be%CC%81nin-2010-2020.pdf
	ANNEXE 4 - PIC Plan Intégre de com SMNE-version finale	2014-2018	Document shared
	PSNIE BENIN _ 2020 - 2024 (PLAN STRATEGIQUE NATIONAL INTEGRE ORIENTE VERS L'ELIMINATION DU VIHSIDA, LA TU	2020 - 2024	Document shared
	fy-2016-benin-malaria-operational-plan	2016	https://d1u4sg1s9ptc4z.cloudfront.net/uploads/2021/03/fy-2016-benin-malaria-operational-plan.pdf
Botswana	revised_National_Health_Policy	2011	Document shared
	Standardization and Laboratory Logistics System Design for Botswana	2009	https://studylib.net/doc/18226387/standardization-and-laboratory-logistics-system-design-fo...
	Botswana Integrated Health Service Plan Final HLSP	2010-2020	https://www.medbox.org/document/integrated-health-service-plan-a-strategy-for-changing-the-health-sector-for-healthy-botswana-2010-2020#GO
	Malaria Strategic Plan Botswa2010-2015	2010-2015	https://endmalaria.org/sites/default/files/botswa2010-2015.pdf
	Botswana National Drug (Medicine) Policy	2005	https://www.moh.gov.bw/Publications/policies/Botswana%20National%20Drug%20(Medicine)%20Policy.pdf
	NATIONAL TUBERCULOSIS PROGRAM MANUAL botswana_tb	2007	https://www.medbox.org/document/botswana-national-tuberculosis-programme-manual#GO
	botswana_tb_hiv_policy_guidelines_(2011)	2011	https://www.tbonline.info/media/uploads/documents/botswana_tb:hiv_policy_guidelines_%282011%29.pdf
	Botswana Essential Health Service Plan HLSP	2010	https://www.moh.gov.bw/Publications/policies/Botswana%20EHSP%20HLSP.pdf
	Botswana Maternal Mortality Reduction Initiative	2015	https://pdf.usaid.gov/pdf_docs/PA00TCKG.pdf
	Botswana NCD Strategy Final	2018-2013	https://www.iccp-portal.org/system/files/plans/Botswana%20NCD%20Strategy%20Final.pdf
	Clinic standards	2013	https://www.moh.gov.bw/Publications/standards/Botswana%20National%20Health%20Quality%20Standards%20for%20Clinics/Botswana%20CLINIC%20Standards%20SE%206%20Primary%20Healthcare%20Services.pdf
	The National HIVAIDS Strategic Framework 2003-2009	2003-2009	https://extranet.who.int/countryplanningcycles/sites/default/files/country_docs/Botswana/botswana_national_hiv_aids_stragetic_framework_2003-2009.pdf
Burkina Faso	Burkina_Faso_Nat_Health_Strategy_2011-2020 Fr	2011-2020	https://www.childrenandaids.org/sites/default/files/2018-05/Burkina_Faso_Nat_Health_Strategy_2011-2020%20Fr.pdf
	PLAN STRATEGIQUE NATIONAL MULTISECTORIEL DE LUTTE CONTRE LES MALADIES NON TRANSMISSIBLES	2019-2021	Document shared
	Burkina Faso - Plan Stratégique intégré de lutte contre les maladies non transmissibles 2014-2018	2014-2018	https://www.iccp-portal.org/sites/default/files/plans/Burkina%20Faso%20-%20Plan%20Strat%C3%A9gique%20int%C3%A9gr%C3%A9%20de%20lutte%20contre%20les%20maladies%20non%20transmissibles%202014-2018.pdf
	Sante-Diabete_strategie_COVID19-Diabete_FR	2020	https://www.idf.org/component/attachments/?task=download&id=2178:Sante-Diabete_strategie_COVID19-Diabete_FR
	guide de bonne execution des analyses de biologie medicale au burkina faso	2009	https://www.studocu.com/row/document/universite-de-ouagadougou/hematologie/gbea-bf-2-guide-dexecution-des-examens-de-biologie-medicale/21664603
	cadre strategique de lutte contre le vih/sida 2001-2005	2001-2005	https://data.unaids.org/topics/nsp-library/nsp-africa/nsp_burkina_faso_2001-2005_fr.pdf
	PLAN STRATEGIQUE INTEGRE DE LUTTE CONTRE LES MALADIES NON TRANSMISSIBLES2016-2020	2016-2020	https://www.medbox.org/document/plan-strategique-integre-de-lutte-contre-les-maladies-non-transmissibles-burkina-faso#GO
	Cadre Strategique de Lutte Contre Le VIH/SIDA 2006-2010	2006-2010	https://www.ilo.org/dyn/natlex/docs/MONOGRAPH/111680/139299/F-1243072672/BFA-111680.pdf
	Plan-Stratégique-Santé-des-Adolescents-et-des-Jeunes-2015-2020.-Burkina-Faso	2015-2020	https://www.prb.org/wp-content/uploads/2018/05/Plan-Strate%CC%81gique-Sante%CC%81-des-Adolescents-et-des-Jeunes-2015-2020.-Burkina-Faso.pdf
	Normes_en_matiere_de_labo-analyse_biolmed_LABM_BF-2	2009	Document shared
	Burkina Faso_Plan stratégique de lutte contre le cancer 2013-2017	2013-2017	https://www.iccp-portal.org/system/files/plans/Burkina%20Faso_Plan%20strat%C3%A9gique%20de%20lutte%20contre%20le%20cancer%202013-2017.pdf
	fy-2019-burkina-faso-malaria-operational-plan	2019	https://d1u4sg1s9ptc4z.cloudfront.net/uploads/2021/03/fy-2019-burkina-faso-malaria-operational-plan.pdf
Burundi	Plan Stratégique National de Laboratoire 2015-2019	2015-2019	Document shared
	burundi_tb_2011-2015_fr	2011-2015	Document shared
	Plan Stratégique National de lutte contre le Paludisme 2018-2023	2018-2023	http://minisante.bi/wp-content/uploads/pnilp/Plan%20Strat%C3%A9gique%20National%20de%20lutte%20contre%20le%20Paludisme%20%202018-2023.pdf
	PLAN-STRATEGIQUE-NATIONAL-DE-LA-SANTE-DE-LA-REPRODUCTION-MATERNELLE-NEONATALE-INFANTILE-ET-DES-A	2019-2023	https://share-net-burundi.org/wp-content/uploads/2019/07/PLAN-STRATEGIQUE-NATIONAL-DE-LA-SANTE-DE-LA-REPRODUCTION-MATERNELLE-NEONATALE-INFANTILE-ET-DES-ADOLESCENTS.pdf
	Plan Stratégique National de lutte contre le VIH/SIDA 2007-2011	2007-2011	https://www.ilo.org/wcmsp5/groups/public/---ed_protect/---protrav/---ilo_aids/documents/legaldocument/wcms_126635.pdf
	WHO co-operation with Burundi	2016-2018	https://apps.who.int/iris/handle/10665/137042?locale-attribute=fr&
	PLAN STRATEGIQUE NATIONAL DE LUTTE CONTRE LES MALADIES CHRONIQUES NON TRANSMISSIBLES	2011-2015	https://www.iccp-portal.org/system/files/plans/BDI_B3_PS%20PNILMCNT%2029%2006%202011%5B1%5D.pdf
	PLAN D'ACTIVITES DES MALADIES CHRONIQUES NON TRANSMISSIBLES 2017	2017	https://www.iccp-portal.org/system/files/plans/BDI_B3_PAA%20FINALE%202017%20version%20%20%20electronique%20(2).pdf
	Burundi_Nat Plan AIDS_2014 fr	2014-2017	http://minisante.bi/wp-content/uploads/pnls/PSN-VIH%202014-2017.pdf
Cameroon	DIRECTIVES NATIONALES DE PREVENTION ET DE PRISE EN CHARGE DU VIH AU CAMEROUN	2014-2018	https://www.childrenandaids.org/sites/default/files/2017-05/Cameroon_National-Integrated-HIV-Guidelines2014.pdf
	Guide national de prise en charge des personnes vivant avec le VIHSIDA – Cameroun		https://www.aidsmap.com/sites/default/files/legacy/-v634770171201330000-file-1052501-guide-national-de-prise-en-charge-des-personnes-vivant-avec-le-vih-sida__e2-88-92-camerounpdf.png
	GuideBonneExecutionAnalyseBiologieMedicale_dpml_minsante_cameroun	2011	https://dpml.cm/images/Publications/GuideBonnePratique/GuideBonneExecutionAnalyseBiologieMedicale_dpml_minsante_cameroun.pdf
	ORGANISATION DES LABORATOIRES SUIVANT LA PYRAMIDE SANITAIRE AU CAMEROUN		Document shared
	Plan strategique de lutte contre la TB au Cameroun 2015 - 2019	2015 - 2019	https://www.pnlt.cm/index.php/documentation/plan-strategique-national/4-plan-strategique-national-2020-2024-du-programme-national-de-lutte-contre-la-tuberculose/file
	national tuberculosis strategic plan 2010	2010-2014	https://extranet.who.int/countryplanningcycles/sites/default/files/planning_cycle_repository/cameroon/nationaltuberculosisstrategicplan2010.pdf
	PLAN STRATEGIQUE NATIONAL DE LUTTE CONTRE LE VIH, LE SIDA ET LES IST 2014-2017	2014_2017	https://www.ilo.org/dyn/natlex/natlex4.detail?p_lang=fr&p_isn=99130&p_count=96544
	National Health Development Plan 2016-2020.Cameroon.	2016-2020	https://www.minsante.cm/site/?q=en/content/national-health-development-plan-nhdp-2016-2020
	Plan Stratégique National de Prévention et de Lutte contre le Cancer (PSNPLCa)	2020 - 2024	https://www.iccp-portal.org/system/files/plans/FINAL%20COPY%20PSNPLCa%20FRENCH.pdf
	FR_DOCUMENT_PLAN STRATEGIQUE NATIONAL DE SANTE NUMERIQUE_Réduit	2020 - 2024	https://www.minsante.cm/site/?q=en/content/plan-strat%C3%A9gique-national-de-sant%C3%A9-num%C3%A9rique-2020-2024
	HEALTH SECTOR STRATEGY 2016-2027	2016-2027	https://www.minsante.cm/site/sites/default/files/HSS_english_0.pdf
	PDRH- Etats des lieux des RHS_reduit_0	2013-2017	https://www.minsante.cm/site/?q=fr/content/pdrh-2013-2017-etat-des-lieux-et-diagnostic-des-ressources-humaines
	Cameroun-Plan Strategique National Paludisme_2019-2023	2019-2023	https://lnsp-cam.org/wp-content/uploads/2021/10/PSNLP-2019-2023-CONSOLIDE-TRANSMIS.pdf
	Manuel_de_campagne_de_sensibilisation_RHN_de_la_FID	2018	https://idf.org/images/Manuel_de_campagne_de_sensibilisation_RHN_de_la_FID.pdf
	Plan strategique de lutte contre la TB au Cameroun 2015 - 2019	2015-2019	Document shared
	Plan Strategic National du Develpmt labo 2018-2020 (1)	2018-2022	Document shared
Central African Repu	RCA-Plan-National-de-Developpement-Sanitaire-2006-2015	2006-2015	https://www.prb.org/wp-content/uploads/2020/06/RCA-Plan-National-de-Developpement-Sanitaire-2006-2015.pdf
	sep2007_PLAN_STRATEGIQUE_SYSTEMESSANTE_RCA_finale	2017	https://www.humanitarianresponse.info/fr/operations/central-african-republic/document/plan-strategique-de-renforcement-des-syst%C3%A8mes-de-sant%C3%A9
	normes_districts_sante_rca		Document shared
	CADRE STRATEGIQUE NATIONAL DE LUTTE CONTRE LE VIH ET LE SIDA 2012-2016	2012-2016	https://www.ilo.org/wcmsp5/groups/public/---ed_protect/---protrav/---ilo_aids/documents/legaldocument/wcms_301805.pdf
	Central_African_Republic_2003_Reproductive_Health	2003	Document shared
	msp-rca_politique_pnlp_version_finale	2016	https://www.humanitarianresponse.info/sites/www.humanitarianresponse.info/files/documents/files/msp-rca_politique_pnlp_version_finale.pdf
	9_rca_plan_strategique_palu_07_11	2007-2011	https://www.humanitarianresponse.info/sites/www.humanitarianresponse.info/files/documents/files/msp-rca_politique_pnlp_version_finale.pdf
	CAF_B3_Plan strat MNT 2015 2021 RCA 7 04 2015	2015-2021	https://www.sante.gouv.sn/sites/default/files/Plan%20Strat%C3%A9gique_DLMNT.pdf
	RCA_Malaria_Annexe 12_PSN paludisme 2018-2022 etendu 2023	2018-2023	https://scorecardhub.org/wp-content/uploads/2021/06/drc-plan-strategique-jules-corrige.pdf

Chad	Tchad-Politique-Nationale-de-Sante-2016-2030	2016-2030	https://www.prb.org/wp-content/uploads/2020/06/Tchad-Politique-Nationale-de-Sante-2016-2030.pdf
	PLAN NATIONAL DE DEVELOPPEMENTSANITAIRE (PNDS3 : 2018-2021)	2018-2021	https://www.prb.org/wp-content/uploads/2020/06/Tchad-Plan-National-de-Developpement-Sanitaire-2018-2021.pdf
	PLANSTRATEGIQUE NATIONALDERIPOSTE AU SIDA 2012-2015	2012-2015	https://www.ilo.org/dyn/natlex/docs/ELECTRONIC/111139/138421/F-1953807236/TCD-111139.pdf
	TCHAD_PLAN STRATEGIQUE NATIONAL 2019-2023	2019-2023	https://executiveboard.wfp.org/document_download/WFP-0000099504
Comoros	DOCUMENT DE STRATÉGIE NATIONALE DE PRÉVENTION ET DE LUTTE CONTRE LES MALADIES NON	2013	https://www.iccp-portal.org/system/files/plans/COM_B3_STRATEGIE%20MNT%20draft%20MARS%2013%20%281%29%20%281%29.pdf
	POLITIQUE NATIONALE DE SANTE (PNS) FEVRIER 2015	2005	https://www.ilo.org/dyn/natlex/docs/ELECTRONIC/111131/138412/F-734372038/COM-111131.pdf
	ASC_Santé_Plan-strategique-contre-paludisme_2007-2014	2007-2014	http://www.cgp.km/doc/Plan_Strategique_de_lutte_contre_le_paludisme_2007-2014.pdf
	PLAN NATIONAL DE DEVELOPPEMENT SANITAIRE 2010-2014	2010-2015	https://faolex.fao.org/docs/pdf/com200553.pdf
	Programme National de lutte contre la Tuberculose et la Lèpre COMORES Nov 2011	2011	Document shared
	ASC_Santé_PSN-contre-VIH-SIDA_2015-2019	2015-2019	Document shared
	Comoros_Nat Strat Plan HIV_2011-2015 fr	2011-2015	https://www.childrenandaids.org/sites/default/files/2018-05/Comoros_Nat%20Strat%20Plan%20HIV_2011-2015%20fr.pdf
Congo	Congo_Nat Strat Framework_2014-2018 Fr	2014-2018	https://www.childrenandaids.org/sites/default/files/2018-05/Congo_Nat%20Strat%20Framework_2014-2018%20Fr.pdf
Côte d'Ivoire	PSN_TB-CI_ 2016-2020_Plan Principal	2016-2020	https://allianceciv.org/alliance/pdf/PSN_TB-CI_%202016-2020_Plan%20Principal.pdf
	National Malaria strategic plan	2018-2023	https://d1u4sg1s9ptc4z.cloudfront.net/uploads/2021/03/fy-2018-cote-divoire-malaria-operational-plan.pdf
	Plan-Stratégique-National-de-la-Santé-des-Adolescents-et-des-Jeunes-2016-2020.-Côte-d'Ivoire	2016-2020	https://www.prb.org/plan-strategique-national-de-la-sante-des-adolescents-et-des-jeunes-2016-2020-co%CC%82te-divoire-pdf/
	PLAN NATIONAL DE DEVELOPPEMENT SANITAIRE 2016-2020	2016-2020	https://www.childrenandaids.org/sites/default/files/2017-11/pnds_2016-2020.pdf
	Policy of health and Public hygiene	2016	Document shared
	Plan d'action chaque nouveau né 2018-2020	2018-2020	https://www.unicef.org/cotedivoire/media/2546/file/Plan%20d'action%20chaque%20nouveau%20n%C3%A9%202018-2020.pdf
	Non-communicable diseases plan	2015-2019	Document shared
	Cote-d-Ivoire_Nat Strat Plan HIV_2016-2020 fr	2016-2020	https://www.unaids.org/sites/default/files/country/documents/CIV_2017_countryreport.pdf
Democratic Republic	DRC-National-Strategic-Plan-Against-HIV-2014-2017	2014-2017	https://www.childrenandaids.org/sites/default/files/2017-05/DRC-National-Strategic-Plan-Against-HIV-2014-2017.pdf
	Guide plateau technique par niveau	2021	Document shared
	National Multisectoral Strategic Plan_2014-2020_english_20140916'	2014-2020	Document shared
	National strategic plan to fight Malaria2016-2020	2016-2020	https://scorecardhub.org/wp-content/uploads/2021/06/drc-plan-strategique-jules-corrige.pdf
	5_PolitiqueNationaleDeSanteDeLaReproduction_Juillet_2008	2008	http://familyplanning-drc.net/docs/5_PolitiqueNationaleDeSanteDeLaReproduction_Juillet_2008.pdf
	PLAN-STRATEGIQUE-NATIONAL-CSU_FINANCEMENT	2020-2030	Document shared
	Joint Mission of the United Nations Interagency Task Force on the Prevention and Control of Noncommunicable Diseas	2015	https://apps.who.int/iris/bitstream/handle/10665/332177/WHO-NMH-NMA-17.89-eng.pdf
	malaria_plan_strategique_2007_2011	2007_2011	https://www.nationalplanningcycles.org/sites/default/files/planning_cycle_repository/democratic_republic_of_congo/malaria_plan_strategique_2007_2011.pdf
	normes-zs-actualisees-juillet-2012x	2012	https://www.prb.org/wp-content/uploads/2020/06/RDC-Plan-National-de-Developpement-Sanitaire-2016-2020.pdf
Djibouti	DJIBOUTI-Plan strategique national Paludisme 2020-2024	2020-2024	https://erc.undp.org/evaluation/managementresponses/keyaction/documents/download/3685
	Rapport TB Djibouti final version	2019	Document shared
Egypt	HIV Strategic Framework Ministry of Health Egypt	2012-2016	Document shared
Eritrea	eritrea_health_strategic_plan	2010-2014	Document shared
Eswatini	Eswatini_The National Multisectoral HIV and AIDS Strategic Framework-2018-2023_final	2018-2023	Document shared
	National-Health-Ssector-Strategic--Plan-II-Final-draft--March-2015-_edited_	2014-2018	Document shared
	national framework to stop new hiv infections2015	2011-2015	Document shared
	Swaziland National Malaria Elimination Policy	2010	https://endmalaria.org/sites/default/files/swaziland2010.pdf
	essential health care package for swaziland	2010	https://extranet.who.int/countryplanningcycles/sites/default/files/planning_cycle_repository/swaziland/essential_health_care_package_for_swaziland.pdf
	standard treatment guidelines and Essential Medicines List	2012	https://www.medbox.org/pdf/5e148832db60a2044c2d2d3e
	National health sector strategic plan	2008-2013	https://www.fao.org/faolex/results/details/en/c/LEX-FAOC187342/
Ethiopia	ethiopia_health_1993_en	1993	https://www.cmpethiopia.org/media/health_policy_of_ethiopia_1993
	ethiopia-health-sector-development-planhsdp-iii	2005-2009	https://extranet.who.int/countryplanningcycles/sites/default/files/planning_cycle_repository/ethiopia/ethiopia-health-sector-development-planhsdp-iii.pdf
	ethiopia_malaria_national_strategic_plan_2011-2015_130810	2011-2015	https://extranet.who.int/countryplanningcycles/sites/default/files/country_docs/Ethiopia/ethiopia_malaria_national_strategic_plan_2011-2015_130810.pdf
	Ethiopia_Nat Strat Plan HIV_2015-2020	2015-2020	https://www.childrenandaids.org/sites/default/files/2018-05/Ethiopia_Nat%20Strat%20Plan%20HIV_2015-2020.pdf
	Ethiopia - National Strategic Plan Tuberculosis and Leprosy Control 2013-2020	2013-2020	https://www.afro.who.int/publications/national-strategic-plan-tuberculosis-and-leprosy-control-2006-2013-ec-201314-2020
	Health-Policy	1993	https://faolex.fao.org/docs/pdf/eth174474.pdf
Equitorial Guinea	Ameliorer_le_diagnostic_du_paludisme_02		https://www.rural21.com/fileadmin/_migrated/content_uploads/Ameliorer_le_diagnostic_du_paludisme_02.pdf
	Cadre strategique Guinee Equatoriale final	2001-2005	Document shared
Gabon	Gabon_PSN_ 2018-2021	2018-2021	Document shared
	normes santé 2011 (1)	2011	https://csgabon.info/file/f2/Normessante%2014072011.pdf
Gambia	Gambia MNH Roadmap	2005-2015	Document shared
	Gambia National Policy on HIV and AIDS	2014-2020	https://www.childrenandaids.org/sites/default/files/2018-05/The%20Gambia_Nat%20Guidelines%20HIVAIDS_2014-2020.pdf
	Gambia-National-Health-Policy_2012-2020	2012-2020	https://p4h.world/en/node/12245#:~:text=This%20national%20health%20policy%20provided,high%20morbidity%20and%20mortality%20rates.
Ghana	National Health Policy_12.07.2020.pdf-13072020-FINAL	2020	https://www.moh.gov.gh/wp-content/uploads/2020/07/NHP_12.07.2020.pdf-13072020-FINAL.pdf
	ghana health sector tb strategic plan 2015-2020_final	2015-2020	https://www.ccmghana.net/images/PRs/NTP/TB-health-sector-plan-2015-2020.compressed.pdf
	national_tb_health_sector_strategic_plan_2009-2013_2	2009-2013	https://www.tbonline.info/media/uploads/documents/national_tb_health_sector_strategic_plan_2009-2013_2.pdf
	Ghana National HIV and AIDS Strategic Plan 2016	2016-2020	https://www.childrenandaids.org/sites/default/files/2017-07/Ghana%20National%20HIV%20and%20AIDS%20Strategic%20Plan%202016.pdf
	Ghana Malaria NATIONAL STRATEGIC PLAN	2021-2025	Document shared
	fy-2018-ghana-malaria-operational-plan	2018	https://d1u4sg1s9ptc4z.cloudfront.net/uploads/2021/03/fy-2018-ghana-malaria-operational-plan.pdf
Djibouti	DJIBOUTI-Plan strategique national Paludisme 2020-2024	2020-2024	https://erc.undp.org/evaluation/managementresponses/keyaction/documents/download/3685
Guinea	Guinea_Nat Stat Framework HIV_2013-2017 fr	2013-2017	https://www.invest.gov.gn/document/vih-plan-strategique-2013-2017
	fy-2018-guinea-malaria-operational-plan	2018	https://d1u4sg1s9ptc4z.cloudfront.net/uploads/2021/03/fy-2018-guinea-malaria-operational-plan.pdf
	PNG-National Malaria Straetgic Plan (2014-2018)	2014-2018	https://apmen.org/sites/default/files/all_resources/PNG-National%20Malaria%20Straetgic%20Plan%20%282014-2018%29.pdf
	Plan-Comm 2018-2020 - 10 07 2019-VF- validé	2018-2022	Document shared
	plan-strategique-national-de-lutte-contre-le-paludisme-2013-2017	2013-2017	https://www.invest.gov.gn/document/plan-strategique-national-de-lutte-contre-le-paludisme-2013-2017#:~:text=Le%20but%20du%20Plan%20Strat%C3%A9gique,d'ici%20la%20fin%202017.&text=Pour%20atteindre%20ces%20objectifs%20des,par%20an%20ont%20%C3%A9t%C3%A9%20d%C3%A9finis.
	DNL DOC NORMES INFRA & EQPT LABO 161019 23H27 (1) draft final	2019	Document shared
	Plan-Stratégique-National-de-la-Santé-Maternelle-du-Nouveau-né-de-l'Enfant-de-l'Adolescent-et-des-Jeunes-2016-202	2016-2020	https://www.prb.org/plan-strategique-national-de-la-sante-maternelle-du-nouveau-ne-de-lenfant-de-ladolescent-et-des-jeunes-2016-2020-guinee-pdf/
	Extrait Loi N°L94_012_CTRN_Legislation Pharmaceutique_Des Analyses Biomédicales	2021	Document shared
Guinea-Bissau	Guinea-Bissau_Nat Strat Plan HIV_2007-2011	2007-2011	https://www.childrenandaids.org/node/530
Kenya	Kenya Essential Medical Laboratory Commodity List 2014	2014	https://docplayer.net/43635108-Kenya-essential-medical-laboratory-commodity-list-2014.html
	Kenya Aids Strategic Framework 2020_2025	2020_2025	https://nsdcc.go.ke/kenya-aids-strategic-framework-kasf-ii/#:~:text=The%20second%20Kenya%20AIDS%20Strategic,presentation%20for%20ease%20of%20implementation.
	kenya_national_strategic_plan_on_tuberculosis_leprosy	2015-2018	http://publications.universalhealth2030.org/uploads/kenya_national_strategic_plan_on_tuberculosis_leprosy.pdf
	National Strategic Plan for TB 2019 - 2023	2019 - 2023	https://www.chskenya.org/wp-content/uploads/2019/04/NSP-Executive-Summary_CH.pdf
	Kenya-Non-Communicable-Disease-NCD-Strategic-Plan-2021-2025	2021-2025	https://www.health.go.ke/wp-content/uploads/2021/07/Kenya-Non-Communicable-Disease-NCD-Strategic-Plan-2021-2025.pdf
	Kenya-Investment-Case - MNCH	2016	Document shared
	annual_moh_final_work_plan_2014-2015_5.07._2014-11	2014-2015	http://publications.universalhealth2030.org/uploads/annual_moh_final_work_plan_2014-2015_5.07._2014-11.pdf
	Tender-for-supply-of-Laboratory-Equipment-for-UHC-2	2018-2019	Document shared
	KTSWSSP_-_Revised_Procurement_Plan__for_RVWSB_Cluster-January_2019	2019	Document shared
	NRM-_Maternal_Health	2010	http://guidelines.health.go.ke:8000/media/NRM-_Maternal_Health.pdf
	Kenya-Malaria-Strategy-2019-2023	2019-2023	http://fountainafrica.org/wp-content/uploads/2020/01/Kenya-Malaria-Strategy-2019-2023.pdf

	Kenya_National_Diabetes_Strategy	2010-2015	https://www.worlddiabetesfoundation.org/sites/default/files/WDF09-436%20Kenya%20National%20Diabetes%20Strategy%202010-2015%20%20-%20Complete.pdf
	Kenya_health_policy_2014_to_2030	2014-2030	http://publications.universalhealth2030.org/uploads/kenya_health_policy_2014_to_2030.pdf
	Breast-Cancer-Screening-and-Early-Diagnosis-Action-Plan-2021-2025	2021-2025	https://www.health.go.ke/wp-content/uploads/2021/10/Breast-Cancer-Screening-and-Early-Diagnosis-Action-Plan-2021-2025.pdf
	Norms_and_Standards_for_Health_Service_Delivery_2006	2006	http://guidelines.health.go.ke:8000/media/Norms_and_Standards_for_Health_Service_Delivery_2006.pdf
Lesotho	LSO_B3_Endorsed NCD Stratergic Plan - Copy	2014-2020	https://www.iccp-portal.org/system/files/plans/LSO_B3_Endorsed%20NCD%20Stratergic%20Plan%20-%20Copy.pdf
	Lesotho_Nat Health Strat Plan_2017-2022	2017-2022	https://www.childrenandaids.org/sites/default/files/2018-05/Lesotho_Nat%20Health%20Strat%20Plan_2017-2022.pdf
	National AIDS strategic plan	2001-2004	https://www.prepwatch.org/resources/national-hiv-aids-strategic-plan-2018-19-2022-23/
	Lesotho - Maternal Health Oct 20-final	2013	https://www.undp.org/sites/g/files/zskgke326/files/publications/Lesotho%20-%20Oct%2020-final.pdf
	Lesotho_TB		Document shared
Liberia	Liberia National Malaria Communication Strategy 2016-2020	2016-2020	Document shared
	Liberia NATIONAL HIV & AIDS STRATEGIC PLAN 2015-2020 Final _Authorized_ OK	2015-2020	https://www.childrenandaids.org/node/444#:~:text=The%20NSP%20aims%20to%20reduce,positive%20mothers%20on%20lifelong%20antiretroviral
	Liberia National Leprosy and Tuberculosis Strategic Plan 2014 - 2018	2014-2018	https://lcm.org.lr/wp-content/uploads/2021/09/TB-and-Leprosy-Strategic-Plan-2014-2018-consolidated-1-1.pdf
	liberia_maternal and newborn health_roadmap	2007	Document shared
	Liberia Family Planning Costed Implementation Plan: 2018-2022	2018-2022	http://www.healthpolicyplus.com/ns/pubs/10246-11503_LiberiaCIPBrief.pdf
	Other/pandemic/ Labs investment_plan_for_building_a_resilient_health_system		Document shared
	updated HIV treatment guidleines	2020	https://au.int/web/sites/default/files/newsevents/workingdocuments/27027-wd-liberia-_investment_plan_for_building_a_resilient_health_system.pdf
	Liberia national health and social welfare		https://faolex.fao.org/docs/pdf/lbr204463.pdf
	Liberia mental health Policy and strategy	2016-2021	https://www.afro.who.int/sites/default/files/2017-06/Mental%20Health%20Policy%20and%20Strategic%20Plan%20for%20Liberia%202016%20-%202021%20.pdf
	IHR-PVS NATIONAL BRIDGING WORKSHOP FOR LIBERIA		https://extranet.who.int/sph/sites/default/files/NBW%20Liberia-Final%20report.pdf
	LIBERIA_Negleted Tropical Diseases_Master_Plan_2016_2020	2016-2020	https://espen.afro.who.int/system/files/content/resources/LIBERIA_NTD_Master_Plan_2016_2020.pdf
	Liberia-National Standard Therapeutic Guidelines and Essential	2017	https://pdf.usaid.gov/pdf_docs/PA00JFTW.pdf
	[LIBERIA] Essential Package of Health Services (2011)	2011	https://www.hfgproject.org/essential-package-of-health-services-country-snapshot-liberia/
	Liberia-EPI-FP-Integration-Study-Report	2018	Document shared
	TERMS OF REFERENCE THE NATIONAL STANDARDS LABORATORY		https://www.fao.org/3/ca7509en/ca7509en.pdf
	Medicines List -EML-2nd-Edition-2017		https://www.medbox.org/pdf/5e148832db60a2044c2d4cf5
Madagascar	17365-17659_PSNRSC	2019-2030	http://www.healthpolicyplus.com/ns/pubs/17365-17659_PSNRSC.pdf
Malawi	Malawi 02017 - 2022 National Laboratory Strategic Plan Approved Version June 2018	2017 - 2022	https://extranet.who.int/countryplanningcycles/sites/default/files/planning_cycle_repository/malawi/health_sector_strategic_plan_ii_030417_smt_dps.pdf
	Malawi Malaria Strategic Plan 2017	2017 - 2022	https://d1u4sg1s9ptc4z.cloudfront.net/uploads/2021/03/fy-2017-malawi-malaria-operational-plan.pdf
	Malawi-National-HIV-AIDS-Strategic-Plan-2015-2020	2015-2020	https://www.childrenandaids.org/sites/default/files/2017-11/Malawi-National-HIV-AIDS-Strategic-Plan-2015-2020.pdf
	Malawi National Laboratory Policy Approved Version 13-03-2018.	2017	Document shared
	Malawi Revised Malaria Treatment Guidelines 5th Edition 2020 _ Final - Signed[94]	2020	Document shared
	Malawi National Stategic plan tuberculosis and leprosy	2021-2025	https://nkhokwe.kuhes.ac.mw/handle/20.500.12845/200#:~:text=The%20Plan%20seeks%20to%20further,TB%2FHIV%20collaborative%20activities%20and
	Malawi Updated escp (COVID and pandemic preparedness)	2021	Document shared
	Health Sector Strategic Plan	2011-2016	https://www.resakss.org/sites/default/files/Malawi%20MH%202011%20Malawi%20Health%20Sector%20Strategic%20Plan%202011%20-%202016.pdf
	Malawi ENAP - infant mortality reduction		https://cdn.who.int/media/docs/default-source/mca-documents/nbh/enap-country-progress-tracking-report-2015-2016-v2.pdf?sfvrsn=41945d17_1&download=true
	national community strategy	2017 - 2022	https://chwcentral.org/resources/national-community-health-strategy-malawi/#:~:text=The%20main%20focus%20of%20this,supplies%2C%20transportation%2C%20and%20infrastructure.
	Malawi Lab standardization	2009	https://pdf.usaid.gov/pdf_docs/Pnadr853.pdf
	TB Laboratory Netwot Operational Plan_Malawi 26 April	2021-2025	Document shared
Mali	MLI_B3_Plan strategique MNT adopté en CM le 22 octobre 2014	2015-2019	https://www.iccp-portal.org/system/files/plans/MLI_B3_Plan%20strategique%20MNT%20adopt%C3%A9%20en%20CM%20le%2022%20octobre%20%20%202014.pdf
	MH_StrategicPlan_2021	2017-2021	https://malihealth.org/wp-content/uploads/2018/04/MH_StrategicPlan_2021.pdf
Mauritania	projet de preparation de reponse strategique contre la COVID 19	2020	Document shared
	VIH	2012-2020	Document shared
	health technology national policy	2005-2015	Document shared
	Plan Nationale de Developmentment Sanitaire	2012-2020	Document shared
Mauritius	Form_B_indicative_procurement_plan_final	2021-2022	Document shared
	NASA (National AIDS Spending Assessment)	2018	https://health.govmu.org/Documents/Legislations/Documents/NASA%20Report%202018%2017%20AUG%202020%20%282%29.pdf
	Mauritius TB manual	2018	https://health.govmu.org/Documents/Departments-Hospitals/Departments/Documents/MANAGEMENT%20PROTOCOL%20FOR%20TUBERCULOSIS%20IN%20MAURITIUS%202018%20Final.pdf
	sexual and reporoductive health strategy and plan	2009-2015	https://healtheducationresources.unesco.org/sites/default/files/resources/mauritius_sexual_reproductive_health_strategy_plan_2009-2015.pdf
	National plan for COVID 19 resurgence	2021	https://health.govmu.org/Documents/Main%20Page/Communicable_Diseases/NATIONAL%20RESPONSE%20AND%20CONTINGENCY%20PLAN%20IN%20THE%20EVENTUALITY%20OF%20A%20RESURGENCE%20OF%20COVID-19%20CIRCULATION.pdf
	NATIONAL ACTION PLAN ON ANTIMICROBIAL RESISTANCE	2017-2021	https://www.who.int/publications/m/item/mauritius-national-action-plan-on-antimicrobial-resistance
	NATIONAL ACTION PLAN FOR THE PREVENTION AND CONTROL OF PLAGUE	2021	https://health.govmu.org/Documents/Main%20Page/Communicable_Diseases/NATIONAL%20ACTION%20PLAN%20FOR%20THE%20PREVENTION%20AND%20CONTROL%20OF%20PLAGUE.pdf
	national preparedness paln for Ebola virus	2014	https://health.govmu.org/Documents/Main%20Page/Communicable_Diseases/NATIONAL%20PREPAREDNESS%20PLAN%20ON%20EBOLA%20VIRUS%20DISEASE.pdf
	Health Sector Strategic Plan Final 15 September 2020	2020-2024	https://health.govmu.org/Communique/HSSP%20Final%2015%20September%202020.pdf
Mozambique	mozambique_-_health_sector_strategic_plan_-_2014-2019	2014-2019	https://extranet.who.int/countryplanningcycles/sites/default/files/planning_cycle_repository/mozambique/mozambique_-_health_sector_strategic_plan_-_2014-2019.pdf
	Plano Estratégico_PNCT 2008-2012	2008-2012	https://www.scribd.com/document/490688106/Plano-Estrategico-PNCT-2008-2012-2-pdf
	Maternal and Child Health Integrated Program (MCHIP)	2015	https://pdf.usaid.gov/pdf_docs/PA00JXHJ.pdf
	RAPID ASSESSMENT PROTOCOL FOR INSULIN ACCESS IN MOZAMBIQUE	2009	https://www.access2insulin.org/uploads/4/9/1/0/4910107/mozambique_rapia_report_2009.pdf
	Implementing Laboratory Quality Management Systems in Mozambique	2011-12	https://core.ac.uk/download/pdf/144171747.pdf
	Country Operational Plan for HIV	2021	https://www.state.gov/wp-content/uploads/2022/09/Mozambique-COP22-SDS-.pdf
	NCD Joint programming mission, 2-6 November 2015	2015	https://apps.who.int/iris/bitstream/handle/10665/275774/WHO-NMH-NMA-18.79-eng.pdf?sequence=1&isAllowed=y
	NATIONAL MALARIA CONTROL PROGRAMME	2017-2022	https://pdf.usaid.gov/pdf_docs/PA00W8CM.pdf
Namibia	Country Cooperation Strategy Namibia - 07 August 2019	2018-2022	https://www.afro.who.int/sites/default/files/2019-12/CCS%20Namibia%20-%2007%20August%202019.pdf
	Namibia Malaria Strategic Plan 2010-2016	2010-2016	https://endmalaria.org/sites/default/files/namibia2010-2016.pdf
	National Strategic Framework for HIV 2017_2022	2017_2022	https://hivpreventioncoalition.unaids.org/country-action/namibia-hiv-aids-national-strategic-framework/
	National-Guidelines-for-the-Management-of-Tuberculosis-Fourth-Edition-2019	2019	https://www.mhss.gov.na/documents/146502/1042285/National+Guidelines+for+the+Management+of+Tuberculosis%2C+Fourth+Edition+2019-2.pdf/c3676019-3205-4ed8-50dd-4fb301c3fda1?t=1657527637226&download=true
	namibia_national_health_policy_framework_2010-2020	2010-2020	https://extranet.who.int/countryplanningcycles/sites/default/files/country_docs/Namibia/namibia_national_health_policy_framework_2010-2020.pdf
	Namibia National Supply Chain Assessment Results	2013	https://www.medbox.org/document/namibia-national-supply-chain-assessment-results#GO
	NAMIBIA NATIONAL MULTISECTORAL STRATEGIC PLAN FOR PREVENTION AND CONTROL OF NCDs		https://www.iccp-portal.org/system/files/plans/NAMIBIA%20NATIONAL%20MULTISECTORAL%20STRATEGIC%20PLAN%20FOR%20PREVENTION%20AND%20CONTROL%20OF%20NCDs.pdf
	National Human Resources for Health Strategic Plan	2020-2030	https://pdf.usaid.gov/pdf_docs/PA00X19S.pdf
	Establishing a Public Health Laboratory System	2010-2020	https://www.aphl.org/conferences/proceedings/Documents/2014/Annual-Meeting/16%20Kaura.pdf
	Namibia Health Facility Cencus	2020-2030	https://shopsplusproject.org/sites/default/files/resources/Namibia%20Private%20Health%20Providers%20and%20Facilities%20Census%20Results.pdf
Niger	Niger_Nat Strat Plan HIV_2008-2012	2008-2012	https://www.childrenandaids.org/sites/default/files/2018-05/Niger_Nat%20Strat%20Plan%20HIV_2008-2012.pdf
	NER_B3_Plan Stratégique PNLMNT Niger	2012	https://www.iccp-portal.org/system/files/plans/NER_B3_Plan%20Strat%C3%A9gique%20PNLMNT%20Niger.pdf
	PSN_NIGER_Malaria_2017_2021_2_12_2016	2017-2021	https://extranet.who.int/nutrition/gina/sites/default/filesstore/NER%202019%20PLan%20strat%C3%A9gique%20national%20MNT.pdf
Nigeria	RevisedNationalHealthPolicyDocument	2004	https://ngfrepository.org.ng:8443/handle/123456789/3178
	NATIONAL-HIV-AND-AIDS-STRATEGIC-FRAMEWORK	2017-2021	https://www.aidsdatahub.org/resource/national-hiv-strategic-plan-2021-2026#:~:text=The%20National%20HIV%20Strategic%20Plan,all%20people%20living%20with%20HIV.
	Nigeria_National Malaria Strategic Plan2020	2014-2020	https://www.health.gov.ng/doc/NMEP-Strategic-Plan.pdf
	USAID Global Health Supply Chain Program-Procurement Supply Management (GHSC-PSM) Nigeria	2020	https://www.ghsupplychain.org/country-profile/nigeria
	Child Survival in Nigeria: Situation, Response, and Prospects	2002	http://www.policyproject.com/pubs/countryreports/nig_csrevised.pdf
	National Policy Strategic Plan NCD	2013	https://www.iccp-portal.org/national-policy-and-strategic-plan-action-prevention-and-control-non-communicable-diseases-ncds

	Nigeria National Essential Diagnostics List		Document shared
	SECOND NATIONAL STRATEGIC HEALTH DEVELOPMENT PLAN	2018-2022	https://ngfrepository.org.ng:8443/jspui/bitstream/123456789/3283/1/SECOND%20NATIONAL%20STRATEGIC%20HEALTH%20DEVELOPMENT%20PLAN%202018%20%E2%80%93%202022.pdf
	TB CRG Action Plan Nigeria	2021-2025	https://stoptb.org/assets/documents/communities/CRG/TB%20CRG%20Action%20Plan%20Nigeria.pdf
	National Guidelines for Setting up Medical Lab in Nigeria-1	2012	http://www.mlscn-as.org/document/National%20Guidelines%20for%20Setting%20up%20Medical%20Lab%20in%20Nigeria-1.pdf
Réunion	PRS_plan_strategique_24_07.pdf	2012-2016	https://www.lareunion.ars.sante.fr/prs-2012-2016-5
	Consultation on Technical and Operational Recommendations for Clinical Laboratory Testing Harmonization and Standa	2008	file:///Users/ndione/Downloads/consultation-on-technical-and-operational-recommendations.pdf
	Rwanda Strategic Plan for HIV Extended to 2020	2013-2020	https://rbc.gov.rw/IMG/pdf/rwanda_hiv_aids_2020_and_2030_targets.pdf
	Tuberculosis National Strategic Plan	2013-2018	https://rbc.gov.rw/fileadmin/user_upload/report2019/3%20%20Extended%20TB%20NSP%202018-2020_March_2017.pdf
	Republic of Rwanda National Community Health Policy	2020-2024	https://www.advancingpartners.org/sites/default/files/projects/policies/chp_rwanda_2008.pdf
	pdf_approved_procurement_plan_2019-2020	2019-20	https://mpe.gov.na/documents/105739/105862/Annual+Procurement+Plan+2019-2020.pdf/650c9fd5-5eea-5543-654f-5ace901f9325
Rwanda	Strategic NCDs framework and targets settings in Rwanda	7/7/2015	http://ncdsynergies.org/wp-content/uploads/2015/07/Rwanda-Target-Setting_Paolo-dAci-NCDsynergies.pdf
	Rwanda: Harmonized LMIS System Design Review and SOP/Curriculum Development	date not available	https://publications.jsi.com/JSIInternet/Inc/Common/_download_pub.cfm?id=17421&lid=3
	Trends in MCH-RWANDA	7/7/2015	https://dhsprogram.com/pubs/pdf/FA108/FA108.pdf
	Key Findings on Family Planning, Maternal and Child Health, and Malaria	7/1/1905	Document shared
	Obesity-MOH Clinical Practice Guidelines_main	7/9/1905	Document shared
	RWANDA MALARIA STRATEGIC PLAN	2020-2024	https://d1u4sg1s9ptc4z.cloudfront.net/uploads/2022/01/FY-2022-Rwanda-MOP.pdf
	Senegal Plan for maternal newborn and family planning 2010 French	2010	https://www.unfpa.org/sites/default/files/admin-resource/Senegal%20Plan%20for%20maternal%20newborn%20and%20family%20planning%202010%20French.pdf
Senegal	PLAN STRATEGIQUE LUTTE CONTRE LES MALADIES NON TRANSMISSIBLES	2017-2020	https://www.sante.gouv.sn/sites/default/files/Plan%20Strat%C3%A9gique_DLMNT.pdf
	Senegal_Nat Strat Plan HIV_2011-2015 fr	2011-2015	https://apf.francophonie.org/IMG/pdf/2013_10_vih_dakar_senegalstrategie.pdf
	Monitoring essential medicine prices, availability and affordability	2008	https://haiweb.org/wp-content/uploads/2015/07/Sao-Tome-and-Principe-Report-Pricing-Surveys.pdf
Sao tome and Princi	WHO country co-operation strategy	2008-13	https://www.who.int/publications/i/item/WHO-CCU-15.03-SaoTomeandPrincipe
	Eliminating malaria in SÃO TOMÉ AND PRÍNCIPE	2015	Document shared
	EXPANDED PROGRAM ON IMMUNIZATION	2016-2020	Document shared
	Malaria Control Strategic Plan	2016 - 2020	https://www.afro.who.int/sites/default/files/2017-05/mcsp.pdf
	national-leprosy-and-tb-strategic-plan-2016-2020-final-draft	2016 - 2020	https://portal.mohs.gov.sl/wp-content/uploads/2021/04/national-leprosy-and-tb-strategic-plan-2016-2020-final-draft.pdf
	Sierra Leone National Strategic Plan on HIV 2016 - 2020	2016 - 2020	https://portal.mohs.gov.sl/wp-content/uploads/2021/04/sierra-leone-hiv-national-strategic-plan-2016-2020.pdf
	Sierra Leone Laboratory System - now & future	2016 - 2020	Document shared
	Sierra Leone & Guinea Lab Diagnostic Training and Transition Plan	2010	https://www.aphl.org/aboutAPHL/publications/Documents/APHL_Sierra_Leone_Guinea_Report_proof6.pdf
Sierra Leone	Lab strengthening in public health emergencies: perspectives from Sierra Leone	2016	http://diadev.eu/images/Lab-Strengthening-Working-Paper_DiaDev_2020_2020-10-07-123308.pdf
	Sierra Leone National Reproductive, Maternal, Newborn, Child & Adolescent Health Strategy	2020	https://www.afro.who.int/publications/sierra-leone-national-reproductive-maternal-newborn-child-and-adolescent-health-policy
	Sierra Leone WHO Profile Diabetes	2017-2021	https://www.who.int/publications/m/item/diabetes-sle-country-profile-sierra-leone-2016
	Integrating Diabetes and HTN into SL Health System	2016	Document shared
	SL Non-communicable Disease		Document shared
	sierra-leone-basic-package	2020	https://mohs2017.files.wordpress.com/2017/06/gosl_2015_basic-package-of-essential-health-services-2015-2020.pdf
	Health Sector Strategic Plan	2010	https://sierraleone.unfpa.org/sites/default/files/pub-pdf/nhssp_2010_15.pdf
	Somalia National Strategic Plan for Malaria	2017-2021	https://moh.gov.so/en/wp-content/uploads/2020/07/Somalia-National-Malaria-Strategic-Plan-2017-2020.pdf
	Strategic Framework for the Somali for HIV	2011-2015	Strategic Framework for the Somali for HIV
Somali	Rep, Maternal, Neonatal, Child, Adolescent Health Strategy	2020-2024	https://www.emro.who.int/somalia/priority-areas/rerproductive-maternal-neonatal-child-and-adolescent-health.html
	TOR - Somalia GF TB Program EQA		Document shared
	depthealthstrategicplanfinal2020-21to2024-25-1	2009-2013	https://www.health.gov.za/wp-content/uploads/2020/11/depthealthstrategicplanfinal2020-21to2024-25-1.pdf
	national-cancer-strategic-framework-2017-2022-min	2020-2024	https://www.health.gov.za/wp-content/uploads/2020/11/national-cancer-strategic-framework-2017-2022-min.pdf
	SouthAfrica_National-Health-Strategic-Plan_2015	2015	https://www.childrenandaids.org/SouthAfrica_National-Health-Strategic-Plan_2015
	South Africa's National Strategic Plan for HIV, TB and STIs_FullDocument_FINAL	2017-2022	https://www.gov.za/sites/default/files/gcis_document/201705/nsp-hiv-tb-stia.pdf
	SouthAfrica_National-Health-Strategic-Plan_2015	2017-2022	https://www.childrenandaids.org/SouthAfrica_National-Health-Strategic-Plan_2015
South Africa	National Health Laboratory Services	2015-2020	Document shared
	Malaria Elimination Strategy Plan for South Africa	2020-2025	https://www.nicd.ac.za/wp-content/uploads/2019/10/MALARIA-ELIMINATION-STRATEGIC-PLAN-FOR-SOUTH-AFRICA-2019-2023-MALARIA-ELIMINATION-STRATEGIC-PLAN-2019-2023.pdf
	South Africa's National Strategic Plan Reduce Maternal & Child Mortality	2019-2023	http://www.kznhealth.gov.za/family/CARMMA_south_africa_strategy.pdf
	South Africa WHO Profile Diabetes		https://www.who.int/publications/m/item/diabetes-zaf-country-profile-south-africa-2016
	Strategic Plan for the Prevention and Control of NCD	2016	https://bhekisisa.org/wp-content/uploads/2022/06/NCDs-NSP-SA-2022-2027-1.pdf
	policy-framework-on-decentralised-management-of-mdr-tb-may-2019_compressed	2013-2017	https://www.health.gov.za/wp-content/uploads/2020/11/policy-framework-on-decentralised-management-of-mdr-tb-may-2019_compressed.pdf
	south_sudan_national_health_policy_2016_to_2025_2	2016	https://extranet.who.int/countryplanningcycles/sites/default/files/planning_cycle_repository/south_sudan/south_sudan_national_health_policy_2016_to_2025_2.pdf
	south-sudan-sbcs-hiv	2016-2026	Document shared
South Sudan	New guidelines for TB Control-South Sudan-V4- Final version 23-06-2016	2019	http://www.arkangelo.org/AAA/AAA%20documents/2016%20documents/New%20guidelines%20for%20TB%20Control-South%20Sudan-V4-%20Final%20version%2023-06-2016.pdf
	South Sudan Reproductive Health Strategic Plan 2013-16	2018-2020	https://healtheducationresources.unesco.org/library/documents/south-sudan-reproductive-health-strategic-plan-2013-16#:~:text=Policy%20Goal%3A%20To%20reduce%20maternal,comprehensive%20reproductive%20health%20care%20package.
	SSMJ Vol 13 No 5 Special Malaria Issue_final	2013-2016	http://www.southsudanmedicaljournal.com/assets/files/Journals/vol_13_iss_5_dec_20/SSMJ%20Vol%2013%20No%205%20Special%20Malaria%20Issue_final.pdf
	hiv_plan_sudan	2020	https://extranet.who.int/countryplanningcycles/sites/default/files/country_docs/Sudan/hiv_plan_sudan.pdf
	sudan_national_health_sector_strategic_plan_nhssp_2012-2016	2004-2009	Document shared
Sudan	sudan_national_health_policy_2007_en	2012-2016	http://sho.gov.sd/controller/dwn_hub_files.php?id=1396
	Sudan-Malaria-Treatment-Protocol_2017	2007	https://reliefweb.int/report/sudan/sudan-malaria-diagnosis-and-treatment-protocol-2017#:~:text=The%20committee%20also%20recommended%20the,of%20intramuscular%20artemether%20in%20Sudan.
	Sudan-National-TB-management-Guideline-March.2019-1	2017	https://www.humanitarianresponse.info/sites/www.humanitarianresponse.info/files/2019/07/Sudan-National-TB-management-Guideline-March.2019-1.pdf
	8.The_Nat_Health_Policy_2017_6th__24_October__2017		https://www.eahealth.org/policy-publications/tanzania-national-health-policy-2017
	national_strategic_plan_for_TB_2015_2020	2017	https://www.tbdiah.org/resources/publications/national-strategic-plan-v-for-tuberculosis-and-leprosy-programme-2015-2020/#:~:text=Abstract%3A%20The%20National%20Strategic%20plan,for%20the%20coming%20five%20years.
	HEALTH SECTOR HIV AND AIDS STRATEGIC PLAN 2017 - 2022	2017-2022	http://library.tacaids.go.tz/bitstream/handle/123456789/128/HEALTH%20SECTOR%20HIV%20AND%20AIDS%20STRATEGIC%20PLAN%202018%20-%202022.pdf?sequence=1&isAllowed=y#:~:text=The%20HSHSP%20is%20a%20sector,Elderly%20and%20Children%20(MOHCDGEC).
Tanzania	TANZANIA_Malaria-Strategic-Plan-2014-2020	2014 - 2020	https://www.out.ac.tz/wp-content/uploads/2019/10/Malaria-Strategic-Plan-2015-2020-1.pdf
	NCD Stategic Plan 2016 - 2020	2016 - 2020	https://extranet.who.int/nutrition/gina/sites/default/filesstore/TZA-2016-2020-NCD%20Strategic%20Plan.pdf
	701320PROP0P110y000Procurement0Plan	2015-2020	https://documents1.worldbank.org/curated/en/209671468009941791/pdf/701320PROP0P110y000Procurement0Plan.pdf
	Health Sector Strategic Plan	2015 - 2020	https://mitu.or.tz/wp-content/uploads/2021/07/Tanzania-Health-Sector-Strategic-Plan-V-17-06-2021-Final-signed.pdf
Togo	TGO-111030	2015-2020	Document shared
	PLAN NATIONAL DE DEVELOPPEMENT SANITAIRE 2017-2022	2021-2025	https://www.afro.who.int/fr/publications/programma-national-de-developpement-sanitaire-du-togo-2017-2022#:~:text=L'%C3%A9laboration%20du%20PNDS%202017,sanitaire%20universelle%20d'autre%20part.
Tunisia	howard-morris_standardization-and-harmonization-in-laboratory-medicine	2017-2022	Document shared
	Test menu Final-Draft_26Oct2017_Signed copy-2	2019	Document shared
	NATIONAL-STRATEGIC-PLAN-FOR-TB-and-LEPROSY_Nov_24_2020_WEB-1	2017-2020	https://www.health.go.ug/cause/national-strategic-plan-for-tuberculosis-and-leprosy-control-2020-21-2024-25/
Uganda	The-Uganda-Malaria-Reduction-Strategic-Plan-2014-2020	2021-2025	http://library.health.go.ug/sites/default/files/resources/The%20Uganda%20Malaria%20Reduction%20Strategic%20Plan%202014-2020.pdf
	Uganda_National-HIV-and-AIDS-Strategic-Plan_2015	2014-2020	https://www.childrenandaids.org/Uganda_National-HIV-and-AIDS-Strategic-Plan_2015#:~:text=The%20National%20HIV%20and%20AIDS%20Strategic%20Plan%202015%2F2016%20%E2%80%932019,and%20discrimination%20due%20to%20HIV%E2%80%9D.
	UGANDA NATIONAL HEALTH LABORATORY SERVICES POLICY	2016-2020	https://www.cphl.go.ug/node/142#:~:text=The%20policy%20sets%20direction%20in,supplies%3B%20human%20resources%3B%20quality%20management
	Zambia National Health Strategic Plan	2017-2021	https://www.moh.gov.zm/?wpfb_dl=182#:~:text=This%20strategic%20plan%20focuses%20on,possible%2C%20Leaving%20No%20One%20Behind.
	UNFPA-RMNCAH-N-Communication-and-Advocacy-Strategy-2018-2021	2017-2021	https://www.unfpa.org/sites/default/files/pub-pdf/UNFPA%20Strategy%20for%20Family%20Planning%202022-2030.pdf
	4 Tuberculosis Manual for Zambia_Final	2018-2021	https://www.afro.who.int/sites/default/files/2019-06/4%20%20Tuberculosis%20Manual%20for%20Zambia_Final.pdf
Zambia	zambia_tuberculosis_programme-stop_tb_strategic_plan_2008-2012	2008-2021	https://dolatiaschan.com/4/5737255
	zmb_str_national_aids_strategic_framework_2017-2021_30may2017	2017-2021	https://www.unicef.org/zambia/media/1031/file/Zambia-national-AIDS-strategic-framework-2017-2021.pdf

	Zambia Strategic Plan NCD Risk Factors	2013-2015	https://www.iccp-portal.org/system/files/plans/ZMB_B3_NCDs%20Strategic%20plan.pdf
	Zambio Biomed Lab Strategic Plan	2013-2016	https://www.moh.gov.zm/?wpfb_dl=82
	National+Malaria+Elimination+Strategic+Plan+2017-Final_PRINT	2017-2022	https://static1.squarespace.com/static/58d002f017bffcf99fe21889/t/5b28d7f1575d1ff0942dbce1/1529403401067/National+Malaria+Elimination+Strategic+Plan+2017-Final_PRINT.pdf
Zimbabwe	Harmonization and Standardisation Document_Zimbabwe 2015	2015	Document shared
	Zimbabwe Health Sector HIV and STI Strategy	2021-2025	http://www.mohcc.gov.zw/index.php?option=com_phocadownload&view=category&download=672:comprehensive-national-strategy-final-digital-pdf&id=10:reports&Itemid=720
	National Health Laboratory Strategic Plan	2017-2021	https://www.unicef.org/zimbabwe/media/411/file/National%20Health%20Laboratory%20Strategic%20Plan.pdf
	The National Malaria Control Programme Strategy	2008-2013	https://d1u4sg1s9ptc4z.cloudfront.net/uploads/2022/01/FY-2022-Zimbabwe-MOP.pdf
	Zimbabwe National Maternal and Neonatal Health Road Map 2007-2015	2007-2015	https://extranet.who.int/countryplanningcycles/sites/default/files/planning_cycle_repository/zimbabwe/zimbabwe_mnh_roadmap_2007-2015_0.pdf
	Final2 draft NHL Strategic Plan 2022-2026	2022-2026	https://www.northmankato.com/sites/default/files/documents/2021%20Strategic%20Plan%20Attachments_0.pdf
	National Tuberculosis Program Strategic Plan	2017-2020	https://depts.washington.edu/edgh/zw/hit/web/project-resources/TB-NSP.pdf
