# Supplementary material for: Contexts for developing of national essential diagnostics list. Lessons from a mixed-methods study of existing documents, stakeholders and decision making on tier-specific essential in-vitro diagnostics in African countries
Source: PLOS Glob Public Health. 2023 May 18;3(5):e0001893. doi: 10.1371/journal.pgph.0001893 (PMC10194858; doi:10.1371/journal.pgph.0001893)
Supplement: S3 Table — (PDF) [file pgph.0001893.s003.pdf]

Name of country	Name of Document	URL
eSwatini	Eswatini HLS Strategic Plan 2021-2023-Cleaned-16Feb2022_Final Signed	https://www.ers.org.sz/documents/1616518535.pdf
Ghana	GHANA LAB POLICY final (310513) Accepted - June 23, 2015	https://www.afro.who.int/sites/default/files/2017-05/annual-report-2015--wco-ghana-.pdf
Ghana	GHANA NHLSP Draft (2) 14-3-12 -final draft	Document shared
Kenya	Kenya_SP NPHL March_Final_March 27th 2017	Document shared
Malawi	Malawi_National Laboratory Policy Approved Version 13-03-2018.	Document shared
Nigeria	Nigeria _ Approved Nigeria National Medical Laboratory Services Policy_FP	Document shared
Nigeria	Nigeria _ Reviewed version of NMLStP from group of lab experts November 2021 X	Document shared
Sierra Leone	Sierra Leone _ final NMLSTP	Document shared
Tanzania	Tanzania_NHLSP II_Final_6-5-2016	Document shared
Uganda	UGANDA NATIONAL HEALTH LABORATORY SERVICES POLICY II 2016	https://www.cphl.go.ug/sites/default/files/2019-06/UG%20NHLS%20Policy%20%20LTC%20Final%20draft%20-%20Thomas-Pizaro-Gaspard%20signed23Mar2018.pdf
Cameroon	Cameroon - Plan Strategic National du Develpmt labo 2018-2020_Eng	http://onsp.minsante.cm/en/publication/307/cameroon-national-laboratory-policy
Niger	PLAN_STRATEGIQUE_RECERCHE_EN_SANTE_2013_2020adoptjuin	https://healthresearchwebafrica.org.za/files/PLAN_STRATEGIQUE_RECERCHE_EN_SANTE_2013_2020adoptjuin..pdf
Niger	PNS_VERSION FINALE NIGER	https://www.dphmt-msp.ne/sites/default/files/textes/PNS_VERSION%20FINALE%20NIGER.pdf
Niger	PSA Niger 2019-2023 VF	Document shared
Madagascar	MDG_B3_B DOC PSNLIMNT- PH ce 02072018_	Document shared
Madagascar	Plan Stratégique de Renforcement du Système d'Information Sanitaire	https://www.measureevaluation.org/resources/publications/sr-17-146/at_download/document#:~:text=Le%20Plan%20de%20D%C3%A9veloppement%20du,interventions%20futures%20de%20d%C3%A9veloppement%20sanitaire.
Madagascar	Plan strategique national de renforcement de la sante communautaire	http://www.healthpolicyplus.com/ns/pubs/17365-17659_PSNRSC.pdf
Madagascar	Plan-de-développement-du-secteur-santé-2020-2024. Eng	https://scorecard.prb.org/wp-content/uploads/2022/03/Plan-de-de%CC%81veloppement-du-secteur-sante%CC%81-2020-2024..pdf
Madagascar	Plan-Stratégique-SISAL-2016-2020_Eng	http://www.plateforme-elsa.org/wp-content/uploads/2017/03/Plan-Strate%CC%81gique-SISAL-2016-2020.pdf
Cape Verde	IV Plano Estrategico Nacional VIH-SIDA2020	https://ccssida.cv/wp-content/uploads/2021/10/IV-Plano-Estrategico-Nacional-VIH-SIDA2020.cleaned.pdf
Cape Verde	PEDRHS Ministerio Saude 2016	Document shared
Cape Verde	PNDS 2017-2021 -VOL I Definitivo	Document shared
Cape Verde	PNDS 2017-2021 -VOL I Definitivo_Eng	Document shared
Guinea-Bissau	PLAN STRATEGIQUE CHOLERA GUINEE BISSAU_VF	https://www.plateformecholera.info/index.php/bonus-page-2/national-strategies-plans/guinea-bissau/152-wca/strategic-framework/library-of-national-plans/445-plan-strategique-de-prevention-et-de-riposte-contre-le-cholera-en-guinee-bissau
Guinea-Bissau	Plano Nacional do Desenvolvimento Sanitário	https://extranet.who.int/mindbank/item/3460
Guinea-Bissau	plano_nacional_de_desenvolvimento_sanit__rio_1997-2001	https://extranet.who.int/mindbank/download_file/3649/7fb7eeef5d1f630b2968024b372fd64313680f74
Sao Tome and Principe	PNS1_validacao	Document shared
Sychelles	National-Health-Strategic-Plan-2022-2026-Full-Version	http://www.health.gov.sc/wp-content/uploads/National-Health-Strategic-Plan-2022-2026-Full-Version.pdf
Equatorial Guinea	National health development plan	https://www.guineasalud.org/archivos/Protocolos/Plan.pdf
Democratic Republic of the Cong	Politique Nationale sur l'Achat Strategique des	Document shared
Democratic Republic of the Cong	RDC-Plan-National-de-Developpement-Sanitaire-2016-2020	https://www.prb.org/wp-content/uploads/2020/06/RDC-Plan-National-de-Developpement-Sanitaire-2016-2020.pdf
Benin	VF_PNS__19_01_2020_VF Politique Nationale De Sante 2018-2030	https://files.aho.afro.who.int/afahobckpcontainer/production/files/VF_PNS__19_01_2020_VF.pdf
Rwanda	PNADF435 National Medical Laboratory Policy	https://pdf.usaid.gov/pdf_docs/PNADF435.pdf
Ethiopia	Ethiopia Lab Master Plan_2nd Edition	https://www.ephi.gov.et/images/downloads/Ethiopia%20Lab%20Master%20Plan_2nd%20Edition.pdf
