# Supplementary material for: Contexts for developing of national essential diagnostics list. Lessons from a mixed-methods study of existing documents, stakeholders and decision making on tier-specific essential in-vitro diagnostics in African countries
Source: PLOS Glob Public Health. 2023 May 18;3(5):e0001893. doi: 10.1371/journal.pgph.0001893 (PMC10194858; doi:10.1371/journal.pgph.0001893)
Supplement: S4 Table — (PDF) [file pgph.0001893.s004.pdf]

number if tests taken up in country strategy	year of WHO approval
25	1999
24	1999
15	1999
16	1999
24	1999
20	1999
22	2009
12	2009
16	2009
2	2017
23	2008
22	2008
13	2008
3	2008
12	2008
12	2008
12	2008
3	2015
2	2015
22	2006
25	2006
12	2006
9	2013
7	2013
2	2017
13	2003
12	2003
20	2013
12	2013
26	1989
