# Supplementary material for: Contexts for developing of national essential diagnostics list. Lessons from a mixed-methods study of existing documents, stakeholders and decision making on tier-specific essential in-vitro diagnostics in African countries
Source: PLOS Glob Public Health. 2023 May 18;3(5):e0001893. doi: 10.1371/journal.pgph.0001893 (PMC10194858; doi:10.1371/journal.pgph.0001893)
Supplement: S2 Data — (PDF) [file pgph.0001893.s006.pdf]

SUMMARY OF QUALITATIVE DATA BY COUNTRY

(anonymized participants)

INTERVIEW PARTICIPANTS FOR COUNTRY: BURKINA FASO

# INT	Place of work participants
1	Laboratory Services MoH
1	Laboratory Services MoH
2	Hospital Laboratory

DOCUMENTS THAT ARE ANALYSED

# DOC	Name document, year
**1**	Direction des laboratoires, juillet 2009 : Normes en infrastructures, équipements et analyses de biologie médicale essentielles des formations sanitaires publiques

FINDINGS BY THEME

Global themes	Specific themes	Findings/Answers (*Refer to # INT and # DOC*)
**MoH organisation**	Position laboratories in MoH *(separate department or sub-division?)*	**In terms of hierarchy, the Medical Laboratories Directorate is divided into three departments and is a sub-component of the Directorate for Access to Health Products. The latter is affiliated with an agency of the Ministry of Health called the National Agency for Pharmaceutical Regulation.**<br>#1 : National Agency for Pharmaceutical Regulation<br>- Directorate for Access to Health Products<br>o Directorate for Medical Laboratories<br>The Directorate for Medical Biology Laboratories includes:<br>- In vitro medical devices department<br>- Medical biology laboratory monitoring department<br>- Evaluation and quality management follow-up service
**Laboratory system**	Tiers in laboratory system	???
	Laboratory tiers related to tiers health system (*including community level*)	**The laboratories follow the health system of Burkina Faso and are present in four tiers the:**<br>- The Health and Social Promotion Center (CSPS),<br>- The Medical Center/Medical Center with Surgical Branch (CM/CMA),<br>- The Regional Hospital Center (CHR)<br>- The National or University Hospital Center (CHN/CHU) (DOC1)
	Distribution of laboratories across public, private for profit / not for profit	**There are about 155 public laboratories and about 50 private laboratories in Burkina Faso.**<br>150 to 160 public laboratories ; 54 private laboratories approximately (#1)
	Challenges in laboratory system, differentiating for tiers and areas *(problems in availability and access to – specific - IVDs)*	**In Burkina Faso, several challenges concerning the technical set-up (inputs and equipment), human resources and the budget allocated to the laboratories have been identified**.<br>#1: 1) Shortage of inputs despite quantification of needs in advance; 2) Problem of Equipment maintenance ; 3) Problem of financing the action plan for the laboratories<br>#2: Insufficient human resources in the city while there are many in the periphery
**Stakeholders in lab system and services**	Technical	#1: OMS, CDC
	Implementing	
	Funding	#1: ASLM

	MoH departments / states / regions	
**National committee/working groups on IVDs**	Committee name(s) and aims	**Two different and complementary committees (quality and biosafety) have been set up in Burkina Faso.**<br><br>DOC1: 1) The committee of experts on standards, techniques, methods, protocols and algorithms for biological diagnosis; 2) The committee of experts on infrastructure, biosafety, equipment, personnel, reagents and laboratory consumables.
	Categories of committee members, % women	**Each committee includes several specialists from different fields related to the specific aspects of the laboratories. The distribution between men and women is not provided for Burkina Faso.**<br><br>Doc 1: The expert committee on standards, techniques, methods, protocols and algorithms for biological diagnostics is composed as follows<br><br>- a specialist in clinical biochemistry and an alternate<br>- a specialist in bacteriology-virology and an alternate<br>- a parasitology-mycology specialist and an alternate<br>- a specialist in biological hematology and an alternate<br>- a specialist in immunology and an alternate<br>- a specialist in anatomy cytology pathology and an alternate<br>- a specialist in the organization of laboratory networks and an alternate<br>- a specialist in medical biology from the laboratory directorate and an alternate<br>- a veterinary biologist and an alternate<br>- a specialist in public health and an alternate<br>- a specialist in quality management and an alternate<br>- an infectious disease specialist and an alternate<br>- a specialist in molecular biology and an alternate<br>- a specialist in blood transfusion and an alternate<br>- two medical biologists from private medical laboratories and two alternates<br><br>The committee of experts on infrastructure, biosafety/biosecurity, equipment, personnel, reagents and laboratory consumables is composed as follows<br><br>- a biomedical engineer or senior biomedical technician<br>- a specialist in public procurement<br>- a specialist in the organization of laboratory networks and reference laboratories and a deputy<br>- a specialist in the management of supplies, equipment and laboratory reagents and a substitute<br>- a specialist in the management of priority programs for malaria, HIV/AIDS and tuberculosis<br>- a veterinary biologist with experience in procurement of laboratory equipment and reagents<br>- an architect with expertise in medical laboratory design and an alternate<br>- a biosafety/biosecurity specialist and an alternate<br>- a resource person from CAMEG<br>- a resource person from the Ministry of Health's Administration and Finance Department (DAF)<br>- a resource person from the Health Development Support Program (PADS)<br>- a resource person from the Ministry of Health's Public Procurement Department (DMP)<br>- a resource person from the Health Products Access Branch (HPAB)<br>- a resource person from the biomedical equipment and maintenance management company (SOGEMAB)

	Who decides on the members of these committees and what are criteria for membership	**There is no one person who decides who can be a member of the committees. In the event of a meeting, the directorate of laboratories sends a letter to the stakeholders who, at their level, designate someone to represent them.**<br><br>#2: Each laboratory has quality correspondents and biosafety correspondents<br><br>The management of the laboratories sends a correspondence to ask for the sending of an agent who will represent the hospital and the laboratory at the level of these authorities.
	Missing stakeholders in committees?	**Associations working in the field of laboratories were perceived as absent.**<br><br>#2: The association: people think that the elaboration of documents is technical and do not see the usefulness of involving an association
	If no committee: what the MoH plans/ what are barriers ?	
**Presence of national laboratory documents**	Laboratory policy (*Look at date*)	Guide de bonne exécution des analyses de biologie médicale au Burkina Faso, 2009<br><br>This guide to the proper performance of medical analysis defines the requirements that medical laboratories must comply with, regardless of their status. It will serve as a tool for inspection and supervision
	Strategic plan<br><br>*(Look at date, budget?)*	
**Presence of documents with guidelines on IVDs**	National documents that address (essential) IVDs (*dates, budget?)*	Normes en infrastructures, équipements et analyses de biologie médicale essentielles des formations sanitaires publiques, 2009<br><br>No budget reported
	What vertical (disease) programs identify priority IVDs ?	
	Whether documents define (essential) IVDs by tier – if so, which tiers? (including community level)	**The document defines the essential tests at each level of the laboratory system including the community level where some rapid tests are performed by nurses, i.e. malaria, urine tests, HIV,.**<br><br>#1: At the community level, programmes define tests: malaria, urine tests, HIV, some small tests; these are performed by the health workers present, mainly the nurses.
	If no/not all documents address IVDs by tier: What are the reasons?	
	If no/not all documents address community tier: What are the reasons?	
**Stakeholders, processes and criteria for prioritizing / selecting IVDs**	Category of stakeholders involved in development of documents addressing IVDs	**First, there are the participants in the workshop to amend the laboratory standards who are directly involved in the organization, management or acts of laboratories (prescribers and technicians). Secondly, the validation workshop was open to all national stakeholders.**<br><br>Doc 1: 1) The expert committee on standards, techniques, methods, protocols and algorithms for biological diagnostics; 2) The expert committee on infrastructure, biosafety/biosecurity, equipment, personnel, reagents and laboratory consumables<br><br>Participating in the workshop to amend laboratory standards were: Ordre des médecins, Direction des Laboratoires, CHU PCDG, Ordre des pharmaciens, LNSP, CHR Ouahigouya, DGPML, Centre Muraz, CHR Koudougou, DGIEM/DES, DGIEM/DIS, PNT,

		DSP, CHU YO, CMA Pissy, ITSS, CMA secteur 30, CMA Boromo, CHR Tenkodogo, DHP, DPM<br><br>Participating in the validation workshop of the laboratory standards: CNRFP, DHP, CHU PCDG, DGPML/DRP, CNRFP, DGPML, Ordre des médecins, Direction des Laboratoires, CNTS, CHU PCDG, Pharmacie de l'avenir, DEP/Santé, LNSP, CHR Ouahigouya, CHUSS, CHR Koudougou, Ordre National des vétérinaires, Clinique SANDOF, DGIEM/DES, DGIEM/DIS, Ordre National des pharmaciens, Service Santé des armées, DSP, DRS Hauts Bassins, DLM, PNT, DRS Est, LNSP, DRS Centre, DGPML/DMPT, Ordre National des infirmiers/ères
	Steps in development of documents (*consultants, drafts, workshops?)*	**The document was developed in two phases: the first phase consisted in organizing a workshop to amend the draft developed by the laboratory directorate. Then, in the second phase, a validation workshop was organized with all stakeholders. A revision phase of the document was planned but, due to a lack of financial means, this was not done.**<br><br>Doc 1: organization of two workshops: 1) Workshop to amend the laboratory standards; 2) Workshop to validate the laboratory standards<br><br>#1: The initial document has undergone some modifications: In relation to this prioritization of essential examinations, these examinations that appear on the essential lists were not defined just like that: we had to invite all of the laboratory actors by level of care, of course, and on these occasions they were asked to say exactly which examinations they would like to make available at their level. But the changes only concern the input availability form. The revision was caused by the rapid evolution of the epidemiological situation and on the other hand by the capacity of the practitioners to perform a certain number of examinations. Indeed, as the training curricula for medical biologists evolve, this must also be translated into the field in terms of an increase in the minimum package of tests to be offered to the population. Periodically, the field actors meet to discuss the updating of the list. The field actors are asked "Do you think that this list is still up to date? Are there examinations that should be pruned in favor of others or should they be maintained? It is the field actors who guide these revisions. The actors are consulted during the training and promotion activities organized by the laboratory management. If the ideas converge towards a complete revision, the laboratory management considers how to organize a workshop for the revision. The revision of the minimum package often faces the financial obstacle of organizing it.
	Criteria for selecting essential IVDs (by tier, clinical care or disease surveillance)	**In Burkina Faso, the choice of essential tests at each level was mainly based on the type of personnel present in the health services. The tests were defined according to the capacities of the health personnel present. Epidemiological criteria and accessibility were also important criteria.**<br><br>#1: 1) Based on the staff present at each level and their skills; 2) Epidemiological criteria based on the principle that certain diseases are endemic in some areas but not in others; 3) The ease of transporting inputs to enable these examinations to be carried out; "For example, at the level of the CM/CMA, there are certain examinations that were considered feasible at the patient's bed, such as urine tests. We removed them from the list, thinking that midwives could perform them at the patient's bed, so this test was simply removed from the list and replaced by the HBS antigen.<br><br>When we go up to the RHC level, the same criteria were used to define the different examinations that must be available; this is how we were able to decide on 13 for the CM/CMA and 23 for the RHC. Thus, as of today, we are at 13 for the CM/CMA and 23 for the CHR. As for the UHCs, we have not been able to define a list, knowing that at the UHC level all the examinations should be feasible.<br><br>#2: The prioritization criteria are, in my opinion, a function of the technical platforms and the skills assigned to these locations.

	Discussions and (dis)agreements in prioritizing IVDs – by tier (*note: RDTs in community?)*	
**Intention and plans to develop NEDL *(processes in development of Nigeria NEDL)***	Knowledge about WHO EDL and perceived usefulness of WHO guidelines for developing NEDL?	**Some staff of the Laboratory Services have already participated in an international workshop where the EDL was presented and believe that this list is not very different from the one used in Burkina Faso.**<br><br>#1: Lack of knowledge of WHO EDL. "Personally, I have already heard of it, I even had the 2018 version. We think it is quite good and ambitious; when we look at the examinations, they are defined by pathology; it is the same situation we have here: for example, we see the examinations that must be available for malaria, HIV and so on."<br><br>Thinks that it is structured in the same way as in Burkina Faso: "It is practically the same structure except that for the WHO we put the examinations opposite the pathologies, that's it: when we confronted the new list with that of 2018 it was practically the same framework."<br><br>« Je sais qu'en 2018 on était au forum mondial sur les dispositifs médicaux où cette liste nous avait été présentée et il avait été demandé à chaque pays de donner son expérience. Je ne vais pas donner les noms des pays (rires) mais c'est le Burkina qui avait quand même une liste de diagnostics essentiels en respectant sa pyramide sanitaire, parce que la plupart des pays de l'Afrique de l'ouest étaient là, mais nous on a pu présenter quelque chose qui s'apparentait un peu à la liste de l'OMS »
	MOH's taken and/or planned steps for developing NEDL or similar document (who took the initiative, survey, who writes, stakeholders involved, validation etc)	**The development of an essential diagnostic list for Burkina Faso is not yet on the agenda. They already have financial difficulties in revising their document.**<br><br>#1: There is not yet a plan to develop an NEDL. Laboratory management has housed this list of essential diagnostics in the essential drug list. In fact, there is a national list of essential medicines and within it there are several headings: there is a heading for essential consumables, essential reagents, essential medicines, essential diagnostics.<br><br>Q: So if it were to be updated, the global list of everything else would have to be updated as well? Answer: Normally, the revision of the list of essential medicines should be done for both lists and managed by the National Agency for Pharmaceutical Regulation. The management of the laboratories is always invited to the review and if there is any input to be made, it is usually agreed with the laboratory actors when the regulations proceed to the review.
	Perception of usefulness of NEDL in solving (some) barriers to availability of IVDs in tiers / lab system	**The WHO EDL improves what already exists in Burkina Faso and allows for internationally harmonized data with all countries using the same list.**<br><br>#1: "I think it is a serious tool to be implemented everywhere; if it is the same one that all countries use, I think it will facilitate the cross-referencing of data so that at a given time, we can know what is the availability rate, the breakage rate for a given input at the national level. It improves what already exists."
	Present and foreseen challenges in development of NEDL	
**Implementation of NEDL**	Plans and steps for implementation	
	Which stakeholders are /will be involved in implementation	
	What problems in lab services will/may be solved	

	with successful implementation of NEDL?	
	(Foreseen/possible) Problems in implementation of NEDL and how to solve these problems	
**Recommendations**	For own country: steps to take in developing and implementing NEDL by tier	**A small group must be identified to work on a first draft which will then be submitted to a larger group for validation. The document must also be very practical and not theoretical, otherwise it will be useless**<br>#1: First, organize a workshop by level of care to draft the list. Two workshops should be organized: a drafting workshop, then a validation workshop before the conclusion.<br>In order for the laboratory management to organize these workshops, it needs the support of partners and the Ministry of Health.<br>#2: I think that at the very beginning, we can designate a core group of resource persons who will work continuously for 2 to 3 consecutive weeks, release these activities according to the level and then hold a workshop extended to other components, why not even civil society, to bring in their contribution to what has already been done by this core group.<br>In this core group, we need the heads of the laboratories at the national level, at the peripheral level, we need the prescribers at the national level, at the peripheral level, who will all sit down according to their daily lives and their functions and then according to what is available in the field, even if it is necessary to bring something more, to set up the framework of something that can be improved by the large group.<br>If we start doing folk workshops people are going to go out there and sit around and just talk, there will be nothing.<br>And there is no point in writing a document that is difficult to apply because very often we are too theoretical; we must be pragmatic, practical on the ground.<br>So this document, which will be written by the core group, will be submitted to the large group, perhaps even in advance, which will read it and add to it, and then organize a 5-day workshop to validate it.
	For other countries: steps to take in developing NEDL by tier *(Nigeria's lessons)*	
	For development of NEDL: considering gender of recipients	**Prioritization of tests by gender is not relevant because there are no female-specific problems other than pregnancy and women can get pregnant anywhere.**<br>#1: Prioritizing by gender is not too relevant because there are the same conditions for everyone except for pregnancy in women.<br>#2: No, I think that this case...it's true that we very often put women and children in front...it's good but the man must also be healthy. In any case, if there are resources, I think it is not even necessary to make this distinction: we put in place something that will cover both children and women and men.
	For ASLM / FIND/ WHO: type of support for countries in development and implementation of NEDL	Not asked

INTERVIEW PARTICIPANTS FOR COUNTRY: CAMEROON

# INT	Place of work participants
1	Hospital Laboratory
1	National Public Health Laboratory
1	Partner
2	Laboratory Services, MoH
3	Partner
3	Partner
3	Partner

DOCUMENTS THAT ARE ANALYSED

# DOC	Name document, year
1	Plan stratégique national de développement des laboratoires du Cameroun
2	Organisation des laboratoires suivant la pyramide sanitaire au Cameroun
3	Plan stratégique national de lutte contre le paludisme

FINDINGS BY THEME

<table>
<tr><th>Global themes</th><th>Specific themes</th><th>Findings/Answers (Refer to # INT and # DOC)</th></tr>
<tr><td>MoH organisation</td><td>Position laboratories in MoH (separate department or sub-division?)</td><td>#1 : Ministère de la santé -> Inspection générale des services pharmaceutiques et des laboratoires -> Direction nationale des laboratoires, des médicaments et de la pharmacie -> Laboratoire national de santé publique
Le laboratoire national de santé publique n’a pas encore une structure juridique. Pour son fonctionnement interne, laboratoire national de santé publique est organisé en sections
• Qualité
• Biologie moléculaire
• Résistance aux antimicrobiens
• Surveillance épidémiologique
• Surveillance génomique
• Logistique
• Sérologie et hémato biochimie
• Gestion de données
• Anapathe
• Phytopathologie</td></tr>
<tr><td rowspan="3">Laboratory system</td><td>Tiers in laboratory system</td><td></td></tr>
<tr><td>Laboratory tiers related to tiers health system (including community level)</td><td>#1 : Dans le secteur public, les laboratoires sont organisés en quatre niveaux ; ces niveaux sont différents des 3 niveaux de la pyramide sanitaire nationale :
- Le Niveau I : CSI et CMA
- Le Niveau II : HD
- Le Niveau III : HR
- Le Niveau IV : HC et HG</td></tr>
<tr><td>Distribution of laboratories across public, private for profit / not for profit</td><td>#1 :
Répartition :
- Laboratoires publics occupent pratiquement 90%
- Les laboratoires de secteur confessionnels, privés, laïcs, 10%.
On distingue le privé confessionnel et le privé laïc.
Des laboratoires privés qui sont de deux types :
- Les laboratoires privés faisant partie des formations sanitaires privées qui sont comparables à ceux du secteur public ;
- Les laboratoires d'analyses médicales privés indépendants
#2 : Pour les laboratoires privés à but non lucratif : on en a moins d’une vingtaine actuellement</td></tr>
</table>

		- Ce ne sont pas forcément des laboratoires mais des formations sanitaires qui ont en leur sein un laboratoire avec des services gratuits pour un type de patients encore que ce n'est pas pour tout le monde : malgré que ce soit à but non lucratif, ils font une différence entre indigents et non indigents.<br>- Il y a d'autres laboratoires gratuits pour tous les patients qui sont enregistrés dans leur système, quelle que soit l'analyse, le type de soins tant que c'est sur place ; dès que c'est à l'extérieur c'est aux frais du patient.<br>- Ils représentent 5% par rapport aux laboratoires au niveau pays<br>- Ils vont vraiment se concentrer dans ces zones où il n'y a pas un accès facile à nos hôpitaux conventionnels, pas assez de routes, où les populations sont vraiment démunies. Pour certaines pathologies il y a quand même un taux forfaitaire qui est demandé mais qui ne représente rien par rapport à ce que ce serait si vous étiez en milieu hospitalier public.<br><br>Les laboratoires à but lucratifs représentent moins de 20%<br><br>- Il y en a qui sont lucratifs et confessionnels<br>- Ils ratissent une forte population parce que la fréquentation est dense<br>- Ils sont installés en a en périphérie, en zone périurbaine, en zone urbaine-urbaine, et aussi en zone communautaire<br><br>Les laboratoires publics représentent plus de 70%
	Challenges in laboratory system, differentiating for tiers and areas *(problems in availability and access to – specific - IVDs)*	#1 : Approvisionnement erratique : Sauf pour les cadres de TB, les tests relatifs à la prise en charge du VIH comme les tests de dépistage rapide, la charge virale, le diagnostic précoce et dans le cadre de la Covid, les réactifs sont achetés au niveau national et distribués sur sites, sélectionnés. Dans le cadre des autres analyses et diagnostics, chaque laboratoire a la responsabilité de la procurer ses réactifs<br><br>DOC 1 : Problèmes liés aux ressources humaines disponibles : 1) Répartition inadéquate des personnels qualifié ; 2) Disparité des diplômes et des titres des professionnels de la biologie médicale ; 3) Absence d'harmonisation et de validation des curricula de formation ; 4) Absence d'un système de formation continue et de recyclage du personnel.<br><br>#1 : Budget insuffisant : 1) Il n'y a pas de ligne budgétaire pour les laboratoires, nous dépendons de financements de partenaires ; 2) Les services de laboratoire sont noyés dans la direction de la pharmacie et du médicament et le laboratoire vient en dernier lieu et malheureusement tous les directeurs qui ont occupé ce poste de directeur de la DPLM ne sont jamais venus du laboratoire.<br><br>#1 : Problème de l'homologation : 1) il y a tant de réactifs qu'on ne sait même pas par quelle porte ils entrent dans le territoire ; 2) Parfois les fournisseurs obtiennent l'autorisation de mise sur le marché alors même que le réactif n'a pas été homologués mais puisqu'ils ont cette autorisation, c'est facile pour eux d'acheter.<br><br>Doc 3 : « La Direction de la Pharmacie et du Laboratoire, l'Inspection Générale de la Pharmacie et du Laboratoire, le Laboratoire National de Contrôle Qualité des Médicaments (LANACOME) sont des structures responsables de la règlementation, du suivi des approvisionnements, du contrôle des structures sanitaires et de l'Assurance Qualité des médicaments et consommables médicaux »<br><br>#1 : Problème de la mercuriale ; 1) Il y a le fait que les réactifs de laboratoire n'ont pas encore fait la mercuriale qui est une liste de produits à acheter avec les prix ; 2) Cela permet que, si vous êtes une structure publique et que vous voulez acheter quelque chose et qu'il faut passer par une passation de marché, vous devez obligatoirement utiliser la mercuriale qui fixe les prix.<br><br>- *La mercuriale, pour vous dire que la mercuriale est utilisée en ce qui concerne la commande des réactifs dans notre structure. Le problème est que dans la*

		- *mercuriale ce ne sont pas les noms des fabricants qui sont mis, mais on met la dénomination du réactif tel qu'on l'utilise.*<br>- *Donc la mercuriale en ce qui concerne les laboratoires, ce n'est pas le nom du fabricant mais le nom du produit, le nom de base.*<br><br>#2 : Le problème qui est la maîtrise de la qualité des réactifs qui entrent dans le pays<br><br>- Il y a une pléthore de réactifs dont on n'est pas toujours sûrs de leur qualité pour pouvoir garantir des résultats de qualité aussi.<br>- Il n'y a pas une structure qui s'occupe particulièrement de l'homologation mais le contrôle est limité car il y a beaucoup de porosité au niveau des frontières<br><br>La maîtrise de la chaîne de froid<br><br>#3: Absence of ownership and leadership at the central level.<br><br>- As I said CDC has accomplished all of this through implementing partners and support from other stakeholders.<br>- But the lab is only present at the central level.<br><br>In DPLM I think you have 5 staff who support lab activities and there is no lab presence in the regions<br><br>- So in the regions it's only our IP (implementing Partners) who conduct lab activities across the regions.<br><br>There is no finance, there is no funding for lab practice, it's very limited.
**Stakeholders in lab system and services**	Technical	#1: OMS et UNICEF, ASLM, FIND, Africasud<br><br>#2 : CDC, OMS et ASLM, Global Health System Control-Procurement on Supply Management<br><br>#3 : CDC is actually the brain of the entire lab practice in Cameroon, because before CDC, Cameroon didn't have a Department for Labs, so what they had was DPM, pharmacy and medicine, lab came in in 2013.<br><br>CDC started working in 2009 with labs. 2009 was the launching of quality assurance activities for labs but in Cameroon they've started working with labs since 2001. But at that time there was no department there was no department dedicated for labs<br><br>The National Lab Strategic Plan was drafted was supported by CDC, the Lab network mapping was done, the National health public labs created through the support of CDC
	Implementing	#2: Global Hair Solution System, Métabiota, CHAI, IDDS, CHAI, CDFI, Georgetown University, ICAP, Elisabeth Glizer Foundation), UNICEF
	Funding	#1 : Fonds mondial, Banque mondiale
	MoH departments / states / regions	#1 : Métabiota, IDDS qui nous aide dans le transport des échantillons
**National committee/working groups on IVDs**	Committee name(s) and aims	#1 : Il n'y a pas de comité<br><br>Pour l'élaboration des documents, il n'y a que la DPLM comme structure élaborant les lignes directrices et les politiques en collaboration avec certaines directions techniques du ministère.<br><br>Il n'y a pas encore un groupe technique mais c'est en projet. Je pense que lorsqu'il y aura la politique nationale des laboratoires, on pourra mettre en place les différents groupes techniques

	Categories of committee members, % women	
	Who decides on the members of these committees and what are criteria for membership	
	Missing stakeholders in committees?	
	If no committee: what the MoH plans/ what are barriers ?	#2 : Cela a été proposé mais il a du mal à passer au niveau de l'organigramme.<br><br>On espère qu'avec le temps ça va passer mais en général dans notre cahier de charges on pense à tout ce qui est réglementaire, normatif et autre, mais jamais on ne travaille tous seuls sur les drafts. On fait appel à notre direction des affaires juridiques et des contentieux très souvent, et aussi si c'est la règlementation est accentuée sur les maladies de programmes comme le VIH, le Covid, le paludisme et autres, On fait appel à ces programmes-là et ensemble on s'assoit, on prend des orientations en fonction de tout ce qui est stratégie nationale et on rédige le document.<br><br>Il y a des drafts dans le tiroir comme on dit, mais il y a des prérequis que nous n'avons pas<br><br>Le principal obstacle c'est le financement du fonctionnement de ce comité technique de laboratoire, ce n'était pas pensé dans la planification et tout.<br><br>Dès que vous utilisez le mot comité il y a un financement pour gérer les membres du comité car il y a un cahier de charges dès que vous le mettez en place forcément, il y a des impacts financiers ; ce n'est pas budgétisé et le ministère de la santé n'est pas prêt à prendre sur lui ce budget.
**Presence of national laboratory documents**	Laboratory policy (*Look at date*)	Organisation des laboratoires selon la pyramide sanitaire au Cameroun, 2011
	Strategic plan<br><br>*(Look at date, budget?)*	Plan stratégique national de développement des laboratoires du Cameroun, (2018-2022 ; cout : 95 997 312 $)
**Presence of documents with guidelines on IVDs**	National documents that address (essential) IVDs (*dates, budget?)*	Organisation des laboratoires selon la pyramide sanitaire au Cameroun, 2011
	What vertical (disease) programs identify priority IVDs ?	
	Whether documents define (essential) IVDs by tier – if so, which tiers? (including community level)	#2 : C'est dans ce document qu'on va définir le paquet de services minimum en fonction du niveau de laboratoire et voir le paquet minimum d'un niveau supérieur peut être le paquet complémentaire d'un autre niveau. Imaginons que vous êtes CSI et que vous êtes dans une contrée où il y a de fortes populations, un appui des partenaires qui sont capables de vous fournir un certain niveau d'équipement, vous pouvez en plus de votre paquet minimum avoir d'autres analyses que nous avons mises sous le nom de paquet de services complémentaires. Elles doivent être des analyses faites par une formation sanitaire de niveau supérieur à vous mais à chaque fois on limite le niveau jusqu'où vous pouvez aller ; c'est donc dans ce document qu'on renseigne les analyses qui peuvent être faites sur le minimum de compétences techniques qu'il faut dans ces formations sanitaires-là.<br><br>#3: That's really a very good document but unfortunately it is not implemented. If I say so because if you follow that doc, it was done according to the different levels.

		La validation est en cours. Les aspects périphériques sont prêts. Il reste à relire puis demander l'avis des experts dans les différents domaines et dans les laboratoires pour pouvoir apprécier ce qui a été fait, et ensuite faire un atelier de validation.<br><br>Il y a d'abord un atelier de lecture et d'amendement du document qu'il va falloir faire, ensuite un atelier de validation du document et enfin un atelier d'adoption. Et il faudra bien imprimer le document pour pouvoir le mettre à disposition des utilisateurs. À cette phase c'est vraiment la DPML qui s'en charge.<br><br>#1 : Au niveau communautaire, il y a ce qu'on appelle les agents de relais communautaires qu'on forme pour faire des analyses au niveau communautaires, certains tests rapides.<br><br>- VIH et le test covid<br>- Paludisme<br><br>#2 : il y a le test de grossesse qui est gratuit pour les PV VIH. Pour les autres c'est disponible mais payant.
	If no/not all documents address IVDs by tier: What are the reasons?	
	If no/not all documents address community tier: What are the reasons?	
**Stakeholders, processes and criteria for prioritizing / selecting IVDs**	Category of stakeholders involved in development of documents addressing IVDs	#2 : Le DRH du Ministère de la santé pare que c'est lui qui affecte le personnel dans ces formations sanitaires ou dans ces laboratoires ; il faudra donc qu'il soit bien informé.<br><br>Ministère de l'enseignement supérieur : pour l'harmonisation des appellations des diplômes<br><br>Les représentants des écoles de formation parce qu'eux aussi devront bien être au parfum de ce qu'on attend en termes de compétences et de performances de ces personnels-là pour qu'on s'aligne tous sur la stratégie nationale de développement de santé.<br><br>Les professionnels de santé à tous les niveaux de la pyramide, ceux dont on a connaissance, ceux qui ont l'expérience et qui continuent habituellement à faire tout ce qui est stratégie et développement.<br><br>Les partenaires et les bailleurs<br><br>Que tous relisent tous les termes pour qu'on soit bien d'accord sur tous et surtout les aspects juridiques parce qu'on peut écrire une chose et juridiquement parlant ça n'a pas de sens ou alors ça contredit un existant ; c'est pour harmoniser le contenu et le contenant.<br><br>Pour l'atelier de lecture et d'amendement<br><br>À ce niveau c'est encore plus sélectif. Les directions qui interviennent, sont utilisatrices sont conviées, quelques partenaires, quelques acteurs de la société civile et même quelques professionnels de laboratoire.
	Steps in development of documents (*consultants, drafts, workshops?)*	Il y a deux approches :

		- Pour la première, le partenaire peut décider de recruter un consultant qui vient dans le pays récolter les données et développer le draft du document qu'on remet au pays.<br>- L'autre approche est de faire de petits groupes de travail pour sortir avec un draft.<br><br>Un partenaire peu appuyer un atelier de rédaction avec quelques personnes sélectionnées au choix qui selon un consensus peuvent apporter une contribution au squelette du document.<br><br>Une fois qu'on a un draft, on procède à ce qu'on appelle un atelier de validation où on invite un plus grand nombre de parties prenantes impliquées dans l'activité au pays et pendant deux ou trois jours ils reliront le document et y apporteront des inputs.<br><br>Après on passe à ce qu'on appelle un atelier d'adoption où on va le présenter au ministre de la santé.<br><br>Puis on organise une réunion où on va adopter le document, il va le signer et à ce moment maintenant on peut l'imprimer et le distribuer.<br><br>Mais si à la suite de l'atelier de validation il y a des éléments qui n'ont pas été complètement abordés mais dont on pense que ce n'est pas grand-chose, on va refaire de petits ateliers restreints pour affiner le document avant de l'envoyer au ministre pour l'adoption.<br>Certains documents passent au service du premier ministre ou à l'assemblée nationale.<br><br>Si le document a des textes qui ont des implications juridiques, il faudra qu'on l'envoie à la DAJC, la direction des affaires du juridique et du contentieux pour qu'elle vérifie par rapport aux textes et aux lois du pays si la formulation ne pose pas problème.<br><br>#2 : Il y a la relecture et la validation et ensuite il y a l'adoption qui est comme une publicité de ce qu'on a fait comme travail et c'est comme une réunion d'information au grand public mais juste quelques heures, et que la tutelle, notre ministre, dise officiellement que tel travail a été fait, je l'ai reconnu et validé et voilà désormais le document sur lequel vous vous appuierez pour faire x, y ou z.
	Criteria for selecting essential IVDs (by tier, clinical care or disease surveillance)	#1 : Calqué sur les ressources humaines présente dans la structure et les activités de soins qui y sont dispensées :<br><br>- Le système de santé qui dit que les centres de santé doivent avoir un laboratoire ambulatoire doivent avoir à leur tête un infirmier<br>- Quelles activités un infirmier peut-il mener ? Donc on calque ses activités, on transpose ça directement au niveau du laboratoire de ce niveau.<br>- Lorsqu'il y a la présence d'un médecin, ça veut dire qu'on est déjà au niveau du centre médical d'arrondissement, qui a un paquet minimum d'activités qui inclut déjà les analyses de laboratoire.<br>- Quand on va monter au niveau de l'hôpital de district il y aura en plus du médecin, des médecins spécialistes et le paquet minimum d'activités va augmenter avec les spécialités et ainsi de suite jusqu'au sommet, au niveau des hôpitaux généraux.<br><br>#2 : On s'est appuyé déjà sur le document de stratégie nationale de développement de santé comme on vous l'a dit et on a défini jusqu'à quel niveau de prise en charge chaque formation sanitaire peut recevoir un malade, au minimum dans la prise en charge : là on est dans la clinique, si on dit diagnostic rapide du test de paludisme, on peut chercher dans les cellules sanguines etc. donc on s'est appuyé sur un autre document déjà signé, valide du PSND et du plan de santé communautaire pour pouvoir établir ces normes-là.

		#3 : The criteria was based on the presence of the staffing capacity and the structural capacity. So if you look at some of the very small HCs, they all have a technician, a trained technician. Generally, they have maybe a nurse and that nurse is just trained to do rapid malaria test, rapid HIV.<br><br>So when you look at our structure you can see the different tiers they come with the HR capacity and the structural capacity. But some of them now are expanding so they have maybe medical biologist or they have a technician, technologist in those facilities and so they just maybe put some equipment there but the structure does not meet even the equipment that are placed there so that's the challenge.
	Discussions and (dis)agreements in prioritizing IVDs – by tier (*note: RDTs in community?)*	#1 : On essaie de revoir aussi si on peut s'aligner avec les exigences de l'OMS sur le National Diagnostic Essentials list. C'est quelque chose qui en train de se voir parce que même cette liste-là est obsolète ; la preuve en est qu'il y a des analyses qui sont réalisées sur le territoire national qui ne se retrouve pas sur la liste des analyses des différents niveaux de laboratoire et je pense aussi que la classification n'est plus adéquate parce qu'il y a les structures par rapport à leur plateau technique qui, sur la base de la classification administrative, seront classées comme des hôpitaux de district peut-être et pourtant ils ont déjà l'infrastructure des hôpitaux régionaux.<br><br>La DPLM est en train de travailler pour que la classification ne soit plus administrative mais plutôt sur la base des plateaux techniques des différentes structures et à partir de là, le privé pourra aussi définir le niveau auquel il appartient car si on reste sur la base de la classification administrative, le privé ne pourra pas s'y intégrer. Si on passe à la classification par plateaux techniques, ce sera plus facile pour chaque structure de s'identifier et de savoir à quel niveau elle se trouve dans la pyramide sanitaire.
**Intention and plans to develop NEDL *(processes in development of Nigeria NEDL)***	Knowledge about WHO EDL and perceived usefulness of WHO guidelines for developing NEDL?	#1 : Nous en avons déjà entendu parler ; elle n'est pas encore applicable mais je pense que la direction des laboratoires est en train de travailler dans ce sens comme on le disait pour réorganiser l'organiser l'organisation par niveaux des laboratoires.<br><br>Les documents que nous avons ne sont pas calqué directement sur cette liste essentielle de l'OMS.<br><br>#3: Hi, I attended a meeting some time ago I think that was 2017 in Addis and it was discussed. I participated in the development of some of those guidelines, which is a very good approach and it will not only help our weak supply chain system in limited resource countries, if we have a document it will really help us manage our supply chain, but now it doesn't exist so sometimes it's really challenging with the frequent stock out, we saw it during COVID when we could not have access to the shipment of commodities it was very challenging. With our EDL, we would be able to plan in advance and so on...Why not we can start having some of those decentralizing manufacturing some of them, not only in Southern Africa, in East Africa but we should also have it in West and Central Africa.
	MOH's taken and/or planned steps for developing NEDL or similar document (who took the initiative, survey, who writes, stakeholders involved, validation etc)	Pas encore de plan pour développer une LDE au Burkina Faso<br><br>« Comme je vous disais, l'élément déclencheur, ce sera l'adoption de la politique nationale de laboratoire car à l'intérieur toutes les déclarations de politique sont faites, c'est-à-dire que tout ce que nous allons élaborer doit prendre ancrage dessus ».
	Perception of usefulness of NEDL in solving (some) barriers to availability of IVDs in tiers / lab system	#1 : Peut aider à améliorer notre système de laboratoires.

	Present and foreseen challenges in development of NEDL	
**Implementation of NEDL**	Plans and steps for implementation	
	Which stakeholders are /will be involved in implementation	
	What problems in lab services will/may be solved with successful implementation of NEDL?	
	(Foreseen/possible) Problems in implementation of NEDL and how to solve these problems	
**Recommendations**	For own country: steps to take in developing and implementing NEDL by tier	#3: My recommendations will be to first of all I think you said this assessment has been conducted with several countries, so I think it's important to know what are their commonly used diagnostics around before publishing. I think it will then reduce the cost. Because if many countries are using the same thing then production will be cheaper and I think we will have a lot of cost efficiencies.<br><br>#1 : Il faut prendre en compte c'est le plateau technique du laboratoire, ne pas s'attarder sur le document administratif : sur le terrain, on rencontre des situations sur le terrain où vous avez un laboratoire qui est sous-classé, qui a un plateau technique qui ne cadre pas avec son niveau dans la classification<br><br>So you know, as I said we haven't done it as a country but it's a good thing to have. So my recommendations would be first of all if there can be an assessment in country of what is frequently used at the level of diagnostics and even prescriptions, then we can think of establishing such a list, but the overall goal is that it's important to have that list but I cause we have not done it I cannot give you the steps to succeed it.
	For other countries: steps to take in developing NEDL by tier *(Nigeria's lessons)*	
	For development of NEDL: considering gender of recipients	#1 : Pour le moment selon notre contexte, je pense que c'est prématuré.<br><br>Je pense que s'il faut revoir par exemple le cas du paludisme où il y avait l'accès à un paquet minimum pour les enfants de 0 à 5 ans et puis les couches défavorisées, je pense qu'on peut également le faire pour les analyses, et certains tests pour des couches vulnérables.<br><br>Pour les femmes, je ne pense pas trop mais c'est-à-dire que pour moi ce sont les couches défavorisées.
	For ASLM / FIND/ WHO: type of support for countries in development and implementation of NEDL	#3: But as CDC we have a role to provide technical assistance, an advisory role to the government. So we will provide that role, give them the infos but the final decision will come from them. You have already talked about WHO, ASLM, I think those are even at a better place to advise all the governments, yes our role as CDC we are really not...we don't do a lot of that but we provide a kind of advice especially using our data to inform so but WHO and ASLM I think they have the role you need to really advise, give kind of strong recommendation.

INTERVIEW PARTICIPANTS FOR COUNTRY: ETHIOPIA

# INT	Place of work participants
1	Laboratory Services MOH, at Ethiopian Public Health Institute (EPHI)
1	Laboratory Services MOH, at Ethiopian Public Health Institute (EPHI)
1	Laboratory Services MOH, at Ethiopian Public Health Institute (EPHI)
2	Laboratory Services MOH, at Ethiopian Public Health Institute (EPHI)

DOCUMENTS THAT ARE ANALYSED

# DOC	Name document, year
1	National strategic TB and leprosy plan 2013-2022 (midterm 2017 and then revised)
2	HIV strategic plan 2015-2020
3	National strategic plan for malaria prevention, control and elimination in Ethiopia 2011-15 Draft
4	Master Plan For The Public Health Laboratory System In Ethiopia; Second Edition; (2009 – 2013)

FINDINGS BY THEME

Global themes	Specific themes	Findings/Answers (*Refer to # INT and # DOC*)
**MoH organisation**	Position laboratories in MoH *(separate department or sub-division?)*	Laboratory Services is one of the three strategic focus areas of the Ethiopian Public Health Institute (EPHI) which is autonomous public authority in the ministry of health (Others: public health emergency; public health research). #1: EPHI serves as laboratory directorate for MoH<br>The Ethiopian Public Health Institute (EPHI) is the result of the merger in April 1995 of the former National Research Institute of Health (NRIH), the Ethiopian Nutrition Institute (ENI) and the Department of Traditional medicine (DTM) of the Ministry of Health. The merger was affirmed by the council of ministers regulation No 4/1996, which recognized the Institute as an autonomous public authority having its own legal personality.<br>Website EPHI: Mission: To improve the quality of health care service by establishing quality laboratory system and building the capacity of national and regional clinical and public health reference laboratories<br>#0: EPHI is involved in all plans, for instance in TB policy.<br>#2: Labs are categorized: 1) the coordination unit: national lab capacity building directorate; 2) National Reference Labs, that provide reference testing services. The capacity building directorate is working on the overall coordination and the oversight of the national labs programs but there is also the technical arm of the coordination unit. [the structuring document is not ready yet – maybe in two months]
**Laboratory system**	Tiers in laboratory system	#2: Four tiers: First is the (NRL) national reference labs at EPHI; 2nd RRL (regional reference lab) (1st and 2nd include are federal health initiative including the uniformed force lab, and central blood bank lab); 3rd Hospital labs , including regional, zonal and district hospital labs; 4th HC lab including health post labs. However, labs at HC and HP minimal.<br>#1: : Health extension workers and at health posts do malaria RDTs and pregnancy tests. #2: HC lab staff controls quality of RDTs done by HP and CHEWs who report every two weeks to HC level. HP and CHEWs trained by HC lab professionals.<br>DOC1: HP: refer for TB tests to Health centre.
	Laboratory tiers related to tiers health system (*including community level*)	Three tiers health care pyramid, starting at Woreda level: primary hospital (with catchment population of 60,000-100,000), health centres (1 per 15,000-25,000 population) and their satellite Health Posts (1 per 3,000-5,000 population) linked through referral systems, which forms a Primary Health Care Unit (PHCU); general hospital; specialized hospital. Private for profit and NGOs/FBOs augment health service coverage and utilization at all levels.DOC1).<br>#1: around 4000 health facilities including 271 hospitals and 3541 health centres with distribution based on the population size; availability coverage is around 100%. However,

		there may be problems with accessibility because of challenges in supply chain or personnel turnover and many other factors
	Distribution of laboratories across public, private for profit / not for profit	Private for profit and NGOs/FBOs augment health service coverage and utilization at all levels
	Challenges in laboratory system, differentiating for tiers and areas *(problems in availability and access to – specific - IVDs)*	#2: Programmes fund labs and tests for HIV, Malaria, TB and cancer, government funds other tests [not clear whether this is considered a problem – I assume so]<br>#2: HC and HP have minimal labs – no lab professionals<br>#1: Careful to identify problems, because need assessment for NEDL first. Only refer to over- and understocking, and erratic procurement / supply.<br>DOC1: TB and leprosy programme heavily depends on donors for funding. Insufficient lab workers. Limitations in lab system hinders TBP:<br>• Gaps in specimen referral and transportation.<br>• Weak laboratory information and data management system: limited routine use of standardized sample recording and reporting tools, poor test results feedback to referring facilities and lack of routine reporting of TB culture and DST results to EPHI and FMoH.<br>• Lack of enforceable technical regulatory mandate of EPHI over regional or peripheral laboratories.<br>• Equipment maintenance is centralized, with limited system for rapid response.<br>• Lack of backup TB culture and DST specialized equipment and spare parts.<br>• Gaps in reagent preparation for microscopy.<br>• Limited coordination between NTP and EPHI; RHBs and regional laboratories.<br>DOC2: See as critical enabler for HIV programme 1: Health system strengthening: HMIS/M&E, PHPM & Laboratory services. Present problems: Inadequate laboratory services. Frequent disruption of laboratory services particularly CD4 testing has been observed in ART sites due to broken machines, lack of trained laboratory technicians or power disruption to run equipment. Maintenance of HIV related equipment was centralized and even then there was no adequate capacity at the center to provide timely maintenance services. Detection of treatment failure was low. Lack of standardized guidance on patient identification for second line, the required capacity and adequate Laboratory facilities (viral load) largely contribute to the low detection of treatment failure. Noticed problem of central maintenance structure: should be decentralized.
**Stakeholders in lab system and services**	Technical	
	Implementing	
	Funding	#2: GF (TB and malaria), World fund, America CDC, Africa CDC, FIND. Some funders specifically for vertical programmes.
	MoH departments / states / regions	
**National committee/working groups on IVDs**	Committee name(s) and aims	#1: Not yet real committee, but team of 4 persons in working group from EPHI assigned by director of National Laboratories Capacity Building Directorate to make a draft NEDL
	Categories of committee members, % women	All from National Laboratories Capacity Building Directorate
	Who decides on the members of these	Director of National Laboratories Capacity Building Directorate

	committees and what are criteria for membership	
	Missing stakeholders in committees?	
	If no committee: what the MoH plans/ what are barriers ?	
**Presence of national laboratory documents**	Laboratory policy (*Look at date*)	#2: Draft Revised National Lab policy and strategic plan. However, because the government is restructuring and reforming some federal organizations, finalizing the policy and plan has to wait for finalisation of the restructuring process.
	Strategic plan<br>*(Look at date, budget?)*	
**Presence of documents with guidelines on IVDs**	National documents that address (essential) IVDs (*dates, budget?)*	1. National Strategic Plan Tuberculosis and Leprosy Control – 2013-2022 (With tiers, budget, referral, identification of problems, also in laboratories, steps and stakeholders in development<br>2. HIV/AIDS STRATEGIC PLAN 2015-20<br>3. National strategic plan for malaria prevention, control and elimination in Ethiopia 2011-15 Draft (Tiers, Community, budget, stakeholder<br>4. MASTER PLAN FOR THE PUBLIC HEALTH LABORATORY SYSTEM IN ETHIOPIA; Second Edition; (2009 – 2013)
	What vertical (disease) programs identify priority IVDs ?	DOC1 (TBL)<br>DOC2
	Whether documents define (essential) IVDs by tier – if so, which tiers? (including community level)	DOC1: yes<br><br>DOC3: yes including community. HEWs have been trained on the use and interpretation of results of multi-species RDTs. A Laboratory-Based Quality Control Testing of Malaria RDTs in Ethiopia has recently been introduced and will ensure the procurement and use of quality assured RDTs.<br><br>DOC4: Yes, in Appendix 1: Minimum Laboratory Testing Services at Each Tier of the Laboratory; tables for three tiers: chart of lab tests by level with examples of equipment and vendors for each test. Appendix 2: Minimum Requirements for Laboratory Equipment at Each Tier of the Laboratory (table with equipment, what tests the equipment does and presentation of three models
	If no/not all documents address IVDs by tier: What are the reasons?	
	If no/not all documents address community tier: What are the reasons?	
**Stakeholders, processes and**	Category of stakeholders	#2: Process needs special support including the technical and the financial support and also it need resource mobilization. Before implementation we arrange some discussion

**criteria for prioritizing / selecting IVDs**	involved in development of documents addressing IVDs	and we try to accommodate and incorporate all level of stakeholders, funders, regulatory bodies, international and local stakeholders' interests.<br>DOC3 (malaria): funding through its GFATM Round 8 proposal, and benefits from a strong partnership that is expected to continue its funding commitment: The Carter Center (TCC), WB, USAID (including PMI), WHO, UNICEF, UNITAID, MACEPA, PSI. All the partners buy into the National Strategic Plan, and the MCST ensures that activities are not duplicated.
	Steps in development of documents (*consultants, drafts, workshops?)*	For Doc1: Consultants wrote draft
	Criteria for selecting essential IVDs (by tier, clinical care or disease surveillance)	#2: Disease burden; availability of resources; technology; trained manpower - actual situation on the ground according to the Ethiopian level.
	Discussions and (dis)agreements in prioritizing IVDs – by tier (*note: RDTs in community?)*	
**Intention and plans to develop NEDL *(processes in development of Nigeria NEDL)***	Knowledge about WHO EDL and perceived usefulness of WHO guidelines for developing NEDL?	#1: Yes, use EDL2 for draft NEDL. Had to adapt to their health system tiers and also have stand-alone labs. They specified for community level and health facilities without labs: pregnancy tests and malaria diagnosis RDT. Decided: HIV RDT at community level not feasible; they rely on labs in health centres
	MOH's taken and/or planned steps for developing NEDL or similar document (who took the initiative, survey, who writes, stakeholders involved, validation etc)	#0: Director National Laboratories Capacity Building Directorate, Ethiopian Public Health Institute (EPHI) has assigned three persons to develop NEDL, there is a document, but not well organized. They collected the info from various other documents, such as food and drug documents, advanced lab standards, laboratory master plan that was already developed by EPHI in 2009, minimum health services package.<br>#1: Previous director, initiated one year ago, probably initiative originally came from the Ministry of Health. Team of 4 (2 women, two men) drafted the NEDL<br>#2:<br>NEDL in workplan of directorate. Process needs special support including the technical and the financial support and also it need resource mobilization. So before implementation we arrange some discussion and we try to accommodate and incorporate all level of stakeholders, funders, regulatory bodies, international and local stakeholders' interests.]. As reference the WHO EDL (second edition) and the Indian NEDL.. Already allocated some resources for this program from the American CDC lab program, we have some workshops and a document paper preparation we will cover their cost until they develop this document with the different stakeholders. For finalizing draft need discussion platform to accommodate stakeholders, national and international funders. Then send this document to the MOH for their approval and critical review before implementing this document. After approval this document will be sent to the Ethiopian standard agency for publication and distribution for this document to reach the Ethiopian HS level.]<br>They then engaged stakeholders, experts from national reference laboratories to draft the first list of essential diagnostics which included specific disease and the departmentalized list of diagnostics. Reference labs were: National TB reference laboratory, the parasitology laboratory, Malaria and Neglected Tropical diseases, experts laboratories were also involved in this list development and experts from microbiology laboratory, from HIV laboratory also. We have included Non-communicable diseases, experts from the national clinical chemistry laboratory. We have also experts from EPSA (Ethiopian Pharmaceutical Supply Agency) laboratory (MoH government agency). (The Ethiopian Pharmaceutical

		Supply Agency (EPSA) is responsible for supply chain management of public health commodities in Ethiopia. The agency has 19 branch warehouses that serve more than 3,800 health facilities that in turn serve 105 million people in nine regional states and two administrative states.)<br><br>In their pharmaceutical procurement list 2018 chapter 2 is List of Laboratory Reagents, Chemicals and Supplies: (but not by tier of the health system)<br><br>The experts from disease programmes like TB, malaria and HIV included the tests that are needed for their programmes in the draft NEDL.<br><br>They included the specific tests that are already available in the country, they got this information from different reference laboratories and experts - not looked at what is available globally. Now it is a matter of assessing whether these tests can actually be performed in health facilities, considering local circumstances. (checklists on available machines, personnel etc).<br><br>Then conducted 2 workshops to develop these lists (????). They did not involve WHO or other technical or funding agencies, but are planning to do so in future. All till now was paid by EPHI.<br><br>Criteria for inclusion in the list are important diseases common in the country recommended by WHO. They get data on epidemiology from the MoH and will get it from their assessment of health facilities. They now have to start the stage of doing an assessment to see the status of the availability and the accessibility of these list in the country. They have not started – need funding for the assessment. So steps: have made a draft list and will now do assessment.<br><br>They have made a checklist for the assessment: including common diseases, infrastructure, staffing, expertise. For the assessment they need ethical clearance from the Scientific Ethical Review office and of course have to look for funding.<br><br>Funding for assessment could come from USAID, KNCV, CHAI, FHI. These donors also worked on disease specific programmes that need support from labs for diagnosis, surveillance etc. (indeed in disease specific programmes they talk about importance of strengthening labs)<br><br>They plan to have the final draft list ready end 2022 or early 2023.<br><br>The next step is to involve all stakeholders in the area to more develop the documents<br><br>They need funds for a consultant to do the writing and for a dissemination workshop cause we need to disseminate the document and we need to raise awareness and then this part will be the implementation. Awareness needs to be raised from stakeholders working in regional health bureaus.<br><br>They also used as a reference the lists of the Ethiopian Standard Agency, with standards for medical devices, medical tests, physical structures and staff for each level of health facility (a huge document). There are standardized requirements for facilities to get a license to provide services. They also have standards for laboratories and diagnostics. In the NEDL they will prioritise IVDs from the standard list, after they have done the assessment of what is available in the health facilities, the prices and accessibility for the community.
	Perception of usefulness of NEDL in solving (some) barriers to availability of IVDs in tiers / lab system	#1: The NEDL will help health facilities to prioritize the resources they have and to serve their communities and prevent recent over-stocks for some of the test and be out of stock for the others, even for some important tests. The NEDL shows what amount of IVDs and other supplies they need and the list of resources that will be allocated to them. Based on this they will manage the supply chain, their supply stock. We hope this way they can manage their supplies
	Present and foreseen challenges in development of NEDL	

Implementation of NEDL	Plans and steps for implementation	
	Which stakeholders are /will be involved in implementation	• Pharmaceutical Fund and Supply Agency (PFSA) - in procurement and supply management.<br>• Food Medicines Healthcare Administration and Control Authority (FMHACA) for in country medicine and products registration, regulatory agency, quality of medicines, active drug safety monitoring and management (aDSM). Website:<br>• EFDA is the National Regulatory Body of Ethiopia which is under the Ministry of Health. The Authority is responsible to ensure the quality, safety and/or efficacy of medicines, food, cosmetics and medical devices. To promote and protect the public health by ensuring safety and quality of products and health service through registration, licensing and inspection of health professionals, pharmaceuticals food establishments and health institutions and provision of up-to-date regulatory information while promoting rational medicine use<br><br>DOC3: (for malaria: . Pharmaceuticals Supplies Service. The establishment of an agency entirely responsible for drugs and supplies procurement and distribution (i.e. PSA) is an outcome of the sector wide reform which will bring about dramatic improvement in logistic management. Anti-malarials and diagnostic supplies will be procured at the national level through PSA and distributed directly to health facilities. (Agency handles all malaria supplies issues); DACA (Drug Administration and Control Agency): involved in the registration and approval of anti-malarial supplies. Within the new BPR structure (see below), DACA's regulatory mandate has increased to health professionals and health facilities.
	What problems in lab services will/may be solved with successful implementation of NEDL?	
	(Foreseen/possible) Problems in implementation of NEDL and how to solve these problems	#2: Funding of procuring the IVDs: Some programme specific tests will be funded by programmes, Others by govt of Ethiopia. ''We are living in developing countries ''.
**Recommendations**	For own country: steps to take in developing and implementing NEDL by tier	
	For other countries: steps to take in developing NEDL by tier *(Nigeria's lessons)*	#1 All countries should have these document based on their own scenario, based on their own disease epidemiology, and based on their health facilities, their own health facility structure, the available laboratories, they need to have this list so it may take different approaches, because they may have different factors to be considered in the prioritization of this essential list .
	For development of NEDL: considering gender of recipients	#1 They did not prioritise test by gender, but there are tests that are included in the list that gender specific for example STD for maternal health, and the other testers for cancer marker, cervical or breast cancer and the like. The tests important for this purpose are included in the list. When we ask the female member whether she thinks gender is important in decision making and prioritization she says: ''I think a there's no issue regarding the gender; I cannot talk of the value of considering gender in this decision because it's not that much issue in this area. I think disease diagnosis is general not only for females. ''

	For ASLM / FIND/ WHO: type of support for countries in development and implementation of NEDL	#1: ASLM to fund the assessment. [they need funding before submitting to IRB). [We advise to write a proposal and submit]

INTERVIEW PARTICIPANTS FOR COUNTRY: **KENYA**

# INT	Place of work participants
**1**	Laboratory Services MoH
**1**	Other Dept MoH
**1**	Hospital laboratory
**1**	Laboratory Services MoH
**1**	Laboratory Services MoH
**1**	Hospital laboratory
**2**	Laboratory Services MoH
**3**	Regulatory Body
**4**	Partner
**5**	Partner

DOCUMENTS THAT ARE ANALYSED

# DOC	Name document, year
**1**	Kenya Essential Medical Laboratory Commodity List 2014; Published by the Ministry of Health, February, 2014

FINDINGS BY THEME

**Global themes**	**Specific themes**	**Findings/Answers (*Refer to # INT and # DOC*)**
**MoH organisation**	Position laboratories in MoH *(separate department or sub-division?)*	**The Department of laboratory service includes two divisions (of personnel and of diagnostic and clinical support) and is under the authority of the Directorate of public health.**<br>#1<br>1. Ministry of Health<br>2. Cabinet Secretary<br>3. Permanent Secretary<br>4. Director General of Health<br>5. Directorate of public health<br>- Department of laboratory service<br>o Division of personnel<br>o Division of diagnostic and clinical support
**Laboratory system**	Tiers in laboratory system	**Four tiers of laboratories including community level with household units.**<br>#1:<br>Tier 1: Community (household units)<br>Tier 2: Dispensaries; Health centers<br>Tier 3: County hospitals; Sub-county hospitals<br>Tier 4: MTRH; KU; KNH; Reference labs
	Laboratory tiers related to tiers health system (*including community level*)	**There is six level in Kenya health system.**<br>#1:<br>Level 1: Community (household units)<br>Level 2: Dispensaries<br>Level 3: Health centers<br>Level 4: Sub-county hospitals<br>Level 5: County hospitals<br>Level 6: MTRH; KU; KNH; Reference labs
	Distribution of laboratories across public, private for profit / not for profit	Not asked

<table>
<tr><td></td><td>Challenges in laboratory system, differentiating for tiers and areas (problems in availability and access to – specific - IVDs)</td><td>Many challenges related to human resources (lack of qualification), equipment, high cost of testing, particularization of programs, insufficient budget for laboratories. Non-adoption and non-dissemination of Kenya essential laboratory commodities list.

#1:

- Difficulty with equipment leases
- Defective machines,
- Lack of qualified personnel,
- Insufficient budget

#2:

- Problems in labs with the consumables, machines, HR, etc etc. – NEDL can help to streamline and plan procurement.
- There were problems with former list Kenya essential laboratory commodities list (2014), because it was not spread, was not endorsed and taken up.
- Problems with budgets: with a good list they can also better plan for procurements. And see what partners bring in and what MOH can buy.
- Some conflict with PPB which is the regulatory body now.

#3: The laboratory services department were not so happy with their appointment, with the work having been done by KMLTTB (The Kenya Medical Laboratory Technicians and Technologists Board). Laboratory services thought this work should be done by laboratory scientists, not by pharmacists.

#4:

- Lack of resources,
- High cost of testing

#5: The Laboratory is not supported, it lacks structural support within the government

The particularization of programs has not helped the laboratory system</td></tr>
<tr><td rowspan="4">Stakeholders in lab system and services</td><td>Technical</td><td>#1: PEPFAR (CDC/USAID), WHO</td></tr>
<tr><td>Implementing</td><td>#1: ASLM, Africa CDC</td></tr>
<tr><td>Funding</td><td>#1: Global Fund, World Bank, CHAI</td></tr>
<tr><td>MoH departments / states / regions</td><td>#1: National AIDS/STI Control Program (NASCOP), TB program, National Cancer Control Program (NCCP), Malaria Program, Disease Surveillance and Response, Influenza Program, KEMSA, MEDS, KMLTTB, PPB

#3 : PPB: Roles and responsibility of this regulatory body

1. Pharmacists and technician and nurses training accreditation and continuous professional development and including CPD. Their licence, to be renewed annually is based on this.
2. Standards for premises
3. Products including medical devices and technologies. IVDs are categorized here
4. For IVDs PPB has all the regulation functions, including: Licensing, marketing authorization.</td></tr>
</table>

**National committee/working groups on IVDs**	Committee name(s) and aims	**No committee. A small ad hoc committee was once created and then dissolved after developing a document (list)**<br><br>#1: There is no committee. For the existing document, this was made by a small *ad hoc* committee that once that document ended that committee was disbanded.
	Categories of committee members, % women	Not asked
	Who decides on the members of these committees and what are criteria for membership	Not asked
	Missing stakeholders in committees?	Not asked
	If no committee: what the MoH plans/ what are barriers ?	Not asked
**Presence of national laboratory documents**	Laboratory policy (*Look at date*)	Any
	Strategic plan<br>*(Look at date, budget?)*	Any
**Presence of documents with guidelines on IVDs**	National documents that address (essential) IVDs (*dates, budget?)*	**Kenya Essential Medical Laboratory Commodity List; Published by the Ministry of Health, February, 2014**<br><br>Problems why it was not taken up and not revised:<br>• No dissemination to implementers<br>• Was not anchored well in MOH leadership organs, from national to the county level<br>• Document was made before devolution – that is when county governments are the second level of government, with their own roles. Before plans and policies were valid from national to lower levels. Now you have to involve them all.
	What vertical (disease) programs identify priority IVDs ?	
	Whether documents define (essential) IVDs by tier – if so, which tiers? (including community level)	#1: **Yes, document define IVDs including the community level**<br><br>The lowest level are communities which were tier 1 and, in the communities, we came out with a list of in vitro diagnostics that is done at community level which means that they have household units and you'll get mostly the mobile they visit house to house.<br><br>The mobile teams composed of Community healthcare workers (CHW) they visit households to do malaria in vitro diagnostics, random blood sugar, sometimes HIV and self-testing
	If no/not all documents address	

	IVDs by tier: What are the reasons?	
	If no/not all documents address community tier: What are the reasons?	
	Category of stakeholders involved in development of documents addressing IVDs	**Laboratory Diagnostic Services Unit (LDSU) and the National Public Health Laboratory Services (NPHLS) for the development of the document. KEMRI, KNH, UON, AMREF & KMTC. MSH for technical support. USAID for financial support.**<br>Doc 1: This document was produced with the support of Management Sciences for Health/ Health Commodities and Services Management Program (MSH/HCSM) through USAID funding<br>The Ministry of Health acknowledges the contribution of the various institutions and the funding and implementing partners for their support towards the development of the Kenya Essential Medical Laboratory Commodities List (KEMLCL). The Laboratory Diagnostic Services Unit (LDSU) and the National Public Health Laboratory Services (NPHLS) steered the process of the development of KEMLCL document to its conclusion. We further appreciate the contribution by the various technical working groups (TWGs) and in particular the secretariat that comprised of the heads of Ministry of health laboratory divisions and units. As well as the contribution of Institutions (KEMRI, KNH, UON, AMREF & KMTC) and the technical support of MSH under the HCSM program.<br>Finally, we acknowledge the technical and financial support by the United States Agency for International Development (USAID) through MSH/HCSM for the development and publication of KEMLCL.
**Stakeholders, processes and criteria for prioritizing / selecting IVDs**	Steps in development of documents (*consultants, drafts, workshops?)*	**Document developed by a laboratory working group first. The document was shared with stakeholders. But, finally the document was not endorsed.**<br>#1: A work group first<br>Then shared with other stakeholders<br>It was not endorse, it was not taken up cause, they didn't really see their interest
	Criteria for selecting essential IVDs (by tier, clinical care or disease surveillance)	**Driving criterion is presence of equipment. Lab capacity in terms of personnel. Sometimes even in higher level hospital they do still lower level tests (VCT) when it is a service delivery point in a room. Disease burden in different counties and in the priority diseases in the country. Cost of tests. Connected to EML (that is updated annually)**<br><br>Gender: Cervical and breast cancer are on the list<br><br>Doc 1 : Inclusion of a test or commodity on the KEMLCL is considered if the test or commodity, meets the following criteria:<br><br>1. Relevance/Need: contribution towards meeting the identified priority health care needs of the population<br>2. Safety: Scientifically proven and acceptable in its expected way of use for health care workers and patients.<br>3. Quality: The products should comply with internationally acceptable quality standards, as recognized by the Kenya Medical Laboratory Technicians and Technologists Board or other duly recognized regulatory body. The standards should include stability under expected conditions of storage and use.<br>4. Performance: sensitivity and specificity should be meet the WHO requirements for each product indicating the percentage for each commodity/supply<br>5. Comparative cost-benefit: a favorable cost-benefit ratio (in terms of use) compared with alternative products.

		6. Local Suitability/Appropriateness: Preference should be given to a test or supplies with which (laboratory staff is well familiar) and that are suitable and reliably available in the local setting.<br>7. Local Manufacture: To improve availability, and possibly, reduce costs, the test or commodity should have the possibility of being manufactured locally<br><br>#1 :<br><br>• Driving criterion is presence of equipment<br>• Lab capacity in terms of personnel<br>• Sometimes even in higher level hospital they do still lower level tests (VCT) when it is a service delivery point in a room.<br>• Disease burden in different counties and in the priority diseases in the country<br>• Cost of tests<br>• Connected to EML (that is updated annually)<br>• Gender: Cervical and breast cancer are on the list
	Discussions and (dis)agreements in prioritizing IVDs – by tier (*note: RDTs in community?)*	
**Intention and plans to develop NEDL *(processes in development of Nigeria NEDL)***	Knowledge about WHO EDL and perceived usefulness of WHO guidelines for developing NEDL?	**List is not used in Kenya. Look in products list of WHO for quantification or projection for HIV.**<br><br>#1: When you are procuring when you are making quantification and projection for HIV commodities we look at the entire product listing WHO<br><br>#4: We don't use that here in Kenya
	MOH's taken and/or planned steps for developing NEDL or similar document (who took the initiative, survey, who writes, stakeholders involved, validation etc)	Not yet
	Perception of usefulness of NEDL in solving (some) barriers to availability of IVDs in tiers / lab system	**This list would assist in planning at all levels, at the national level. The list would now even help counties allocate appropriate resources. It will regulate private sector. It would bring standardization in terms of quality management system.**<br><br>#1:<br><br>- It will sort out the issue of standardization in terms of technologies<br>- So once standing guidance is that also this NCD like the renal, the kidney you can see they have done a lot of renal support, so also the cancer and other. It will reduce out-of pocket expenses for patients.<br>- This will regulate private sector<br>- This will review technology and tests that are done presently<br>- This would bring standardization in terms of quality management system<br><br>#3 :<br><br>- Procurement efficiency

		- Expanding coverage in society, with counties and health facilities knowing which essential IVDs to procure<br>- Good for evaluation<br>- The essential IVDs on the list will be given a priority by the regulatory board<br>- The list will define what can be done at what tier after assessment of the level of expertise available at that tier.<br>- There is a need for Health technology Assessment to be able to assess the economic value of everything and put it in a list.<br><br>#4: This list would assist in planning at all levels, at the national level<br><br>The list would now even help counties allocate appropriate resources<br><br>The list will also guide many of the people making these decisions who are not scientists.<br><br>Such a list will communicate even to non-scientists, to scientists, to planners, and once you plan, planning will go hand in hand with resource allocation.
	Present and foreseen challenges in development of NEDL	**Better talking about diagnoses, not diagnostics. Better to talk about priority diagnostics but essential diagnoses for priority diseases**<br><br>#2: There is a National essential medicine list which is updated annually – this list can be used for the NEDL<br><br>There is a long list of diagnostics available in the country – we should prioritise the most essential by tier. Two lists should be considered. One NEDL and another list for other diagnostics to be planned by level that are not essential but that the health care structures can procure if they can afford.<br><br>In the NEDL we should talk about diagnoses, not diagnostics, because you also need materials, consumables and machines – these should be part of the NEDL<br><br>For all tests there should be standards on what is needed (also consumables etc) and the set of what is needed should be available, look at perishable issues – if not all consumables are available, there is no use to put the reagents on the list.<br><br>There should also be attention to diagnoses that are obsolete: especially in private clinics they use tests that are not supposed to be used anymore (I tell: in the WHO EDL there is a list of such tests attached)<br><br>Do not talk about *priority* diagnostics but *essential diagnoses for priority diseases*, considering the local and national burden of disease. ''Priority list should not be a wish list''
**Implementation of NEDL**	Plans and steps for implementation	Not asked
	Which stakeholders are /will be involved in implementation	Not asked
	What problems in lab services will/may be solved with successful implementation of NEDL?	Not asked
	(Foreseen/possible) Problems in implementation of	Not asked

	NEDL and how to solve these problems	
**Recommendations**	For own country: steps to take in developing and implementing NEDL by tier	**Include all stakeholders based on mapping. Engage MoH and governor's council. Identify a technical working group for document draft. Distribute document to stakeholders for inputs.**<br><br>#1: We make sure that all the stakeholders are included.<br><br>We set up a technical working group<br><br>We make sure that all the processes, the mapping of important processes like MOH engagement, governor's council engagement to engage their counties because they are stakeholders in this particular part.<br><br>First step is that we looked at our policy and then start with the reference documents and the EDL<br><br>We start with this technical work group that will draft a discussion paper that will be distributed to key stakeholders before we hold an engagement.<br><br>Then that document is reviewed both technically and in terms of scope by stakeholders<br>#2: This list has to be made by a small technical team first that collects list from priority disease programs<br>Then spread gradually wider to other stakeholders<br><br>#4:<br><br>Put together different stakeholders<br><br>Begin by conducting a literature review and obtaining information on diseases<br><br>Develop a zero draft, and then call the stakeholders.<br><br>#5: The process has to be inclusive, it has to be participatory considering the different levels of the health system.
	For other countries: steps to take in developing NEDL by tier *(Nigeria's lessons)*	**Engage World Health Assembly, regional committees (ECSA ECOWAS) and civil society.**<br><br>#4: We need to take this issue to the World Health Assembly.<br><br>Then we also have to look at other organizations like ECSA, the East African Community. West Africa also has its community.<br><br>CDC Africa can also push because they also discuss with the ministers.<br><br>Involve civil society
	For development of NEDL: considering gender of recipients	Not asked
	For ASLM / FIND/ WHO: type of support for countries in development and	**Contribution in the document development for PPB. Technical assistance for WHO**<br><br>#3 :<br><br>– Participating in the document development either by own staff or consultants appointed by the board

	implementation of NEDL	– Involvement in sensation of users (counties), and including procurement organization, in collaboration with Labs<br>– Train procurement people on the NEDL<br>– They will work with Dr Kiiru (of Laboratory services department)<br>– They will do post market surveillance of IVDs<br><br>#4: Technical assistance.<br><br>We may not be able to provide direct funding, because you see, this is like I said the Donor has their interests and our interests is HIV and TB.

INTERVIEW PARTICIPANTS FOR COUNTRY: NIGERIA

Interview #	Place of work participant
1	Medical Laboratory Service Division, FMOH
1	Medical Laboratory Service Division, FMOH (ex)
1	Medical Laboratory Service Division, FMOH
2	Partner - Consultant NEDL
2	Partner - Consultant NEDL
2	Partner - Consultant NEDL
3	Professional association
4	Hospital laboratory
5	Partner

DOCUMENTS THAT ARE ANALYSED

# DOC	Name document, year
1	Nigeria National Essential Diagnostic List 2021
2	Malaria strategic plan 2014-2020
3	Second National strategic health development plan 2:
4	Nigeria National Laboratory Services policy 2021-2025 (before 2015-2019) (not in QUANT)
5	Nigeria National Laboratory Strategic Plan 2021-2025 (draft – not for reference- not in QUANT))
6	Medical Laboratory Science Council of Nigeria: Guidelines for In-Vitro Diagnostics 2018. [guidelines how to regulate IVDs, not the actual list of IVDs; not in QUANT)

FINDINGS BY THEME

Global themes	Specific themes	Findings/Answers (*Refer to # INT and # DOC*)
**MoH organisation**	Position laboratories in MoH *(separate department or sub-division?)*	The Medical Laboratory Service Division (MLSD) is under the Department of Hospital Services. MLSD houses the secretariat of the National Laboratory Technical Working Group (NLTWG)<br><br>The Medical Laboratory Service Division (MLSD) is under the Department of Hospital Services. MLSD houses the secretariat of the National Laboratory Technical Working Group (NLTWG)<br><br>Two major groups of laboratories operating nationally and sub-nationally in case management and disease control- 1) the clinical (or medical) and 2) public health laboratories. Presently CDC Nigeria has a coordinating role in creating laboratory networks for some diseases of public health importance [e.g. yellow fever, CSM, measles, rubella, cholera, viral haemorrhagic fevers (Lassa fever, Ebola), monkeypox, influenza, COVID-19 etc],
**Laboratory system**	Tiers in laboratory system	1) the clinical (or medical) and 2) public health laboratories.<br><br>Clinical laboratories connected to health facilities, five tiers (Nigeria National Laboratory Services policy 2021-2025 Appendix III)<br>1) PHC centres with minimal lab services;<br>2) Model/Comprehensive Health Centre: with Basic laboratory services such as urine microscopy, urinalysis, malaria parasite (MP), haemoglobin concentration (Hb), and stool microscopy;<br>3) Clinics, Maternities and Nursing Homes;<br>4) Private Hospitals, Private Laboratories and General Hospitals;<br>5) Specialist Hospitals and Federal Medical Centres:
	Laboratory tiers related to tiers health system	Tiers of health care :<br><br>• Community: community health workers and primary centres, including PHC centres without labs do RDT for malaria

	(*including community level*)	• Primary: Ward Health System with (1) Health Post (2) Primary Health Clinic and (3) Primary Health Care Centres. PHC centres are supposed to have a laboratory to support malaria treatment (microscope) – however some have no lab (88.1% (30,109) of health facilities)<br>• Secondary: general hospitals should have a lab with three sections: clinical chemistry and microbiology; parasitology; haematology and blood bank. Some have the fourth component: histopathology lab. (11.7% (3,999) of health facilities)<br>• Tertiary: college of medicine, the university teaching hospitals, where training for healthcare professionals are also conducted. Specialized services, specialized departments, including the labs. (0.2% of health facilities)<br><br>Of the previous 34,176 health facilities, 88.1% (30,109) belong to primary health care while 11.7% (3,999) and 0.2% (68) are secondary and tertiary health facilities respectively<br><br>Three levels of health care :<br><br>Community (community health workers and lab technicians: for Malaria<br><br>Primary (mostly under funding state government, although supposedly under local government authorities; and overseen by federal primary health care development agency). Level of service provision and laboratories differs across PHC centers and states (some have no lab). By standards each PHC centre is supposed to have a laboratory to support malaria treatment (microscope)<br><br>Secondary: general hospitals for general health services delivery. Should have a lab with three sections: clinical chemistry and microbiology; parasitology; haematology and blood bank. Some have the fourth component: histopathology lab.<br><br>Tertiary: college of medicine, the university teaching hospitals, where training for healthcare professionals are also conducted. Specialized services, specialized departments, including the labs
	Distribution of laboratories across public, private for profit / not for profit	Not known for laboratories, but for health facilities: primary: 73% public; secondary: 24% public; tertiary: 88% public (DOC2). However, private facilities provide 60% of the health care services through 30% of the country's health facilities (DOC3) [*same in Kenya – people use private facilities more*]<br><br>DOC 2: Depending on tier:<br>1. Primary level: More public than private (21800 : 8200) Based on the Ward Health System, the three recognised facility types are; (1) Health Post (2) Primary Health Clinic and (3) Primary Health Care Centres. (NPHCDA, 2011 - National Primary Health Care Development Agency).<br>2. Secondary level less public than private (969 :3023)<br>3. Tertiary: More public than private (73:10)<br><br>DOC3:<br><br>Nigeria has a growing private health sector which provides 60% of the health care services through 30% of the country's conventional health facilities – this includes not-for-profit services provided by faith-based and non-governmental organizations; and private-for-profit providers. The broader private health sector also includes traditional medicine providers, patent and proprietary medicine vendors (PPMVs), drug shops and complementary and alternative health practitioners.<br><br>[thus % of structures do not say much. % of users say more]
	Challenges in laboratory system, differentiating for tiers and areas *(problems in availability and*	Many challenges in laboratory system: See also situation analysis in NEDL – survey of 62 laboratories.<br><br>• poor availability of tests for common diseases, other than for programmes malaria, HIV, (DOCs) ; programme supported labs are of higher quality than not supported: *''PEPFAR lab are fully equipped, fully functional, air conditioned, with all the machines, well renovated and all of that. When you step away from that*

	access to – specific - IVDs)	*the general lab is dilapidated, still very manual and all of that.''* – this created a parallel structure (#5)<br>• high costs of tests<br>• no LIMS;<br>• lack of personnel; Lab HR are available in Nigeria, but not absorbed in the public system (#2)<br>• poor amenities;<br>• lack and poor maintenance of equipment;<br>• lack of standardisation of laboratory services;<br>• insufficient funding;<br>• poor linkage between clinical and medical laboratory services [lab services not used by clinicians – example: 2010-2015, percentage of children with symptoms of malaria being tested has risen from 5% to 13% but percentage of children taking ACT for malaria treatment has risen from 12% to 38%. (DOC3)]<br><br>Challenges that are improving:<br>• Clinicians' confidence in and use of lab tests is increasing (#5)<br>• 'Organization, Coordination and Linkages' and 'Quality Management System. (DOC4 – after evaluation previous policy and strategic plan)<br><br>DOC 1: related to availability of tests ; high costs; for common diseases; no LIMS personnel, to amenities, to equipment<br><br>DOC3:<br>• Lack of standardisation of laboratory services<br>• Poor maintenance of lab equipment.<br>• Absence of public health laboratories<br>• Poor linkage between clinical and research laboratory services<br><br>Example of poor lab system: Between 2010 and 2015, the number of children with symptoms of malaria being tested has risen from 5% to 13%. and the percentage of children taking ACT for malaria treatment has risen from 12% to 38%.<br><br>DOC4: Situational analysis end-term evaluation of strategic plan 2015-2019: implementation challenged by a weak plan design, inadequate funding, coordination and absence of monitoring and evaluation framework and plan. Significant achievements were however observed in two (2) critical system thematic areas viz: 'Organization, Coordination and Linkages' and 'Quality Management System."'<br><br>#5: Generally improvement lab quality and status of lab services and confidence of users in lab results – but not in all states and locations. Challenge is the inter-professional rivalry in the system where we have the pathologists wanting to lord over the laboratory scientists; that has been a major issue bringing down service implementation in NIG. However, still programme supported labs are of higher quality than not supported:'' PEPFAR lab are fully equipped, fully functional, air conditioned, with all the machines, well renovated and all of that. When you step away from that the general lab is dilapidated, still very manual and all of that.'' – this created a parallel structure<br><br>#3: NIG adopted the ISO 15.89 standards for public and private labs; some secondary and tertiary labs accredited. There are guidelines for primary tier labs, but they are not supported and infrastructure is poor. For primary level they now implement SLIPTA programme of ASLM (also for secondary)<br><br>#2: There are HR for the lab, but they are not absorbed in the public system. Some private HCs have the capacity to employ lab scientists and technicians
**Stakeholders in lab system and services**	Technical	Many technical partners; Mentioned by key-informants for development NEDL: MSH (with fund Global Fund health system strengthening (GFHSS) program), and WHO employed consultants.<br><br>#5 MSH during Global Fund health system strengthening (GFHSS) program supporting the development of basic document that are required for providing oversight to lab services in

		NIG: revision of the NIG medical lab service policy (finished) and the developing of the strategic plan (finished by consultants but needs a final validation – NLTWG validated, but pathologists hold back)<br>#2: WHO and MSH (with money Global Find) Consultants for development of NEDL
	Implementing	PEPFAR : PEPFAR supported the infrastructure, across the country 2 labs per state were renovated and upgraded to support general lab service<br>#5 PEPFAR supported PEPFAR labs: with equipment, airconditioning, machines, renovation of building etc. GF HSS Grant also supported the infrastructure, across the country 2 labs per state were renovated and upgraded to support general lab service
	Funding	WHO and Global Fund for development NEDL<br>#2: WHO Nigeria and Global Fund, for NEDL: Fees Consultants for development of NEDL; WHO: stakeholder discussion and validation meetings, printing of documents; survey logistics, travel, training
	MoH departments / states / regions	FMOH, States, Primary Health Care Authority – for setting guidelines, procure and supply public labs of different levels in their state<br>States set guidelines, procure and supply public labs of different levels in their state
**National committee/working groups on IVDs**	Committee name(s) and aims	National Laboratory Technical Working group (NLTWG), inaugurated by the Minister of Health. January 27th 2017 – also work on IVDs. An advisory body, meeting every three months, whose mission is to "provide technical guidance for coordination of medical laboratory systems, services and oversight function on the implementation of laboratory policies by the tiers of government and other stakeholders in Nigeria." (DOC4) The federal working group encourages states to establish their own LTWG – 6 states have already been supported.<br>#1: National Laboratory Technical Working group (NLTWG), inaugurated by the honourable Minister of Health. January 27th 2017; aims: coordinate laboratory activities and harmonize activities of different organisations. [#2: MOH decided that it would be good to have stakeholders outside the MOH to help it in terms of issues related to lab services and get their buy-in; 2017 because national policy and strategic plan had to be revised.] Is the federal working group; they encourage states to establish their own LTWG – 6 states have already been supported.<br>How to become a member: organization council of Nigerian pathologist is member and if president not available to attend , he attended. (#4)<br>DOC4: Lab Technical Working group is an advisory body whose mission is to "provide technical guidance for coordination of medical laboratory systems, services and oversight function on the implementation of laboratory policies by the tiers of government and other stakeholders in Nigeria." The NLTWG Secretariat is domiciled within the Division of Medical Laboratory Services currently in the Department of Hospital Services<br>DOC2: Malaria Partners Forum
	Categories of committee members, % women	Committee members are from organisations, total 53 members. Members are amongst others: the National blood service transfusion; national agency for the control of AIDs; national TB program; medical laboratories.; medical and dental council of Nigeria; Institute of Nigeria medical research; professional association of medical and scientists and association of pathologists; Nigerian center for Disease Control; WHO and USAID and all those US Govt partners. Percentage women varies, because organisations are member and send representatives, not individual members based on personal qualifications<br>They are #1: Based on organizations – 53 members. They meet every quarter. Members are amongst others: the National blood service transfusion; national agency for the control of AIDs; national TB program; medical laboratories.; medical and dental council of Nigeria; Institute of Nigeria medical research; professional association of medical and

		scientists and association of pathologists; Nigerian center for Disease Control; WHO and USAID and all those US Govt partners.
	Who decides on the members of these committees and what are criteria for membership	MOH decides: Criteria are organisations, associations, and departments involved in lab domain.
	Missing stakeholders in committees?	Some implementing partners of donor agencies that are not passing through the FMOH lab division – she has seen them when travelling in the states and LGAs. There should be a nation-wide inventory of those organization. A rule should come up that for any intervention coming into the country from anywhere has to pass through the federal ministry and when it passes through the federal ministry you now have to see with your intervention on core component of the strategic plan. That will ensure that all interventions are being coordinated and achieve a scientific goal or vision for the country instead of pockets of interventions here and there that are not coordinated and not giving us a direction or way forward.] (#5)
	If no committee: what the MoH plans/ what are barriers ?	
**Presence of national laboratory documents**	Laboratory policy	Nigeria National Laboratory Services policy 2021-2025 – NEDL is thematic area #9 of the policy
	Strategic plan	The previous national laboratory Strategic plan 2015-2019, written by a core groups appointed by FMOH was not implemented as the plan was not costed and there was no specific Department in the Federal Ministry of Health directly responsible for the implementation.<br><br>Now a draft National Laboratory Strategic Plan and Annual Operational Plan, which is not validated yet and has no budget. Development of the plan and policy part of GFHSS (the lab arm), and technically supported by MSH. The draft is already reviewed by a group of lab experts. -<br><br>Policy implementation process covering a period of five years is to be executed through a National Laboratory Strategic Plan and Annual Operational Plans [draft reviewed version from group of lab experts -no budget, not validated yet]<br><br>National Laboratory Strategic Plan 2015-2019 (process started in 2012, by a core group appointed by the FMOH - no appreciable effort was made to implement the plan as the plan was not costed and there was no specific Department in the Federal Ministry of Health directly responsible for the implementation.
**Presence of documents with guidelines on IVDs**	National documents that address (essential) IVDs (*dates, budget?)*	**NEDL 2021 – no budget**<br><br>**Malaria strategic plan 2014-2020 (has budget)**<br><br>DOC Nigeria National Laboratory Services policy 2021-2025<br><br>#1: They have generic tests on the lists– no specific brands. Needed equipment and consumables to do the tests are not part of the NEDL. Lab policy and strategic plan address tiers of labs<br><br>DOC2: Malaria strategic plan 2014-2020 (with budget for Programme Cost of $348,944,127; Drug, Commodities and Supplies of $3,553,248,668 and Health System Cost for Logistics (Drug & Commodity distribution) of $230,917,375.)
	What vertical (disease) programs identify priority IVDs ?	Malaria programme

		DOC2: Objective 2 is related to laboratory: To test all care-seeking persons with suspected malaria using RDT or microscopy by 2020: Universal access to parasitological confirmation of malaria at all levels. Possible because of RDTs at PHC level.
	Whether documents define (essential) IVDs by tier – if so, which tiers? (including community level)	NEDL: Two tiers: 1. Community and primary health facilities without lab; 2: health facilities with lab. Only the two tiers, because NEDL followed structure WHO EDL (#5)<br>***For Case Nigeria on NEDL*:** Doc 1: Two tiers: 1. Community and health facilities without lab; 2: health facilities with lab. (#5 Only the two tiers, because NEDL followed structure WHO EDL.)<br>IVDs of NEDL: The NEDL enlists 145 diagnostic test categories comprising 65 general IVDs for detection and aid to the diagnosis of a range of disease conditions; 73 disease-specific IVDs in clinical settings covering primary, secondary, tertiary and national reference laboratories and 7 IVDs for screening of blood donations. It also includes 12 general IVDs and 15 disease specific IVDs for use in community and health settings without laboratories. (Diseases: Cholera; Hepatitis B and C; HIV; Malaria; Syphilis; TB; Peptic Ulcer).<br>No brands of IVD, no categorization and standardization by tier of lab system (as they had before). Donors, decision makers, policy makers and users can chose from the NEDL what do we use to manage at a particular area. Lab and medical professionals, managers and policy makers from health facilities decide together what IVDS are needed to tackle the disease burden in the area – referencing the NEDL – and make considerations based on what tests already available in the facility, infrastructure, power supply, water supply, HR. (#2).<br>Malaria strategic plan: RDTs at community level<br>#2: NEDL is a robust list of diagnostics that donors, decision makers, policy makers and users can have access to deciding now what do we use to manage at a particular area. So we didn't want to go like a fixed regulation standardization (as they had before). Lab and medical professionals, managers and policy makers from health facilities decide together what IVDS are needed to tackle the disease burden in the area – referencing the NEDL. What determines what tests are available in that center, infrastructure will play a role, power supply, water supply play a role, HR will play role, the disease burden will play role and all that.<br>#5 Only the two tiers, because NEDL followed structure WHO EDL.<br>DOC2: RDTs at community level. Objective to Build capacity of personnel in public and private health facilities, and at community level for parasitological confirmation of malaria
	If no/not all documents address IVDs by tier: What are the reasons?	
	If no/not all documents address community tier: What are the reasons?	
**Stakeholders, processes and criteria for prioritizing / selecting IVDs**	Category of stakeholders involved in development of documents addressing IVDs	Stakeholders involved in NEDL: all stakeholders in laboratory domain. All members organisations of NLTWG automatically part of NEDL (#5). Pathologists, physicians, IVDs sellers, procurers, regulatory bodies, marketeers, partner non-govt. organisations including WHO, MSH, Global Fund, NIG CDC, Society for Family Health etc. Practitioners, service providers, FMOH departments, government bodies including agencies, like, medical department, National blood transfusion, state ministries of health. Dept of Defence (DoD). Medical Laboratory Science Council of Nigeria (MLSCN) is one of the regulatory bodies that regulates the profession and practice of medical laboratory services and will ensure that only MLSCN approved IVDs, medical laboratory equipment, reagents, chemicals and consumables are allowed to be produced, imported, distributed, stocked,

		marketed and used in medical laboratories in Nigeria. Other regulatory bodies are the medical and dental councils,.<br><br>Majority of participants were men – about one-fifth of participants in stakeholder meetings were women (DOC1).<br><br>#2 At validation meetings: pathologists, physicians, IVDs sellers, procurers, regulatory bodies, marketeers. Partner Organisations and departments: WHO, MSH, Global Fund. Practitioners, service providers, government bodies including agencies, like NIG CDC, medical department, National blood transfusion, state ministries of health.<br><br>#5: She was involved as head labs in providing oversight, reviewing documents. All members organisations of NLTWG automatically part of NEDL. (NIG medical Council, NIG medical Lab science Council, Vet Council, etc). Missing stakeholders: Implementing partners of donor agencies that are not passing through the FMOH lab division.[A rule should come up that for any intervention coming into the country from anywhere has to pass through the federal ministry and when it passes through the federal ministry you now have to see with your intervention on core component of the strategic plan. And that will ensure that whatever interventions that are coming in are being coordinated and achieving a scientific goal or vision for the country instead of pockets of interventions here and there that are not coordinated and giving us a direction or way forward.]<br><br>#3: He is from NLTWG subcommittee on quality management system: about the IVD lab's guidelines and capacity to validate reagents and test kits. (They have gazetted in vitro diagnostic guideline). [see document; these are guidelines how to regulate IVDs, not the actual list of IVDs: Medical Laboratory Science Council of Nigeria: Guidelines for In-Vitro Diagnostics 2018. MLSCN which regulates the profession and practice of medical laboratory science will ensure that only MLSCN approved In – Vitro Diagnostics (medical laboratory equipment, reagents, chemicals and consumables) are allowed to be produced, imported, distributed, stocked, marketed and used in medical laboratories in Nigeria]. Stakeholders in validation meetings representatives from the regulatory agencies, like the medical and dental council, the medical lab science council of Nigeria, the public analyst of Nigeria; non-governmental organizations, the US CDC in Nigeria, DoD, society for family health.<br><br>NOTE: important to have respected persons to engage stakeholders – that are diplomatic and knowledgeable on the topic and lab domain– as Callista was regarded. She was described by MLSD as having played an important role in stakeholder engagement.<br><br>DOC1: Majority men – about one-fifth of participants in stakeholder meeting were women.
	Steps in development of documents (*consultants, drafts, workshops?)*	1. Initiative from minister of health (prof Emeribe who was in the WHO strategic advisory group of experts of in vitro diagnostics 'sold' the idea to develop an NEDL him).<br>2. Get buy-in of NLTWG in a meeting in 2019. (WHO Nigeria who is member of NLTWG made the group aware of the NEDL. Gvt requested WHO for support - #1)<br>3. WHO and MSH assigned consultants – independent of one another, but then worked together as a team. WHO consultant lead (2 and 1 resp – 2 men, one woman)<br>4. Consultants wrote and presented draft tool for a national survey to understand the lab landscape to guide them in developing the EDL for NIG. '' 'to let us understand the landscape of what is there at primary level, secondary level, tertiary level and public health level. We wanted to know what is there and to know what is needed'' [Tool was based on template WHO EDL, 2nd edition]. Questions on: IVDs presently used, priority disease, human resources, equipment, infrastructure; barriers to diagnosis and access IVDs. NLTWG made inputs to drop and add some diseases.<br>5. (After pretesting): Survey in 62 institutions in 6 states, in primary, secondary and tertiary laboratories in Feb 2020. For analysis survey appointed consultant already working for MSH. (had a paper and later electronic tool. Electronic was used for data analysis. Survey was paid by WHO.)<br>6. Consultants made draft NEDL based on IVDs they found in the survey and what should be there. Surprising were: i) type of IVDs in facilities, that were expected not to be used anymore; ii) big variation across states and facilities.

		7. Presentation findings survey and draft draft NEDL to a large group of stakeholders. First 2-3 day meeting with the laboratory experts and after revisions presented next version NEDL to a 3 days meeting, chaired by the chairman of the NLTWG with a larger stakeholder group, including pathologists, clinicians and other bodies. Discussions in breakout groups. Output of the second working meeting: revised NEDL.<br>8. For 2-5 days people had opportunity to read revised NEDL and send comments by email that were integrated in final NEDL .<br>9. NEDL sent to minister of health for signature, who put his signature in June 2021.<br>10. NEDL is printed with funding WHO (#1)<br>11. Although official launch by minister for dissemination has not taken place yet NEDL was already presented to national council on health. [see with implementation]<br><br>Concerning DOC1:<br><br>#2: With second edition WHO EDL 2019 – WHO decided countries should develop their national EDL, Nigeria, Kenya and Bangladesh selected as pilot countries supported by WHO (Only Nigeria took it up) – Prof Emeribe (Lead consultant WHO) was in the WHO strategic advisory group of experts of in vitro diagnostics. Prof Emeribe 'sold' the idea to the minister of health. WHO appointed 2 consultants, MSH had one. [MSH in parallel had the idea to support labs with an NEDL – their TOR being to give technical assistance in developing national documents - #5] Teamwork WHO MSH was smooth. [#5: Request for consultants did not come from MoH, but FMOH coordinated the process. MSH advertised the TOR for consultant – and together with NLWTG committee did selection]<br><br>[#1: process started in 2019 then COVID slowed down the process.<br><br>Second step: Get buy-in of NLTWG [see committees] in a meeting in 2019. [#1: WHO Nigeria who is member of NLTWG made the group aware of the NEDL. Gvt requested WHO for support]<br><br>Consultants presented draft tool for a national survey to understand the lab landscape to guide them in developing the EDL for NIG. Draft tool was based on template WHO EDL, 2nd edition. NLTWG made inputs to drop and add some diseases. In tool questions on: available IVDs that are presently used, priority disease, but also at human resources, equipment, infrastructure; barriers to diagnosis and access IVDs. [also #1]<br><br>After pretesting and pilot and finalization data collectors in six states trained who did the survey in primary, secondary and tertiary laboratories. [#1] Survey in 62 institutions in 6 states in Feb 2020. For analysis survey appointed consultant working for MSH ''to let us understand the landscape of what is there at primary level, secondary level, tertiary level and public health level. We wanted to know what is there and to know what is needed'' [#1: Had a paper and later electronic tool. Electronic was used for data analysis. Survey was paid by WHO.]<br><br>Consultants made draft NEDL based on IVDs they found in the survey. [#1: surprising findings were the type of IVDs that they use in facilities – that you did not expect them to use it anymore and the big variation across states and facilities. They put in NEDL: the IVDS that were already used in the system and what should be there]. They presented and discussed findings survey and draft NEDL to a large group of stakeholders. First meeting was with the larger Lab house and lasted 2 or 3 days. After revisions presented it to a bigger house that included the pathologists, clinicians and other bodies that took 3 days. [#3: Chairman of the NLTWG chairs the meeting].There were discussions on whether some test could be done at community level. [#3: participative process. There was room for discussions after group work breakout sessions; arguments were on what type of staff could do the tests, and at what tier. Pathologists and lab council advocated for quality control by district lab scientist of tests done at primary level by lab technicians and CHEWs] Output of the last working meeting was the revised NEDL – final touches could be done by email.<br><br>#5: Experts in the meeting discussed at what level tests could be done, consider difficulties of test, human resources, expertise, equipment, diseases etc.<br><br>#4 Different meetings, even after two meetings, people still had opportunity for 2-5 days to send comments by email.

<table>
<tr><td></td><td></td><td>With those revisions: sent to minister of health for signature.

#1: NEDL is printed with funding WHO. Minister put his stamp on the NEDL in June, 2021. Official launch for dissemination not taken place yet – was supposed to be before Xmas 2021, but canceled, has to fit in his agenda. However: NEDL was already presented to national council on health. [see with implementation]</td></tr>
<tr><td></td><td>Criteria for selecting essential IVDs (by tier, clinical care or disease surveillance)</td><td>• Conditions with high disease burden/high public health relevance (prone to outbreaks) where diagnostics have a clear impact on the diagnosis and management of a disease
• Tests encompassing care pathways of diseases/conditions.
• Critical supporting tests such as complete blood count (CBC) and C-reactive protein (CRP).
• Tests that enable safe and rational use of National Essential Medicines List
• Available tests in labs (based on survey)
• Affordability (health financing)
• Skills of human resources / health practitioner
• Accessibility
• Infrastructure.
• The types of testing appropriate at each tier of the health care system depending on factors such as access to electricity, reagent-grade water, phlebotomy and specialized human resources

Note by #1: “Most tests are essential but some are more essential than the others ‘’

#1: “Most tests are essential but some are more essential than the others ‘’ Criteria

• Disease burden (and what is needed to manage the disease burden)
• Available tests in labs (based on survey)
• Affordability (health financing)
• Skills of human resources / health practitioner
• Accessibility
• Infrastructure.
• Amenities as water and electricity

DOC1:

• Conditions with high disease burden/high public health relevance where diagnostics have a clear impact on the diagnosis and management of a disease
• Tests that enable safe and rational use of National Essential Medicines List
• Conditions prone to outbreaks/epidemics (SUCH AS?)
• Tests encompassing care pathways of diseases/conditions.
• Tests that are not the mainstay of diagnosis but are critical supporting tests such as complete blood count (CBC) and C-reactive protein (CRP).
• The types of testing appropriate at each tier of the health care system depending on factors such as access to electricity, reagent-grade water, phlebotomy and specialized human resources</td></tr>
<tr><td></td><td>Discussions and (dis)agreements in prioritizing IVDs – by tier (note: RDTs in community?)</td><td>Arguments centered around whether some test could be done at community level, level of staff to do the tests and at what tier (considering difficulties of test, available human resources, expertise, equipment), how quality control should be organized. (Pathologists and lab council advocated for quality control by district lab scientist) Decided on more RDTs in community than in other countries – as long as there could be quality control from higher level. Discussions: ‘’ ’Labs are defending their territories but in terms of access to diagnostics it's good to upscale, for you to upscale you have to have more people doing tests that are not demanding, RDT is all about that.’’ (#2). Other argument about whether to keep tests that are usually done in the labs, but are no longer definitive. Example ESR (erythrocyte sedimentation rate) – they kept it on the NEDL. (#1). Initially it was decided to put tests by lab-tier, but in the end just two: with and without lab.

#2: At level 1, many RDT, more than in other countries, glucose, heamoglobine, urine analysis, cholera, hepatitis, check NEDL] [as long as a laboratorian can do quality control. ‘’Labs are defending their territories but in terms of access to diagnostics it's good to upscale, for you to upscale you have to have more people doing tests that are not</td></tr>
</table>

		demanding, RDT is all about that.'' In Nigeria insufficient human resources, so do task shifting, so better accessibility to tests (Not everyone in lab domain agrees)<br><br>#1: Example of arguments, about ESR (erythrocyte sedimentation rate) add it or remove it entirely because some people just believe that it's no longer definitive. ESR is typically used in many facilities in the country. The consensus was let it be there.<br><br>#4: Some omissions of some terms in the draft. Some tests not tied to the diseases . Not captured well: in histopathology, something with immune chemistry that can be done in some labs (for instance for breast cancer). Thus people on the ground could add some tests that they are using or need. Urinalysis strip can be used for many things, in microbiology, diabetes and has to be put in different places in the NEDL.<br><br>Pregnancy test, malaria tests no need for lab. They took into consideration what the different three levels of labs can do. Initially they wanted to do it by tier. He does not know why it was put together
**Intention and plans to develop NEDL *(processes in development of Nigeria NEDL)***	Knowledge about WHO EDL and perceived usefulness of WHO guidelines for developing NEDL?	Have used EDL 2 for their NEDL and followed format of two levels (with and without lab) and tool for survey of lab landscape. One of the consultants had been in expert committee of WHO EDL.
	MOH's taken and/or planned steps for developing NEDL or similar document (who took the initiative, survey, who writes, stakeholders involved, validation etc)	See above - steps
	Perception of usefulness of NEDL in solving (some) barriers to availability of IVDs in tiers / lab system	NEDL can guide health facilities in prioritizing test and procurement to make test more available to clients.<br><br>#4 Expect that all tiers use the list. Better access to tests for population. List is basis of tests that all health facilities should have – health centres can also get more tests.
	Present and foreseen challenges in development of NEDL	
**Implementation of NEDL**	Plans and steps for implementation	Need official launching by minister of health. However, the NEDL is already known and used by stakeholders who participated in its development, and the FMOH LDirectorate already presented (dec 2021) the NEDL to the national council on health, which is the highest decision and policymaking body on health with all state ministries of health take part. FMOH and State have the right to make their own laws on health issues, but come together to agree on certain things). The council agreed on usefulness of an NEDL. Procurement agencies/departments at federal, state and facility level prioritize request for procurement of IVDs that are on NEDL. Disease programmes (HIV and malaria) also already use the list.<br><br>NEDL is part of new lab strategic plan (that needs validation) and will then be costed. Now no budget.<br><br>For implementation one needs to procure equipment that is linked to the recommended tests. This equipment should be verified and validated at the Public Health IVD Control Laboratory before use (as the specific brands of IVD). There should be training and recruitment of qualified personnel. To reduce cost: capacity should be built in- country

		towards the manufacturing of these IVDs. Support public sector and NGO/FBO laboratories in the provision of multiplex IVDs, improved governance structure, quality and data management, trainings, communication, utility supplies, procurement planning and management to minimize/eradicate stock-outs of IVDs on the NEDL.<br><br>#5 No provision made in budget for implementation of NEDL<br><br>#1: Dissemination: Official launch by minister of Health, but already presented to national council on health in dec 2021 or Jan 2022. They were given printed copies and council agreed usefulness of the NEDL. (FMOH and State have the right to make their own laws on standing health issues, but come together to agree on certain things. Highest decision, policymaking on health is the national council on health).<br><br>Most stakeholders involved in development implementing already. For instance by procurement agencies/departments of federal, state and facility level. When they get a request for procurement can see whether the requested IVD is on the list, and thus approved.<br><br>From the NEDL the policy makers, health practitioners, funders and procurers, at different levels decide what to buy. Funders of specific programmes buy the relevant tests (HIV, Malaria)<br><br>DOC1:<br><br>• FMOH, SMOH and Agencies need to train medical laboratory professionals on ISO 15189 which is the standard that guides medical laboratory services across all levels of care, implement and plan towards quality improvement and accreditation. It should be made mandatory for all laboratories to key into the EQA programme of the National External Quality Laboratory or any other accredited EQA programmes.<br>• Public sector and NGO/FBO laboratories need additional support for the provision of multiplex IVDs, improved governance structure, quality and data management, trainings, communication, utility supplies, procurement planning and management to minimize/eradicate stock-outs.<br>• Procure equipment that is linked to the recommended tests and ensure all medical laboratory equipment and IVDs are verified and validated at the Public Health IVD Control Laboratory before use.<br>• Capacity should be built in- country towards the manufacturing of these IVDs to make access easier and affordable.<br>• Inadequate number of qualified personnel observed in most of the laboratories is not in consonance with the ample numbers of training institutions and qualified Medical Laboratory Scientists and Technicians unemployed and under-employed over several years in the country. There are also inadequate number of Pathologists. This anomaly should be addressed urgently with fresh recruitments and the replacement of unqualified staff rendering medical laboratory investigations.
	Which stakeholders are /will be involved in implementation	Considering that the NEDL is just guidance document, there are other arms for enforcement and regulations. Procurement agencies and manufacturers can hand in their dossiers to supply what is on the NEDL. They submit their products the public health in vitro diagnostic labs that assess IVDS in the country.<br><br>National Assembly for legislation; regulatory bodies for specifying the cadre of human resources per tier and ensure that the various levels of government and private sector involved only employ qualified people and 'get rid of' the unqualified staff who are currently doing tests. Stakeholders to use the list are FMOH and States (commissioners of health), various disease programs who support and fund various lab services. Training institutions for lab personnel.<br><br>#1 : States, facilities, procurement agencies, manufacturers can hand in their dossiers to supply what is on the NEDL. They submit their products the public health in vitro diagnostic labs that assess IVDS in the country (these labs have bene established some

		years ago). EDL list is just guidance document, there are other arms for enforcement and regulations.<br><br>#2 Those involved in lab system can key-in to some of these problems. Stakeholders to do this: FMOH; various disease programs who support and fund various lab services; National Assembly for legislation; regulatory bodies for specifying the cadre of human resources per tier and ensure that the various levels of government and private sector involved get those people who are unqualified get out of the labs. We need quality management system in place (equipment, formation and HR, structure) for successful implementation of NEDL. It will help with national health emergenzies.<br><br>#4: FMOH meets with commissioners of health of states who should get the list adopted and enforced and
	What problems in lab services will/may be solved with successful implementation of NEDL?	High costs and use of many different tests: Reduce the cost of these tests at the various levels when procurement of priority IVDs is coordinated and in bulk. Poor availability of tests: NEDL reduces time of validation by IVD labs because the labs will prioritize IVDs on the NEDL.<br><br>Can reduce the cost of these tests at the various levels when procurement is coordinated.<br><br>#2: NEDL addresses the barriers in the lab system in 5 areas. #3 NEDL can solve problem of uncoordinated supply and procurement and high costs – because if coordination there can be bulk purchase with lowers price. IVD labs will prioritise validation of tests that are on the NEDL and have results in short time.
	(Foreseen/possible) Problems in implementation of NEDL and how to solve these problems	Structure of health and lab system: States (36) can make their own regulations, so they can decide to adopt the NEDL or not. No coordination of procurement. States buy some and most hospitals buy individual needs. There is no regulation that they should restrict buying tests from the NEDL. There are many suppliers marketing different kinds of products to the autonomous facilities and states. There is no regulation. State level procures for public facilities, also for the primary level. Laboratories give requests to procurement depts. Private facilities procure their own.<br><br>#5 Structure of health and lab system: No coordination of procurement. States buy some and most hospitals buy individual needs. There is no regulation that they should restrict buying tests from the NEDL. Now there are many suppliers marketing different kinds of products so that it depend on the individual facility and the things somebody get to market to them. A marketer can come and they are able to sell their products. Regulations difficult to make: States can make their own regulations, so they can decide to adopt the NEDL or not. (36 states) [#1 on procurement: federal level most of the facilities are autonomous and have their own procurement procedure. State level procures for public facilities, also for the primary level. Laboratories give requests to procurement depts. Private facilities procure their own.]<br><br>#4 Not really – the NEDL is a list of tests that are already been done
**Recommendations**	For own country: steps to take in developing and implementing NEDL by tier	Have a laboratory strategic plan with budget – with NEDL being part of it. This will ensure that interventions are being coordinated and contribute to the vision for the country instead of isolated pockets of interventions. Disseminate and popularize and ensure funding from MoH and partners ‘’ *it's not an end point to have a document you have to think about the implementation*.’’ (#5)<br><br>#2: Try to bring NEML and NEDL together because it becomes easier to cost and to convince funders: We need these IVDs for effective health coverage: “"If you make diagnosis available to the people who are concerned, you actually can guarantee, safer use of medicines, in the essential medicine list, more rational use of medicines.”” (<br><br>#5 Have a strategic plan with budget – with NEDL being part of it. That will ensure that whatever interventions come in are being coordinated and achieving a scientific goal or vision for the country instead of pockets of interventions here and there that are not coordinated and giving us a direction or way forward. . Disseminate and popularize and get funding from MoH and partners ‘’ it's not an end point to have a document you have to think about the implementation.’’

		#2: Try to bring EML and EDL together it becomes easier to cost, to say it cost this or this dollars at this level for effective health coverage
	For other countries: steps to take in developing NEDL by tier *(Nigeria's lessons)*	Motivate the minister of health of the importance to have an NEDL. The minister has the capacity and the responsibility to source for funding from development partners. Then move down to medical directors in MoH, then lab leadership and professionals. Let them understand how the UHC benefits from an NEDL.<br><br>Budget for a survey of lab landscape – do not skip it, because the situation is already known. Even if the challenges are already known – the survey data brings the situation including challenges to the national consciousness of all stakeholders in the process and is basis for the NEDL.<br><br>Appoint able laboratory scientists who are knowledgeable on IVDs as consultants who are respected in the country to lead the process: the landscape survey, the writing of drafts and final NEDL. Consultants have more time than FMOH personal and have to keep to set deadlines.<br><br>Engage all stakeholders in the laboratory domain in development, so they feel they own the NEDL - these include medical and other actors in the medical setting. That makes all aware and implementation easy. '' because none of us will be seeing the document for the first time we were actually involved in the development''<br><br>Ensure funding – which will be easier if minister is motivated and NEDL is popularized in MoH and partners: Need funding for consultants, survey, stakeholder meetings, printing, but also plan and budget for revision and implementation.<br><br>Align NEDL with national health care delivery structure; look at where are lab services situated in MoH, what is functioning and status of health and lab services. Do not just adopt the WHO EDL structure, with adjustments, but differentiate by primary, secondary and tertiary tier (and tier without lab), '<br><br>#2. Do not skip the survey for reasons that you already know the challenges and landscape and do not have to spend money on this. Indeed, in our survey we did not find new challenges, but in this way they were brought to national consciousness for the purpose of planning for the NEDL, policy and strategic plan. Appoint able consultants; Advise: WHO list, should be adopted, adapted, modified and owned up by the various countries.<br><br>#3 Engage all stakeholders in development as we did, so they feel they own the NEDL - these include medical and ither actors in the medical setting. That makes all aware and implementation easy. '' implementation will be very easy because none of us will be seeing the document for the first time we were actually involved in the development'' Select very able laboratory scientists who are knowledgeable on IVDs as consultants who are respected in the country. Better use consultants, because they streamline and are given timelines they have to keep. Ensure funding.<br><br>#5 Motivate the minister of the importance to have an NEDL, then move down to medical directors in MoH, then lab leadership and professionals. Let them understand how they benefit from it, and the UHC. Then popularize and get funding from MoH and partners '' it's not an end point to have a document you have to think about the implementation.''. Align NEDL with own health care delivery structure; look at where are lab services situated in MoH, what is functioning and status of health and lab services. Do not just adopt the WHO EDL structure, with adjustments. Differentiate by primary, secondary and tertiary tier (and tier without lab), '' Domiciled the NEDL within the national healthcare delivery system''. Plan and budget for implementation and revision – [we have not done that]<br><br>#1 Have high level commitment from the minister of health who has the capacity and the responsibility to source for funding from development partners. Involve all relevant stakeholders in policy making and implementation in the process. Tailor the NEDL to your country context, considering health service delivery and lab system – not just copy form other countries. Have an expert committee develop a draft and present to a broader spectrum of stakeholders. Source funding for implementation and regular revision.

		#4 Look at human resources. Can also be done by assembling professional bodies in laboratory at different levels of labs. Meet at basis of diseases, equipment, human resources. Using consultants is easy. Same result.
	For development of NEDL: considering gender of recipients	
	For ASLM / FIND/ WHO: type of support for countries in development and implementation of NEDL	• Advocate with MoH for NEDL<br>• Technical assistance in development<br>• Look for funders. They should be interested, because it advances the UHC<br>• Get funding for a few pilot countries and then upscale: *'No African country will have any dollar tied up to developing EDL for themselves''* (#2)<br><br>#5 Support with technical know-how and mobilize for funding of 1) development (consultants, surveys (travel), stakeholders meetings at various levels, printing the document); 2) implementation; 3) M&E and revision.<br><br>#2: 1) Advocate to member states MOH for the NEDL; 2) operationalize, i.e. seed money to develop NEDL in a few countries and then upscale. ''no African country will have any dollar tied up to developing EDL for themselves''.<br><br>#3 Look for funders – who should be interested, because it advances the UHC. “"If you make diagnosis available to the people who are concerned, you actually can guarantee, safer use of medicines, in the essential medicine list, more rational use of medicines.”<br><br>#4 Technical assistance for development. Support implementation , work through FMOH

INTERVIEW PARTICIPANTS FOR COUNTRY: UGANDA

# INT	Background participants
**1**	Laboratory Services MoH
**1**	Laboratory Services MoH
**1**	Laboratory Services MoH
**1**	Laboratory Services MoH
**1**	Partner
**2**	Professional Association
**3**	Partner

DOCUMENTS THAT ARE ANALYSED

# DOC	Name document, year
**1**	Standard test menu, techniques and list of supplies for health laboratories in Uganda (3rd edition, 2017-2020)

FINDINGS BY THEME

Global themes	Specific themes	Findings/Answers (*Refer to # INT and # DOC*)
**MoH organisation**	Position laboratories in MoH *(separate department or sub-division?)*	**UNHLS is a department of MoH. UNHLS is divided into two branches: (1) Coordination arm and (2) Reference laboratories**<br>#1: Department of UNHLS is the technical arm of MOH for laboratories.<br>UNHLS is divided into two branches: (1) Coordination arm and (2) Reference laboratories.<br>Coordination arm include: QA, SS Equipment, Biosafety, Sample Transport Network, Policy, Private Labs<br>Reference laboratories include: Genomic Labs, HIV, VL, EID, HPV, NMRL, NTRL:SRL, Biosafety/Biosecurity, Cancer (INT 1)
**Laboratory system**	Tiers in laboratory system	**Five tiers in laboratory system. There are hubs in levels 2, 3 and 4**.<br>#1:<br>Level 1: H/C III labs<br>Level 2: H/C IV labs<br>Level 3: general hospital labs<br>Level 4: regional referral labs<br>Level 5: national labs<br>Hubs: in levels 2, 3 and 4<br>Now for us to build the capacity in those labs all, it was a bit expensive. So what they did was to make a what we call hubs. A hub is a facility that can serve a radius of 40 kilometers. Now we established 400 hubs and we build the capacity in those hubs. A hub can be at this level, it serves about 30 to 40 facilities irrespective of the level
	Laboratory tiers related to tiers health system (*including community level*)	**Five level in the health system.**<br>#1:<br>6 National Labs<br>16 Regional labs<br>164 General Hospital Labs<br>196 H/C IV Labs<br>1290 H/C III Labs
	Distribution of laboratories across public, private for profit / not for profit	Not asked

	Challenges in laboratory system, differentiating for tiers and areas *(problems in availability and access to – specific - IVDs)*	**Many challenges with infrastructure (tight spaces), irregularity of supply and stock-outs, regulation of test coming in the country.**<br>#2: Lack of laboratory support for microbiology and microbiological resistance studies<br>Lack of infrastructure<br>#3: Irregularity of supply and stock-outs<br>#2: IVD regulations defining the type of diagnostics that enter the country or are used in laboratories is one of the biggest challenges<br>"there is so much equipment coming in and we find that they have a problem and especially RDTs"<br>The rooms used are not up to standard. The tests are done by nurses and other health workers and they don't use lab staff. So they will never register this lab because when they come, they say we don't have a lab<br>#3: Tight spaces in laboratories
**Stakeholders in lab system and services**	Technical	#1: PEPFAR, CDC, USAID, DOD, WHO, GLOBAL FUND, UKAID
	Implementing	#1: USAID, CDC, DOD, WATER REED PROJECT, TAS<br>Funding source determines what gets covered
	Funding	#1: PEPFAR, CDC, USAID, DOD, WHO, GLOBAL FUND, UKAID<br>Fund and technical support
	MoH departments / states / regions	**General directions (Public health, clinical services, planning), Permanente secretary, Civil society, National association of lab workers, Medical bureaus.**<br>#1: Minister<br>General Directions<br>Permanente secretary<br>Directors (Public health, clinical services, planning)<br>Civil societies (CSO, NGO)<br>- Advocacy for funding<br>- Engage communities<br>- Demand creation for lab test<br>National association of Lab workers<br>- Populating guide and politics<br>- Advocacy for adopting guides and politics<br>Medical bureaus (catholic, protestant, muslim)<br>- Coordinate medical services under faith-based organization
**National committee/working groups on IVDs**	Committee name(s) and aims	**(1) Quantification procurement unit, not specific to laboratories but manage quantification in MoH. (2) Lab's supply chain technical committee**<br><br>#1 : There is the quantification procurement unit within the Ministry of Health. This is the largest unit in the Ministry of Health that coordinates all the quantification: HIV commodities, TB, laboratories, and many others.<br><br>So if the laboratory has to organize the quantification, we involve them all. They come in, we make assumptions, and then we quantify. Then we look at what funding sources we have in the country. Then from there, after we look at the funding sources in the country, we say, this is what we have, this is the gap.<br><br>2. There's the lab's supply chain technical committee that meets every month<br><br>If there's a change because suppliers are having trouble, we've discussed it every month and they say suppliers are supposed to bring in commodities, but the supplier manufacturer is having trouble, what do we do?<br><br>They are chair family planning products, there is chair for HIV, Chair for lab

	Categories of committee members, % women	Not asked
	Who decides on the members of these committees and what are criteria for membership	Not asked
	Missing stakeholders in committees?	Not asked
	If no committee: what the MoH plans/ what are barriers ?	
**Presence of national laboratory documents**	Laboratory policy (*Look at date*)	Uganda national health laboratory services policy (V 2009; V2017)<br>National lab hub operations guidelines (2016)
	Strategic plan<br>*(Look at date, budget?)*	The National Laboratory Strategic Plan, 2021 to 2025<br>Budget not provided
**Presence of documents with guidelines on IVDs**	National documents that address (essential) IVDs (*dates, budget?)*	**Standard test menu techniques and list of supplies for health laboratory services in Uganda**, (3rd edition, 2017-2020)
	What vertical (disease) programs identify priority IVDs ?	TB, Malaria, HIV
	Whether documents define (essential) IVDs by tier – if so, which tiers? (including community level)	**Tests are defined in all tiers. In community level, basic tests in hematology, parasitology, serology, blood grouping, urine chemistry and immunology.**<br>Doc 1: Health Centre III<br>Basic tests in hematology, parasitology, serology, blood grouping, urine chemistry and immunology<br>Health Centre IV<br>Selected clinical chemistry tests, hematology, Prothrombin Test (PT) and blood compatibility testing in addition to all the tests done at HC IIIs. CSF, HVS and swab analysis shall also be performed at this level.<br>General Hospital Laboratories<br>Specialized clinical chemistry tests that include lipid, cardiac profile, thyroid functional tests and fertility hormones, plasmin inhibitors, pancreatic and infectious disease tests in addition to all tests provided at HC IVs.<br>Regional Referral Hospital Laboratories (RRH)<br>More specialized tests such as glycated Hb, Reticulocyte count, Hb electrophoresis, body fluids and swab analysis by performing microbiology culture and sensitivity; mycology and cytology (PAP smear and Biopsy), blood gas, and tumor markers tests.<br>National Referral Hospitals<br>Specialized tests in cardiac profile, blood gas tests, metabolic tests, thyroid functional tests, fertility tests and tumor markers, Hb electrophoresis, coagulation

		tests and plasmin inhibitors. They shall also be referral centres for histology and mycology tests particularly culture and sensitivity.<br>Laboratory Hubs<br>CD4, TB, chemistry and hematology test, in addition to providing services expected at its level of health care.<br>EID, Viral Load, microbiology, histopathology, and outbreak investigations are sent to national reference laboratories.
	If no/not all documents address IVDs by tier: What are the reasons?	
	If no/not all documents address community tier: What are the reasons?	
**Stakeholders, processes and criteria for prioritizing / selecting IVDs**	Category of stakeholders involved in development of documents addressing IVDs	**Top management of MOH, the lab team, the clinicians, the suppliers. Important to include clinicians because they ask for tests.**<br>#1: Top management of MOH, the lab team, the clinicians, the suppliers, representatives to show them the performance of the equipment in the country, some of them.<br>So we involve the many stakeholders especially the clinicians had a great important in this because they are the one who are requesting for tests
	Steps in development of documents (*consultants, drafts, workshops?)*	**National assessment to look equipment first. Then workshop with all the clinicians from the different levels with the management of the Ministry of Health and the national stores.**<br>#1: Before we harmonized the equipment in the country, we did an assessment and looked at the type of equipment we had in the country.<br>So we worked with the management of the Ministry of Health and the national stores.<br>So we had to convince the management of the Ministry of Health that the country had to go through harmonization and reduce the number of products in the supply chain.<br>Now, the senior management has brought the idea. And we agreed on the type of tests to be done at each level, based on the minimum health package of the country<br>To identify priority tests with the help of clinicians<br>We organized a workshop with all the clinicians from the different levels who came to present<br>Now, what test, what method could we apply to each of the levels? For that, we needed the technical team in the lab.
	Criteria for selecting essential IVDs (by tier, clinical care or disease surveillance)	**Criteria based on the minimum health package of the country. Three categories called VEN (Vital, Essential and Necessary).**<br>#1: based on the minimum health package of the country<br>We went ahead and said this is what we call VEN (Vital, Essential and Necessary)<br>Now, if the procurement agents. National stores get money, they will focus on V. If they have more money, they can go to Essential. If they have more money, they have to go to Non-essential or Necessary<br>This was done by the lab technicians with the clinicians
	Discussions and (dis)agreements in prioritizing IVDs – by	

	tier (*note: RDTs in community?)*	
**Intention and plans to develop NEDL** ***(processes in development of Nigeria NEDL)***	Knowledge about WHO EDL and perceived usefulness of WHO guidelines for developing NEDL?	**The document was developed before first WHO EDL. UNHLS plan to use it for revision of their document regarding the specificities of Uganda (disease burden, equipment available).**<br>#1: The EDL is from 2018. Our document was developed before 2018. So we didn't use it.<br>Plans to use it for the revision<br>There is a technical person from WHO who will be involved with<br>But we don't just rely on WHO as a country because we have a different disease burden, different from what WHO does<br>Secondly, we may also have equipment available in the country, which is not WHO, but is good and we put it on the list, even if it is not WHO we can find it.<br>But the WHO prequalification has also been restricted, targeting mainly HIV.
	MOH's taken and/or planned steps for developing NEDL or similar document (who took the initiative, survey, who writes, stakeholders involved, validation etc)	**Any plan for development of NEDL. But plan to review their document considering WHO EDL.**<br><br>I think at the time of our review, because you see at 2020, it's due for review, you must reference or benchmark on that WHO as we do our country specific NEDL.
	Perception of usefulness of NEDL in solving (some) barriers to availability of IVDs in tiers / lab system	**Help countries diagnostise their specific needs.**<br>I think that's the question of how countries have to do or try to align, but they may have others depending on their specific needs.<br>So the burden of disease is also a function of what is available in countries.<br>I think it's about trying to align with the WHO guidelines but also having a country, that's why we have national EDLs.<br>What is interesting is that the WHO provides guidance on how to establish the national EDL. It is not a plan that everyone has to follow,<br>but it's a guide on how to make sure that we have it,<br>Yes, because this essential advice, this guidance, the format of it has to be different from the alignment document.
	Present and foreseen challenges in development of NEDL	**Some labs don't have water. There are many lists to use: PEPFAR list, WHO list, global fund list.**<br><br>#1: I mean in some countries, at level 3, some labs don't have water for example, so some tests can't be done.<br><br>We have the PEPFAR list, we have the WHO, we have the global fund.
**Implementation of NEDL**	Plans and steps for implementation	Any
	Which stakeholders are /will be involved in implementation	Not asked
	What problems in lab services will/may be solved with successful implementation of NEDL?	Not asked
	(Foreseen/possible) Problems in implementation of	**Be sure the equipment is harmonized to bring it to public sector.**<br>#1: The only thing about harmonization is that if the equipment is not harmonized, you can't bring it into the public sector.

	NEDL and how to solve these problems	
**Recommendations**	For own country: steps to take in developing and implementing NEDL by tier	**Use the policy makers to do the harmonization. Involve the big players in the WHO and others before you do harmonization. Involve the top leadership of the Ministry of Health. Have a look at the countries that have already developed a NEDL.**<br><br>#1: You have to use the policy makers to do the harmonization.<br><br>You have to involve the big players in the WHO and others before you do harmonization.<br><br>You have to involve the top leadership of the Ministry of Health and explain to them that they need harmonization.<br><br>You have to involve the national stores and explain to them what they are experiencing.<br><br>If you have a lot of equipment in the system, the national stores are not able to get supplies and the national stores are part of the harmonization. And they explain to management the difficulties they are experiencing. When you have so much equipment on board. And then you have to build a rapport with the top management. So they understand why harmonization is important.<br><br>#3: Consider country specifics<br><br>Now, in WHO programming, the way they provide the guidelines is general, but they are country specific, now you also have to let the country specifics fit into the standards, that's very important.<br><br>Inter-training by involving countries that are already well advanced in the process to share their experience
	For other countries: steps to take in developing NEDL by tier *(Nigeria's lessons)*	
	For development of NEDL: considering gender of recipients	Not asked
	For ASLM / FIND/ WHO: type of support for countries in development and implementation of NEDL	Not asked

INTERVIEW PARTICIPANTS FOR COUNTRY: **ZIMBABWE**

**PLACE OF WORK PARTICIPANTS**: Laboratory Services MOH (all 3); One group interview

DOCUMENTS THAT ARE ANALYSED

# DOC	Name document, year
**1**	UMARU, F.A. 2015. Laboratory Harmonization and Standardization in Zimbabwe: A Framework for improving the quality of diagnostics services through standardization of tiered network, tests techniques, methods, instruments and human resources in Zimbabwe.
**2**	National Health Laboratory Strategic Plan (DRAFT2) 2022-2026 (FINAL NOW?) Ministry of Health and Child Care is committed to strengthening the coordination and quality of Laboratory services to support the implementation of the National Health Strategy. Consultative process

FINDINGS BY THEME

Global themes	Specific themes	Findings/Answers (*Refer to # INT and # DOC*)
**MoH organisation**	Position laboratories in MoH *(separate department or sub-division?)*	Directorate of lab services (DLS) is under Department of Curative Services in Ministry of Health and Child Care. DLS has recently a Logistics Sub-Unit (LSU) responsible for forecasting, quantification, procurement and distribution of all commodities (Laboratory Equipment and Reagents/ Consumables).<br><br>Directorate of lab services under department of curative services from Ministry of Health and Child Care<br><br>a Logistics Sub-Unit (LSU) has substantially improved coordination, procurement and supply management of laboratory commodities. This unit, which is overseen by a Laboratory Logistics Officer, is responsible for forecasting, quantification, procurement and distribution of all commodities (Laboratory Equipment and Reagents/ Consumables).<br><br>Two arms:<br>1. Public health laboratories that serve the Preventive Services Division in the Ministry of Health and Child Care (MOHCC). These include institutions such as the Government Analyst and the (National Institute of Health Research) NIHR. They are community focused<br>2. Diagnostic laboratory service based in hospital institutions, independent private laboratory facilities and the National Blood Service of Zimbabwe (NBSZ). The diagnostic laboratory services focus on patient clinical quality care. These laboratories include public health care laboratories, mission laboratories, uniformed forces laboratories, local authorities, city council laboratories, national reference laboratories [National Microbiology Reference Laboratory (NMRL), National Tuberculosis Reference Laboratory (NTBRL), National Virology Laboratory, (NVL)], private clinical laboratories, veterinary laboratories, research laboratories, training institutions laboratories and Zimbabwe National Quality Assurance Programme,(ZINQAP), that was established in 1998 with the mandate to assist all laboratories in Zimbabwe attain and maintain a high standard of performance and to improve the quality of testing services.
**Laboratory system**	Tiers in laboratory system	Five tiers: 1) National reference labs (for Microbiology; Tuberculosis ; Virology), 2) central hospital labs, 3) provincial hospital labs, 4) district/mission hospital labs, 5) rural health centre labs. RDTs in community (Provided by health cengre with lab). Integrated sample transportation system, where samples that are taken at lower levels move to higher levels, and not patients [This increases access].<br><br>DOCs Five tiers: National reference labs [INT: HIV, TB, and for other viruses in the national biology reference lab], central hospital labs, provincial hospital labs, district/mission hospital labs, rural health centre labs<br><br>over 1700 registered diagnostic centers/ Laboratories, including private laboratories, operating at five main levels organized along the referral chain of health service delivery

		Information on supply issues, stock, is gathered in Lab logistics unit through the routine reports from each of these labs. Also data are collected through routine programme site and support supervision like for the HIV programme and from the national microbiology reference lab there are national teams going out into these facilities and generate a report what's the issue on the ground. Historically supervision visits were vertical (HIV, malaria, TB) – nowadays efforts to include all activities and tests, machines etc,
	Laboratory tiers related to tiers health system (*including community level*)	Laboratories are organized along the referral chain of health service delivery; however some rural and urban health clinics do not have labs on site. Community level: HIV and malaria tests – and COVID tests - are task-shifted to the community (community health workers) and rural or urban health (Nurses, primary counsellors) without a lab on site. Distribution of health products including IVDs through directorate of pharmacy services (DPS)<br><br>See above, but some rural and urban health clinics do not have labs on site.<br><br>HIV and malaria tests are task-shifted to the community and rural or urban health clinics without a lab [and lab personnel] on site. Tests are done by nurses, primary counsellors (who do most of the rapid HIV testing within the country), and community health workers. Sometimes they also do rapid malaria testing and syphilis. And nowadays: COVID. These items used by clinics and community workers are being distributed by the health centres with a lab. In ZIM: integrated sample transportation system, where samples that are taken at lower levels move to higher levels, and not patients. This increases access. (DOC)
	Distribution of laboratories across public, private for profit / not for profit	No information
	Challenges in laboratory system, differentiating for tiers and areas *(problems in availability and access to – specific - IVDs)*	• Lab services structure: Health facility laboratories fall under department of clinical services. Health facility labs report to provincial medical director. This has raised complications in reporting, significantly compromising DLS ability to manage and coordinate the services in the country. Limited staff at DLS level.<br>• Inadequate supplies, stock-outs of tests and poor referral services for other than donor supported programmes (HIV, TB, infant HIV, malaria COVID-19). Poor funding.<br>• No national coordination in procurement (hospitals do their own). However, recent logistic unit at DLS should improve national procurement, supply and monitoring.<br>• Inadequately maintained equipment causing defect machines. Laboratory stakeholders have not been sensitized on the Harmonization and Standardization Guidelines (DOC2), and purchase equipment with varying specifications, leading to difficulties in their servicing and maintenance as well as low utilization.<br>• HR issues with lack of qualified staff to perform the tests at certain level.<br>• Operating with limited budget<br><br>DOC 2: The health laboratory services are geared towards clinical care and are based in health facilities which falls under the department of clinical services. This has raised complications in reporting, significantly compromising DLS ability to manage and coordinate the services in the country. Linkages between the laboratories at different levels remain weak because of lack of focal persons at District level. The DLS has limited HR to oversee over 1700 centres/ laboratories in the country which severely limits top supervision and technical support to these lower facilities. This also overwhelms the limited staffing at DLS level.<br><br>DOC 1: the Logistic unit has greatly improved the supplies situation in the country, the list of supplies is usually HIV related and as a result other tests have stock outs. Only donor supported laboratory services run well, including functional referral systems (for HIV, TB, infant HIV, COVID-19).<br><br>Other problems identified in labs with personnel, reagents, infrastructure etc.

		INT: problem is only ref labs report directly to directorate of lab services (DLS). All other tiers labs through provincial medical director (PMD). However now, with DLS logistic unit – they are also supposed to report to DLS. What to procure should now go through DLS, even if hospitals also had some channels to procure themselves items that are not well funded nationally – logistic unit advises what to procure. DLS may identify the national needs, but these are not 100% funded from donors or government. Distribution of health products including IVDs through directorate of pharmacy services (DPS)<br><br>Unavailability of reagents or defect machines – mainly due to funding issues. Also HR issues with qualified staff able to perform the tests at certain level. Funding gaps in delivery from partners or domestic funding not covering the demand. This results in reagent shortages. Problems with maintenance of machines because needed resources for repair by suppliers. Nowadays better: most of the equipment have a service level agreement where the servicing is part and parcel of the reagent cost such that you don't have a lot of time between a machine breaking down and it being repaired.<br><br>Insufficient lab staff employed by gvt. And difficult to retain staff – salaries are low.<br><br>DOC2: recent efforts have gone into improving disease specific control programs such as HIV/AIDS, TB, and malaria, where the funding has been available through global health initiatives. Challenges are operating with a limited budget, inadequately staffed laboratory facilities, inadequately maintained equipment, and inadequate supplies. Many laboratory stakeholders have not been sensitized on the Harmonization and Standardization Guidelines. As such, many partners purchase equipment with varying specifications, placing them in places whose machine through put is not aligned to expected demographic demand leading to difficulties in their servicing and maintenance as well as low utilization.
**Stakeholders in lab system and services**	Technical	See development harmonization document
	Implementing	See development harmonization document
	Funding	See development harmonization document
	MoH departments / states / regions	See development harmonization document
**National committee/working groups on IVDs**	Committee name(s) and aims	Not available -
	Categories of committee members, % women	NA
	Who decides on the members of these committees and what are criteria for membership	NA
	Missing stakeholders in committees?	
	If no committee: what the MoH plans/ what are barriers ?	
**Presence of national laboratory documents**	Laboratory policy (*Look at date*)	National Health Laboratory Policy and National Laboratory Strategic Plan (NLSP, 2010 – 2014) (In line with Maputo Declaration)
	Strategic plan	National Health Laboratory Strategic Plan (DRAFT2) 2022-2026 (FINAL NOW?)

	(Look at date, budget?)	
**Presence of documents with guidelines on IVDs**	National documents that address (essential) IVDs (*dates, budget?)*	*Most relevant*: Laboratory Harmonization and Standardization in Zimbabwe – 2015 *= sort of NEDL because guiding equipment selection, procurement and deployment to the different tiers of laboratory services. Developed by DLS with support from USAID. Was supposed to be updated and revised every few year*
	What vertical (disease) programs identify priority IVDs ?	HIV, Malaria, TB (but these documents not analysed in QUAL
	Whether documents define (essential) IVDs by tier – if so, which tiers? (including community level)	Yes – but not community level. However, malaria strategic plan (not analysed) and information from interviews show that IVD – RDTs are task shifted to community level.
	If no/not all documents address IVDs by tier: What are the reasons?	
	If no/not all documents address community tier: What are the reasons?	
**Stakeholders, processes and criteria for prioritizing / selecting IVDs**	Category of stakeholders involved in development of documents addressing IVDs	National programmes, departments of MoH, reference laboratories, Central, Provincial and District Hospital Laboratories, provincial scientists, scientific councils, financial, technical and implementing partners (WHO, PEPFAR, BRTI, IDDS, CHAI, USAID)<br><br>Different sections of DLS, provincial scientists; national programmes, such as for HIV, malaria, TB; medical lab scientists council; national institute of health research (part of MoH); partners that support lab services: Clinton health access initiative (CHAI), USAID, BRTI, (Biomedical Research and Training Institution); IDBS mainly for TB, WHO, Zimbabwe national quality assurance programme, (Strategic plan)<br><br>DOC1: National Reference Laboratories, Central, Provincial and District Hospital Laboratories; PEPFAR CDC support through BRTI and IDDS for the financial and WHO technical support throughout the development, drafting, finalization and printing of the plan; members of the Laboratory Community for their commitment and dedication in developing the strategic plan; Partners such as CHAI, FHI 360
	Steps in development of documents (*consultants, drafts, workshops?)*	Strategic plan: Draft written by DLS after landscape analysis based on routine reports by labs submitted to logistics unit of DLS and disease programmes' supervision and monitoring reports; input key public and private stakeholders; desk review; technical support by consultant under leadership DLS. Draft NSP presented to stakeholders for final review and endorsement. Inputs were incorporated to produce a final document.<br><br>Standardization document: Two meetings brought together relevant laboratory services partners, clinicians, procurement agents, care and treatment programs and other stakeholders with specific diagnostic interests.<br><br>• Aims of first 3-days'meeting with mainly clinicians and laboratory professionals: review the current tests offered by DLS, evaluate testing gap, understand long term treatments shifts, establish tiered level network, and identify opportunities and challenges for implementation. Agreed on five tiered testing levels: considering human resources, testing techniques complexity and availability of

		specialist medical practitioners. Outcome of this meeting: list of recommended tests stratified by tier and categorized by priorities (must have, should have, may have (Table 1).<br>• Second 5-days’meeting consultative technical evaluation meeting aimed at reviewing the recommended test menus, standardizing test menus, develop methods and techniques for each test, and outline instrument specification as well as human resource requirements. Participants were laboratory scientists and experts in the different areas of diagnostics services. Objective was also: harmonize list of reagents for the standard test menus and develop implementation plan.<br><br>Overall, sixty priority tests were confirmed as national test menus (Table 4) to be conducted across laboratories. Tests are categorized by tiered network on the basis of methods, techniques, instrument complexities as well as human resource skills. Standardization of instruments to guide on appropriate procurement (and donations) and deployment of new and/or existing instruments.<br><br>Information on lab landscape for strategic plan was from routine reports by labs submitted to logistics unit of DLS and by national supervision and monitoring reports by disease programmes.<br><br>DOC2 The process of developing the NHLSP (2022-2026) was a consultative one that incorporated input from key stakeholders (public and private) both directly and virtually so as to guarantee ownership (see stakeholders). The process also involved desk review of relevant documents including WHO guidelines, national program documents, research studies and relevant international standards. This was coupled with situational analysis to inform the whole process with technical support from consultants and leadership from the MoHCC Department of Laboratory Services. A draft NSP was presented to stakeholders for final review and endorsement. Inputs were then incorporated to produce a final document. Some of the key documents that were used to direct the development of the plan were: Vision 2030 document (2018), TSP (2018-2020), NDS1 (2021 – 2025), Mid-term review report of the NHS (2016-2020), Guidance for Development of National Laboratory Strategic Plans and Strategic framework for strengthening health laboratory services<br><br>DOC1: Two meetings held brought together relevant laboratory services partners, clinicians, procurement agents, care and treatment programs and other relevant stakeholders with specific diagnostic interests. The meeting was to review the current tests offered by DLS, evaluate testing gap, understand long term treatments shifts, establish tiered level network, and identify opportunities and challenges for implementation. agreed on five tiered testing levels considering human resources, testing techniques complexity and availability of specialist medical practitioners. The outcome of this meeting was a list of recommended tests stratified by levels and categorized by priorities (Table 1).<br><br>Following the consultative stakeholders meeting, the DLS further organized consultative technical evaluation meeting with the aim of reviewing the recommended test menus from the stakeholders. This meeting was held at the Caribbean Bay Hotel in Kariba between 23rd and 27th February, 2015. Participants were seasoned laboratory experts in the different areas of diagnostics services with the aim of standardizing test menus, develop methods and techniques for each test, and outline instrument specification as well as human resource requirements. Specific objectives of the technical review workshop were: (i) harmonize list of essential testing algorithm, (ii) establish standard list of instruments to support required testing algorithm, (iii) recommend minimum human resources for each tiered level, (iv) harmonize list of reagents for the standard test menus and (v) develop implementation plan.<br><br>Overall, sixty priority tests were confirmed as national test menus (Table 4), which are to be conducted across laboratories. These are essential tests that can respond to the programmatic and demographic burden of disease in the country and as such, must be available at all times. Additionally, these tests are categorized by tiered network on the basis of methods, techniques, instrument complexities as well as human resource skills.

		Standardization of instruments in order to guide donors, procurement partners and other stakeholders on appropriate procurement and deployment of instruments. Hence, the DLS developed instrument specifications and evaluation criteria that must guide donations and procurements of new and/or existing instruments.
	Criteria for selecting essential IVDs (by tier, clinical care or disease surveillance)	• Programmes present<br>• disease burden<br>• appropriate methods and techniques (by tier)<br>• test / instrument complexities (for the tier)<br>• human resource skills by tier.<br><br>Central/Province/District/Rural health centres: A menu of tests for each level of services has been defined with reference laboratories offering reference services. Central hospital laboratories offer a greater range of more complex tests than provincial and district laboratories. At the lowest level the health centres offer low complexity testing (e.g. rapid HIV tests, malaria rapid diagnostic tests (RDTs), TB smear microscopy and other point of care tests).<br>DOC1:<br>• programmatic<br>• disease burden<br>• methods,<br>• techniques,<br>• test / instrument complexities At the lowest level the health centres offer low complexity testing (e.g. rapid HIV tests, malaria rapid diagnostic tests (RDTs), TB smear microscopy and other point of care tests).<br>• human resource skills.
	Discussions and (dis)agreements in prioritizing IVDs – by tier (*note: RDTs in community?)*	No answers (not asked)
**Intention and plans to develop NEDL *(processes in development of Nigeria NEDL)***	Knowledge about WHO EDL and perceived usefulness of WHO guidelines for developing NEDL?	DLS team to review harmonization document has heard about it, but no read.<br>Have heard about it but have not read (‘’ít is on my reading table’’)
	MOH’s taken and/or planned steps for developing NEDL or similar document (who took the initiative, survey, who writes, stakeholders involved, validation etc)	DLS intends to update the harmonisation document (which is like an NEDL) because there are changes in the system, with more RDTs, new diseases (COVID-19) and tests can be done at lower levels. Revision is in the DLS 2022 workplan. They will involve all technical and funding partners who all have lab focal persons who are lab scientists and the provincial and health facility laboratory professionals because they know actual and needed lab capacity.<br><br>DLS made recommendation for the harmonisation document to be reviewed and updated because it is outdated; the document is implemented in country but there are also changes in the system that have overtaken that document. For example: ‘In 2015 PCR were probably done maybe at provincial levels but as we speak now PCR specially for COVID is being done even at a district level. So actually things have changed on the ground, we are now ahead on what we have in our ED or in our harmonization document so we also need to update it so that it mirrors or reflects what is currently happening at the moment.’ [that document is like an NEDL]. It is on the table: ‘we started making a lot of noise about . It is in the DLS workplan for this year, but funding holds back the process. [2022].

		They will involve all technical and funding partners who all have lab focal persons who are lab scientists. The provincial people and people in health facilities are important because they know actual lab capacity and needed capacity.
	Perception of usefulness of NEDL in solving (some) barriers to availability of IVDs in tiers / lab system	With the NEDL DLS can lobby for resources and standardize procurement of tests and equipment and human resource placement. (in fact the harmonisation document is more extensive than an NEDL)<br>The NEDL is a help to DLS to lobby for the resources that are required in the EDL but on its own I don't think it will solve the challenges in the lab system.<br>DOC1: Problems that standardization and harmonization respond to are: uncontrolled influx of sub-standard diagnostic instruments resulting in high cost-per-test, limited instrument maintenance contracts, uncoordinated vertical procurements, low instrument utilization, inappropriate human resource placements, and loss of clients' confidence in diagnostic testing services. In the meantime, there is growing need for laboratory services to respond to program scale-ups due to changing dynamics of program targets.
	Present and foreseen challenges in development of NEDL	Funding holds back the process of reviewing the harmonization document
**Implementation of NEDL**	Plans and steps for implementation	Multilayered process to get the appropriate IVDs in the country
	Which stakeholders are /will be involved in implementation	National microbiology Reference lab evaluates / approves different diagnostics.<br>Directorate or the National Pharmaceutical warehouse takes up tendering process for procurement of IVDs that have been approved. Criteria: prices; regulatory requirements for each of the suppliers; prequalified products from procurement partners, for example Global Fund.<br>Selecting IVDs and materials is a multi-layered approach:<br>National microbiology Reference lab starts with the evaluation of these different diagnostics. Directorate or the National Pharmaceutical warehouse takes up tendering process for procurement of IVDs that have performed successfully. Criteria are also the prices among those test kits which have been approved, they also look at the other regulatory requirements for each of the suppliers and then they come up with the ones that can be procured for use in the country. Also they look at prequalified products from procurement partners, for example Global Fund who share their prequalification list; you have to choose like for example let's say they are 3 different types of Syphilis kits that are prequalified. So prequalification is also another requirement. If a kit was evaluated locally it has to be prequalified and screened through all the partners especially Global fund.<br>Manufacturers or their local representatives come to showcase their products. Usually they will have like a scientific evening where they invite potential customers and then they explain their new products.
	What problems in lab services will/may be solved with successful implementation of NEDL?	
	(Foreseen/possible) Problems in implementation of NEDL and how to solve these problems	

**Recommendations**	For own country: steps to take in developing and implementing NEDL by tier	Revise the harmonization document (but director DLS has to initiate). Study the WHO EDL guidelines and compare with harmonization document.<br>Revise harmonization document – director has to initiate and motivate [we try to interview the director, but he is not responsive]<br>Study the WHO EDL guidelines – and see what is new, in comparison with harmonization document.
	For other countries: steps to take in developing NEDL by tier *(Nigeria's lessons)*	
	For development of NEDL: considering gender of recipients	
	For ASLM / FIND/ WHO: type of support for countries in development and implementation of NEDL	Technical support in using WHO EDL in revision of harmonization document and how to implement. Financial support for meetings of stakeholders and for printing and dissemination.<br>Financial to convene the different stakeholders to actually sit down and I revise the harmonization document and come up with the EDL and for printing and dissemination once in it's been developed and yeah.<br>Technical: in developing the guideline as well as even during implementation and how best do we ensure that we've actually implemented everything that we've listed as part of that EDL
