# Supplementary figures and images for: Contexts for developing of national essential diagnostics list. Lessons from a mixed-methods study of existing documents, stakeholders and decision making on tier-specific essential in-vitro diagnostics in African countries
Source: PLOS Glob Public Health. 2023 May 18;3(5):e0001893. doi: 10.1371/journal.pgph.0001893 (PMC10194858; doi:10.1371/journal.pgph.0001893)

S7 Figure. Flow chart sampling of documents

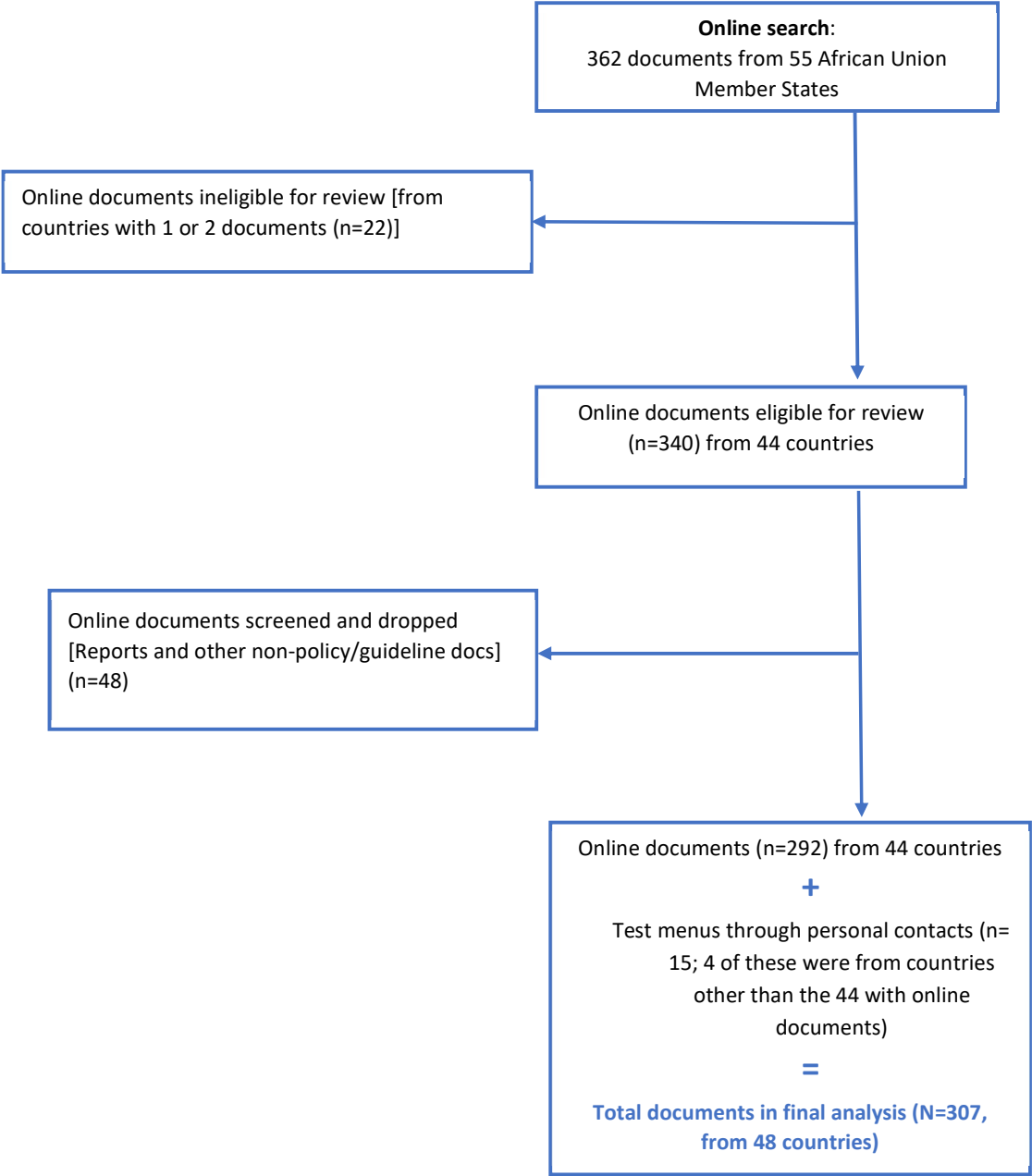

Supplement: S1 Fig — (PDF) [file pgph.0001893.s007.pdf]
