# Supplementary material for: Contexts for developing of national essential diagnostics list. Lessons from a mixed-methods study of existing documents, stakeholders and decision making on tier-specific essential in-vitro diagnostics in African countries
Source: PLOS Glob Public Health. 2023 May 18;3(5):e0001893. doi: 10.1371/journal.pgph.0001893 (PMC10194858; doi:10.1371/journal.pgph.0001893)
Supplement: Attachment — Submitted filename: Response to reviewers.docx [file pgph.0001893.s008.docx]

**Response to reviewers**

Dear reviewers,

Thank you for your close reading of our manuscript, your words of appreciation, and for your comments and useful suggestions to improve the manuscript.

You will see that we added 7 supplementary files to address your requests for data and overview. .

In the table below we address in column 2 each of your comments and questions.

Kind regards,

Winny Koster

| **Issues raised by reviewers** | **Authors’ response** |
| --- | --- |
| 1. Please review your reference list to ensure that it is complete and correct. If you have cited papers that have been retracted, please include the rationale for doing so in the manuscript text, or remove these references and replace them with relevant current references. Any changes to the reference list should be mentioned in the rebuttal letter that accompanies your revised manuscript. If you need to cite a retracted article, indicate the article’s retracted status in the References list and also include a citation and full reference for the retraction notice. | We have reviewed our reference list.  We did not retract papers but have added three (# 21, 22, 23) to strengthen the discussion |
| 2. Please send a completed 'Competing Interests' statement, including any COIs declared by your co-authors. If you have no competing interests to declare, please state "The authors have declared that no competing interests exist". Otherwise please declare all competing interests beginning with the statement "I have read the journal's policy and the authors of this manuscript have the following competing interests:" | We have completed the ‘competing interests’ statement |
| **3**. Please amend your detailed Financial Disclosure statement. This is published with the article. It must therefore be completed in full sentences and contain the exact wording you wish to be published.  a. State the initials, alongside each funding source, of each author to receive each grant.  b. State what role the funders took in the study. If the funders had no role in your study, please state: “The funders had no role in study design, data collection and analysis, decision to publish, or preparation of the manuscript.”  c. If any authors received a salary from any of your funders, please state which authors and which funders. | We have amended our financial disclosure statement |
| 4. In the online submission form, you indicated that "The quantitative database and analysis can be accessed by requesting the authors working for ASLM. The qualitative data transcripts and analysis can be accessed through the first (corresponding) author". All PLOS journals now require all data underlying the findings described in their manuscript to be freely available to other researchers, either 1. In a public repository, 2. Within the manuscript itself, or 3. Uploaded as supplementary information.  This policy applies to all data except where public deposition would breach compliance with the protocol approved by your research ethics board. If your data cannot be made publicly available for ethical or legal reasons (e.g., public availability would compromise patient privacy), please explain your reasons by return email and your exemption request will be escalated to the editor for approval. Your exemption request will be handled independently and will not hold up the peer review process, but will need to be resolved should your manuscript be accepted for publication. One of the Editorial team will then be in touch if there are any issues. | We have uploaded the quantitative and qualitative data bases as supplementary information. S4 and S5  Please note that in the summary and analysis of qualitative interviews (S5) the respondents have been anonymized. |
| 5. We noticed that you used "data not shown" in the manuscript. We do not allow these references, as the PLOS data access policy requires that all data be either published with the manuscript or made available in a publicly accessible database. Please amend the supplementary material to include the referenced data or remove the references. | We added S6 to support the statistical analysis |
| Reviewer #1: Koster et al conducted a mixed-methods study aimed at collecting information from existing national guidance documents from different African countries and on decision making when selecting tier-specific IVDs that could be the basis for recommendations to supporting countries in developing an NEDL or a similar guidance document. They examined xxx documents from 48 countries and conducted key informant interviews with 43 participants from 7 countries. Based on this they provide recommendations concerning development of the NEDL, including a community tier in the NEDL, embedding the NEDL into the national laboratory system context, and stakeholder involvement. These recommendations will be useful as more countries are expected to adapt the WHO EDL to suit national needs. | **NA** |
| Introduction  Line 65: ….unique set of  Line 69: Replace ‘treatment’ with ‘execution’ or ‘implementation’ or other suitable word.  Line 81: Remove ‘A’.  Line 82: I believe the word should be ‘means’ and not ‘makes’  Line 88: Remove the parenthesis from the word ‘sufficiently’  Line 111: The abbreviation UHC was not stated in Line 68 where Universal Health Coverage was first used | Thank you for the close reading.  We have made the corrections you suggested |
| Materials and methods  Lines 195-204: Line 50: It appears the number of documents reviewed were 293, however in line 50, from the abstract, it is stated that ‘362 documents from 48 African countries were reviewed….’. A flow chart showing the document elimination and selection process and a breakdown of document types would be useful. | Thank you for your observations and suggestions.  Supplementary file S7 contains the flow chart.  We added Table 1 with breakdown of document categories in the manuscript text  We corrected the numbers in the abstract |
| A supplementary reference list of the URLs for those documents which could be accessed online should be provided to satisfy the journal’s data availability requirements. | Thank you for this comment. In supplementary materials S1, S2, and S3 are the documents with the URLs as far as available. |
| Discussion  Authors should create a Figure Box or table with the recommendations enabling them to be seen at a glance. | Thank you for the useful suggestion. We have transferred the bullet points under each paragraph of the Discussion to Table 8 with recommendations at the end of the discussion.  You will note that we have strengthened the discussion. |
| Reviewer #2: I must express my sentiments of gratitude to the editor for inviting me for this peer-review, and also appreciate the authors for putting in so much effort in this work. The problem statement was well-defined and highlights the current gaps in existing essential diagnostic list, which not in tune with the current disease burden with respect to the African countries studied, as well as uncovering the limitations in terms of compliance with regulatory guidelines and the various factors contributing to this. The authors showed an in-depth literature review of the current state of NEDL in Africa. Study objectives were also clearly stated WITHOUT ambiguity. | Thank you for your appreciation of our work |
| Are there specific relatable instances (with statistics) where the outcome of a healthcare management program was impacted by the lack of a robust NEDL or test menus? For example, in the management of outbreaks such as cholera, Ebola, COVID-19, etc. This will further drive home the need to address the problem statement. | Thank you for your interesting question. The authors have no knowledge of studies to this end. It would be very interesting to study the lack of a robust NEDL on programmes. |
| In the Materials and Methods section, particularly in the Sampling and sample size of countries section (from lines 191 – 206); Will it be possible to present the distribution by category of all the different documents sourced using a simple table or a chart, so readers can get a snapshot of the information, in addition to the written text? | Thank you for your useful suggestion.  Supplementary file S7 contains the sampling flow chart.  We added Table 1 with breakdown of document categories in the manuscript text  All documents we used are in S1, S2, and S3 |
| The result of the study clearly uncovered the strengths and weaknesses of the different current NEDL and test menu documents in the countries studied, as well as the factors responsible for the gaps in development and implementation. All recommendations made in the discussion were based on the results: such as embedding the NEDL in the national laboratory system context, especially because the test menus (which were country specific) were found to be easily operationalized and in tune with the strengths and weaknesses of country’s health system, the inclusion of relevant stakeholders with decision making power and influence in terms of operationalization and compliance in the formulation of the policy documents.  However, in addition to the recommendations, will it be possible to include example of how other LMICs from other continents (e.g Asia, South America, etc) have managed their NEDL and their current status as far as effectiveness. This can serve as a benchmark for African governments and policy makers to measure how well their countries have performed in terms of implementation of IVDs or how much work needs to done in order to address these deficiencies. | As far as we know, apart from Nigeria in Africa, only India has an NEDL (2019). <https://main.icmr.nic.in/sites/default/files/guidelines/NEDL_2019.pdf>  We did not find any reports or studies on implementation of the Indian NEDL. |
